# Guanidium Unmasked: Repurposing Common Amide Coupling Reagents for the Synthesis of Pentasubstituted Guanidine Bases

**DOI:** 10.1021/acs.joc.4c02645

**Published:** 2025-02-11

**Authors:** Juhana A. S. Aho, Jere K. Mannisto, Saku P. M. Mattila, Marleen Hallamaa, Jan Deska

**Affiliations:** Department of Chemistry, University of Helsinki, Helsinki 00560, Finland

## Abstract

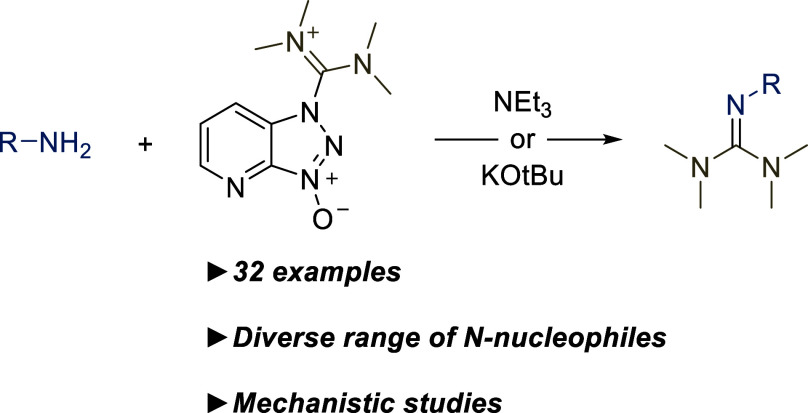

Guanidines make up a class of compounds with important applications in catalysis and medicinal chemistry. In this systematic study, we report on the guanylation of aliphatic amines, anilines, (sulfon)amides, ureas, and carbamates by repurposing HATU, a common amide coupling reagent. The products are 2-substituted 1,1,3,3-tetramethylguanidines (TMGs), a group of sterically hindered superbases. The reaction of a guanidinium salt with aliphatic amines has been regarded as an unwanted side-reaction in amide coupling, yet the exact mechanistic details have been unclear. Our mechanistic investigation shows that the guanylation is highly dependent on the nature of the nitrogen nucleophile. Our findings were applied on two fronts: minimizing guanylation in competing amide coupling reactions as well as maximizing guanylation in a simple one-step synthesis of a broad variety of 2-substituted TMGs, including the late-stage functionalization of pharmaceuticals.

## Introduction

**Scheme 1:**
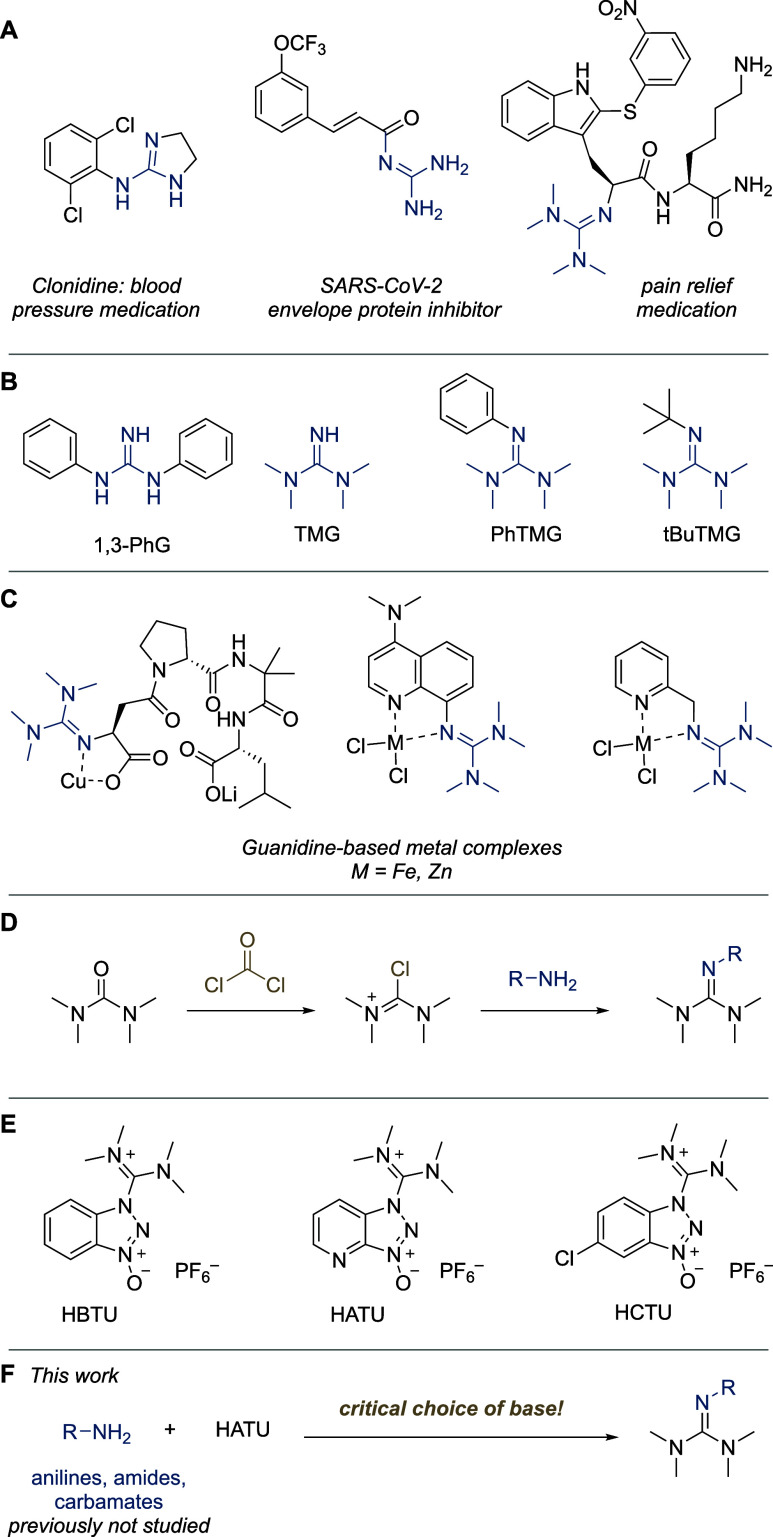
Selected Examples of Important TMGs (A),^1−5^ Guanidines with Modified Substituents (B),^7,8^ Guanidine-based Metal Complexes (C),^14−18^ Conventional Synthesis of TMGs (D),^19−21^ Common Guanidinium Reagents (E), and HATU as General Guanylation Reagent (F)

The guanidine functionality is an important structural motif present in a plethora of bioactive molecules.^1,2^ The moiety is a strong base, and under physiological conditions, it becomes protonated, enhancing protein-interactions and aqueous solubility. Consequently, guanidine functionalities are encountered in a variety of pharmaceuticals with distinct substitution patterns (Scheme 1A).^1−5^ In freebase form, guanidines have diverse uses as superbasic catalysts and stoichiometric reagents.^6^ The guanidine substituents can be tailored to give specific reactivity (Scheme 1B).^7,8^ This approach has been applied particularly to 2-substituted 1,1,3,3-tetramethylguanidines (TMGs). For example, 1,3-PhG, TMG, and *t*BuTMG were evaluated as catalysts in SuFEx click chemistry, but *t*BuTMG uniquely displayed the desired reactivity.^9^ A recent study surveyed a selection of superbase catalysts in glycidol carboxylation, with *t*BuTMG possessing optimal sterics and basicity.^10^ The sterics and basicity of TMGs play important roles in carbon dioxide-based chemistry.^11−13^ Another important application of TMGs is within organometallic chemistry as ligands (Scheme 1C). Copper complexes have been used for enantioselective C–H functionalizations, whereas iron and zinc complexes are used in polymerizations.^14−18^

Conventionally, TMGs are accessed from treatment of tetramethylurea with phosgene or oxalyl chloride, followed by addition of the desired nitrogen nucleophile (Scheme 1D).^19−21^ While effective, the hazardous reagents and the requirement for inert conditions limit the synthetic utility of this approach. The quest for alternative routes for guanylation is therefore an active research topic, where recent methods have focused on synthesizing mono- or disubstituted guanidines.^22−24^ In contrast, we were interested in pentasubstituted guanidines, i.e. TMGs. In search of a more benign protocol in order to overcome the challenges of the conventional phosgene-based method, we were attracted to reports from the field of peptide coupling, where TMGs have been encountered as an undesired side product in amide coupling reactions with guanidium-type coupling reagents HBTU, HATU and HCTU (Scheme 1E).^25^ In the amide coupling process, the typical desired outcome is that the guanidinium reagent reacts with a carboxylic acid. However, highly nucleophilic amines can attack the coupling reagent directly, leading to the formation of the corresponding guanylated amine. Miller and co-workers used HBTU in the synthesis of peptide-based TMGs for catalytic applications,^14,15,26,27^ and initial mechanistic studies found that aliphatic amines are guanylated faster using HATU than HBTU.^3,28^ However, it remained unclear if this trend applied to other, less nucleophilic, nitrogen species. Interestingly, weakly nucleophilic sulfonamides were reported to readily undergo guanylation by HBTU.^29^ This seemed to contradict a more recent report, which found that HATU and HBTU were unreactive toward most nucleophiles.^30^ Despite this side reaction being well-known, we are not aware of a systematic study surveying the role of the reaction conditions in maximizing the guanylation yield. We sought to answer the question if low-reactivity nitrogen nucleophiles could be activated for guanylation by an appropriate tuning of the reaction conditions.

Herein, we report a remarkably simple procedure to access a variety of 2-substituted TMGs from nitrogen nucleophiles, including weakly nucleophilic anilines and amides, previously thought to be unreactive (Scheme 1F).^30^ Our mechanistic results provide strategies for maximizing guanylation, as well as minimizing it in conventional amide coupling.

## Results and Discussion

We began our study by choosing 4-fluoroaniline **1a** as a moderately nucleophilic model substrate. The guanidium-type reagents HBTU, HCTU, and HATU were selected as the tetramethylguanidine donor. Previous literature reports, employing aliphatic amines as substrates, had indicated that HATU forms the guanylated products faster than HBTU in DMF.^28^ Applying literature conditions, we found the reported trend to also apply for **1a** (Table 1, entries 1–4). Guanylation using HATU was largely unaffected by the choice of solvent, whereas HBTU reacted much more slowly in acetonitrile (ACN). The use of triethylamine (TEA) as a base was critical, as the yields were significantly decreased in its absence. An exception to this general pattern was HATU in DMF (Table 1, entry 2), which did not seem to require the addition of base. Subsequently, we proceeded to study HCTU (entries 5 and 6), which behaved similarly to HATU (entries 1 and 2). This suggests that the superiority of HATU and HCTU over HBTU is likely electronic in origin. For a detailed list of screened conditions, including solvent screening, see Supporting Information. Next, we proceeded to further optimize the guanylation yield using HATU in ACN. Surprisingly, a catalytic amount of triethylamine (Table 1, entry 7) and a stoichiometric amount (1.0 equiv; Table 1, entry 8) gave a similar result. These results suggest that TEA has an activating effect in the reaction, possibly on nucleophile **1a**. However, an excess of TEA seemed to be beneficial (Table 1, entry 1). The influence of water on the reaction was confirmed by a stoichiometric amount of HATU being consumed (Table 1, entry 9). A further increase in the amount of HATU finally resulted in nearly quantitative yields (Table 1, entries 10 and 11).

**Table 1:**
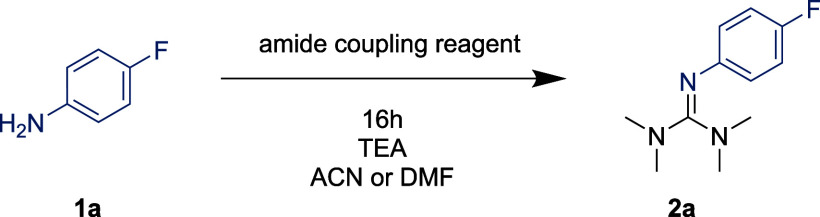
Guanylation Optimization Using *p*-Fluoroaniline **1a**^a^

entry	reagent^b^	solvent	TEA (equiv)	yield of **2a** (%)
1	HATU	ACN	2.0	77 (9)^c^
2	HATU	DMF	2.0	78 (81)^c^
3	HBTU	ACN	2.0	17 (0)^c^
4	HBTU	DMF	2.0	59 (18)^c^
5	HCTU	ACN	2.0	68 (0)^c^
6	HCTU	DMF	2.0	83 (62)^c^
7	HATU	ACN	0.2	70
8	HATU	ACN	1.0	71
9	HATU	ACN	2.0	0^d^
10	HATU (1.2)	ACN	2.0	81
11	HATU (1.5)	ACN	2.0	99

a Yields determined by GC-FID using mesitylene as an internal standard.

b 1.0 equiv, unless mentioned otherwise.

c In the absence of TEA.

d With 2.0 equiv water.

We proceeded to screen whether the reaction could be extended to weaker nucleophiles. To this end, 4-nitroaniline **1b** was chosen as the model substrate (Table 2). Indeed, **1b** showed zero guanylation using TEA (entries 1 and 2). Several nonionic organic bases were screened, but these gave only trace yields at best (see Supporting Information). Gratifyingly, KO*t*Bu in DMF was a major breakthrough (entry 3). Increasing the amount of KO*t*Bu proved to be highly beneficial (Table 2, entry 4), yet, performing the reaction in ACN resulted in a complex mixture (Table 2, entry 5). As KO*t*Bu did not fully dissolve in DMF, 18-crown-6 was trialed in a stoichiometric amount, but it did not affect the reactivity (see Supporting Information). Increasing the amount of HATU to 1.5 equiv resulted in a lower yield (entry 6), however, this could be compensated for by a further increase of the amount of KO*t*Bu, which gave the best result (entry 7). Organic base DBU gave only trace amounts of **2b** (entry 8). Likewise, KHMDS was ineffective (entry 9), however, NaH gave **2b** in a good yield (entry 10). Considering the safety issues associated with NaH in DMF, we advocate for the use of KO*t*Bu.^31^

**Table 2:**
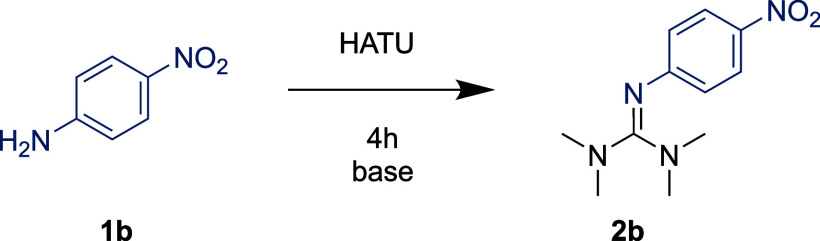
Guanylation Optimization Using Nitroaniline **1b**^a^

entry	solvent	base^b^	yield of **2b** (%)
1	ACN	TEA (2.0)	0
2	DMF	TEA (2.0)	0
3	DMF	KO*t*Bu (1.0)	9
4	DMF	KO*t*Bu (2.0)	63
5	ACN	KO*t*Bu (2.0)	40^c^
6	DMF^d^	KO*t*Bu (2.0)	45
7	DMF^d^	KO*t*Bu (3.0)	70
8	DMF	DBU (2.0)	3
9	DMF	KHMDS (2.0)	0
10	DMF	NaH (2.0)	68

a Yields determined by GC-FID using mesitylene as an internal standard. 1.0 equiv HATU, unless stated otherwise.

b Equivalents in parentheses.

c Complex mixture.

d HATU 1.5 equiv.

**Figure 1:**
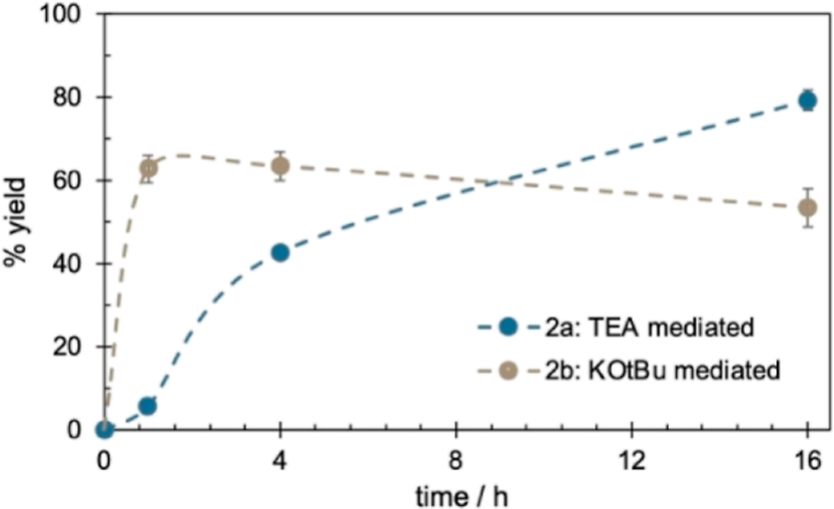
Guanidine formation over time. Conditions: **1a** (0.5 mmol), TEA (2.0 equiv), HATU (1.0 equiv) in ACN, or **1b** (0.5 mmol), KO*t*Bu (2.0 equiv), HATU (1.0 equiv) in DMF. Yields determined by GC-FID using mesitylene as an internal standard.

The optimization results above were elucidated further by a kinetic study (Figure 1), revealing that the two methods proceeded at vastly different rates. The KO*t*Bu-mediated reaction reached full conversion in 60 min. In contrast, the TEA-based reaction took substantially longer, with full conversion taking up to 16 h. The TEA-mediated reaction had a short induction period caused by residual moisture reacting with HATU.

**Scheme 2:**
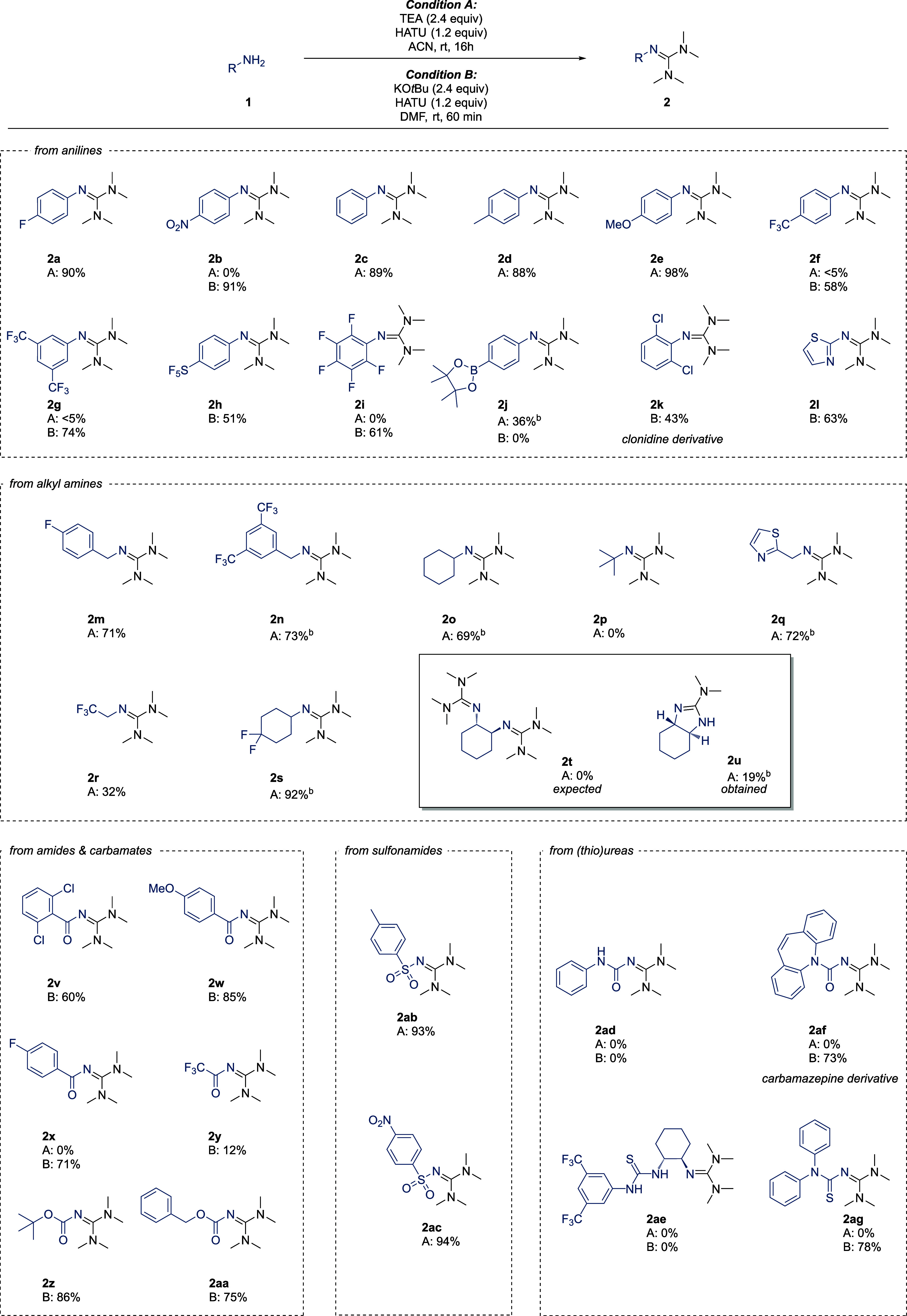
Substrate Scope of the Guanylation Reaction Yields (%) refer to isolated product on 2 mmol scale, except for compounds listed as <5%. For these, trace amounts were observed by GC-MS, but isolation was not attempted. In DMF.

We then proceeded to explore the scope of the reaction using various anilines (Scheme 2). The TEA-promoted reaction (condition A) of **1a** readily provided guanidine **2a** in a good isolated yield, whereas electron-deficient **1b** failed to form **2b**. In contrast, the KO*t*Bu-promoted reaction (condition B) readily delivered **2b**. Electron-rich anilines reacted promptly using condition A, providing products **2c**-**e** in excellent yields. Concurrently, electron-deficient anilines failed to react using condition A, but condition B facilitated the formation of guanidines **2f**-**i** in moderate to good yield. Interestingly, guanidine **2j** was only obtained under condition A, likely due to interference between the boronate ester and KO*t*Bu in condition B. We were pleased to find that sterically hindered and electron-deficient **1k**, a challenging combination, readily formed **2k**, a derivative of the adrenoceptor agonist clonidine. Condition B was also well-compatible with the heterocyclic aniline analogue **1l**, yielding **2l** in 63%. We extended our studies to aliphatic amine nucleophiles. Although aliphatic amines are known to react with HATU,^3,28,30^ we would like to underline that guanylation sensitivity to electronics and sterics has not been studied, to the best of our knowledge. In this regard, *p*-fluorobenzylamine **1m** reacted readily in the presence of triethylamine in ACN, as did significantly more electron-deficient **1n**. While the sterically more demanding cyclohexylamine **1o** still reacted smoothly, the method reached its limit with the bulky *tert*-butylamine **1p** where no reaction could be observed. Furthermore, both heterocyclic **1q** as well as the aliphatic fluorinated **1r** and **1s** turned out to be well-compatible with guanylation condition A. Attempts to produce bis-guanylated **2t** from the corresponding 1,2-diamine failed, and instead, the bicyclic **2u** was obtained. This likely occurred through initial guanylation, followed by an intramolecular attack of the neighboring amine (Supporting Information, Section S11). Next, we explored the other end of the nucleophilicity scale with very weak nitrogen-based nucleophiles. Benzamides **1v**–**x** readily reacted under condition B to give the corresponding *N*-acylated guanidines in good yields. Trifluoroacetamide **1y** could only be isolated in low yield, possibly due to decomposition during the workup. Carbamates **1z** and **1aa** were also smoothly guanylated using condition B. On the other hand, sulfonamides **1ab** and **1ac** did not require strong base and excellent yields of the *N*-sulfonylated guanidines were achieved with TEA. Monosubstituted (thio)ureas **1ad** and **1ae** gave no product, most likely due to decomposition (Supporting Information, Section S12). Delightfully, 1,1-disubstituted (thio)ureas **1af** and **1ag** were readily guanylated under condition B.

**Scheme 3:**
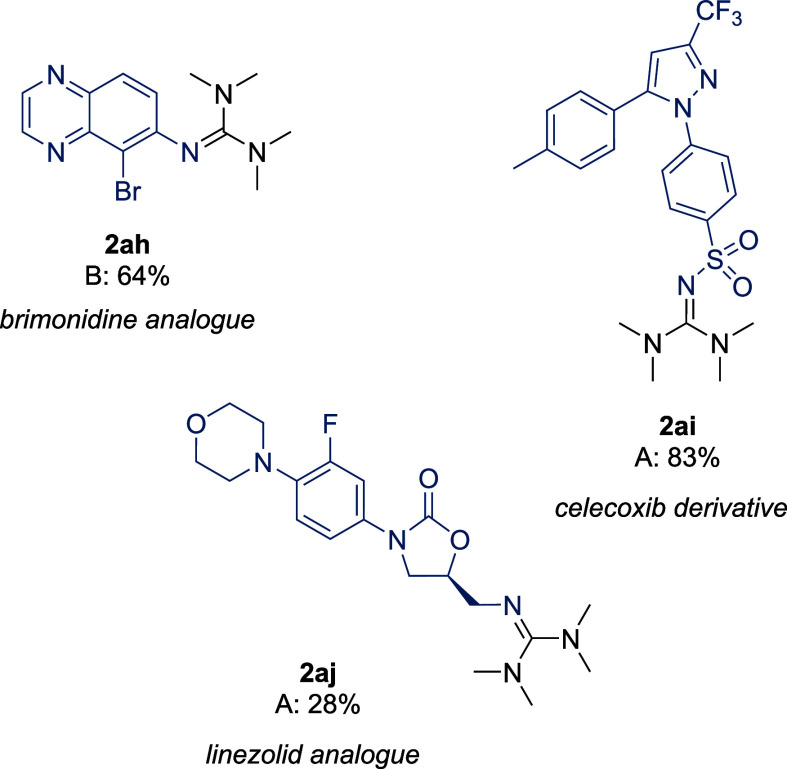
Late-stage Functionalization of Active Pharmaceutical Ingredients and Pharma Precursors Yields (%) refer to isolated product on 2 mmol scale. See Scheme 2 for conditions A and B.

Most basic guanidines were easy to purify by precipitation, where the crude nitrogen base was added to oxalic acid in Et_2_O (see Supporting Information). Subsequent back extraction of the oxalate salt from aqueous sodium hydroxide provided the products in a high NMR purity (>95%) without the need for recrystallization or even chromatographic separation. Lastly, as the guanylation reactions seemed efficient and products were easy to purify, late-stage functionalization was attempted on a selection of more complex amine-containing pharmaceuticals (Scheme 3). We obtained the corresponding TMG derivatives in moderate to good yields, clearly illustrating the suitability of the system for late-stage functionalization in the context of medicinal chemistry.

Having explored the substrate scope, we decided to benchmark our method against conventional synthetic methods.^7,19−21^ We chose the substrates 4-nitroaniline **1b**, fluorinated benzylamine **1n**, and 4-fluorobenzamide **1x**. Tetramethylurea was first treated with oxalyl chloride. Then the nucleophile (1.0 equiv) was added alone or in the presence of TEA (nucleophile 1.0 eq and TEA 1.0 equiv). Only fluorinated benzylamine **1n** gave guanidine products (Supporting Information, Section S10). Guanidine **2b** has previously been synthesized via the conventional route.^7,21^ This suggests our method is more robust and has better reproducibility. In particular, our method is better suited for weakly nucleophilic species, such as benzamides and electron-deficient anilines.

**Figure 2:**
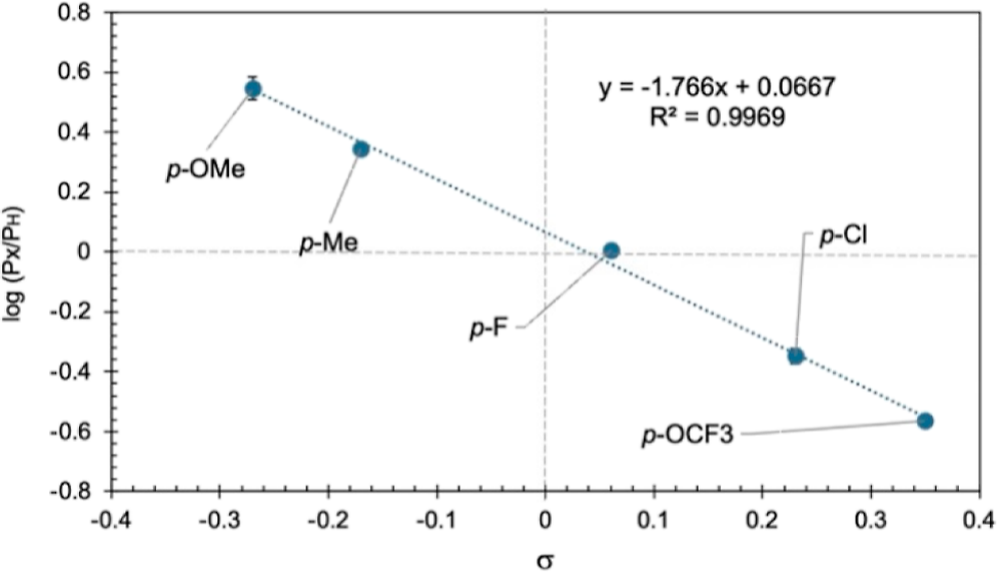
Hammett competition study of 4-substituted anilines under condition A. Reaction conditions: aniline (1.0 equiv), 4-substituted aniline (1.0 equiv), TEA (2.0 equiv), HATU (1.0 equiv), ACN (2.0 mL), 0.5 mmol scale; stirred at room temperature for 16 h.

**Figure 3:**
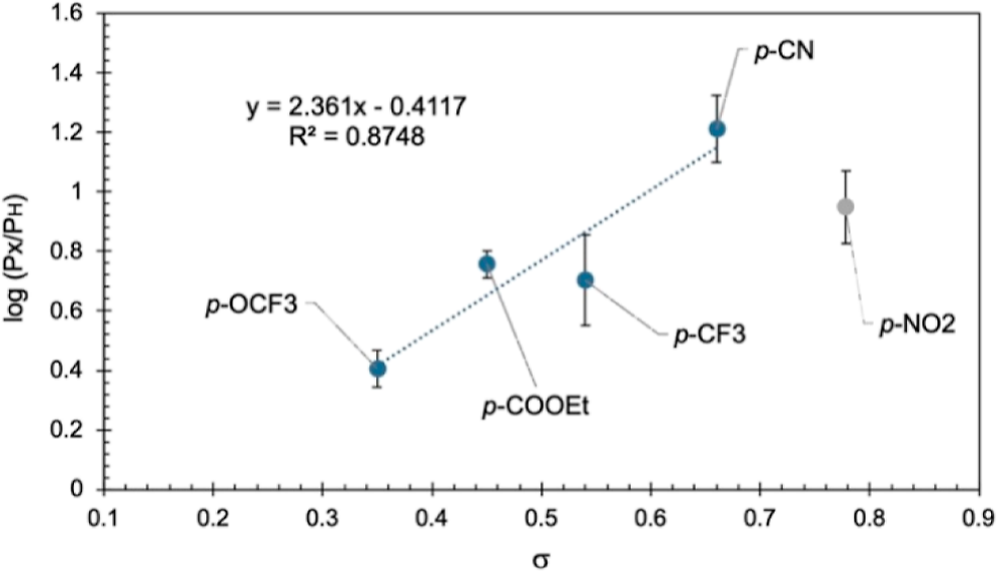
Hammett competition study of 4-substituted anilines under condition B. Reaction conditions: aniline (1.0 equiv), 4-substituted aniline (1.0 equiv), KO*t*Bu (4.0 equiv), HATU (1.0 equiv), DMF (2.0 mL), 0.5 mmol scale; stirred at room temperature for 60 min.

The kinetic studies (Figure 1) and the reaction scope (Scheme 2) suggested that conditions A and B did not share a common reaction mechanism, an apparent fact that was further investigated by competition studies of 4-substituted anilines using Hammett σ parameters (Figures 2 and 3).^32−34^ Under condition A, a negative slope (ρ = −1.77) was recorded, which suggests a buildup of partial positive charge at the aniline nitrogen atom in the transition state (TS). The absolute value of ρ is consistent with an associative mechanism, where the base activates the aniline for nucleophilic attack.^11^ In contrast, under condition B, a positive slope (ρ = +2.36) was obtained, which implies that significant negative charge forms at the aniline nitrogen atom in the TS. The large absolute value of ρ suggests that the charge is localized close to the aromatic ring; hence, the substituents exert a larger influence. The presence of a large negative charge is further supported by good correlation with σ^–^ values (*R*^2^ = 0.93, see Supporting Information). It should be noted that **2b** slightly deviates from the general trend, but this may be due to secondary equilibrium effects exerted by the nitro-substituent.^32^

**Scheme 4:**
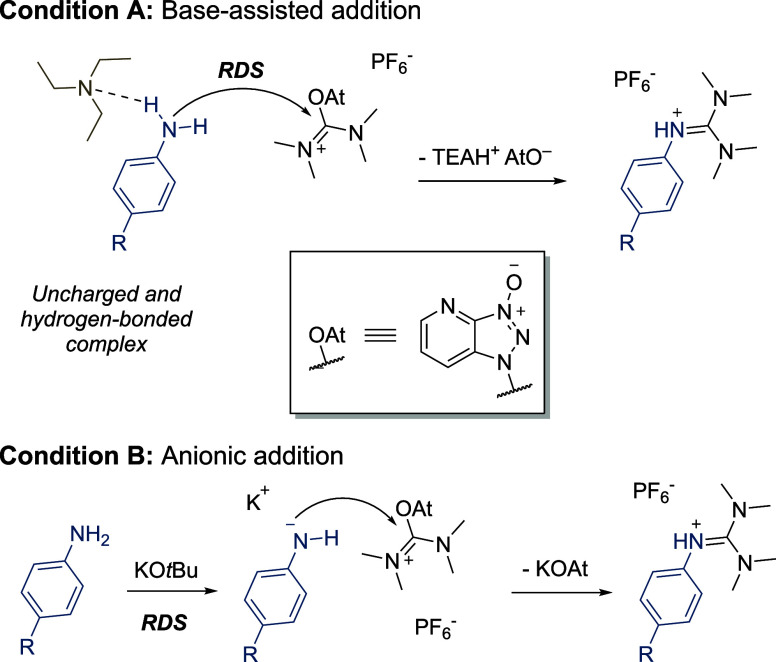
Mechanistic Proposal of the Base-Dependent Guanylation Reactions

The proposed mechanisms are listed in Scheme 4. Under condition A, triethylamine associates to the aniline, activating it for nucleophilic attack. The rate-determining step (RDS) is the addition to HATU, which is likely to occur in a concerted manner with the deprotonation.^11^ The activating effect of triethylamine is further supported by the fact that a catalytic amount of the base is sufficient for synthetically useful yields (Table 1, entry 7). Under condition A, the hydrogen-bonded complex of TEA and an electron-deficient aniline is not sufficiently nucleophilic to directly react with HATU. In contrast, under condition B, deprotonation of the aniline is the RDS. The anion is significantly more nucleophilic, and once formed, it will rapidly add to HATU under substitution of the oxyazabenzotriazole anion (OAt).

Having studied factors that promote guanylation, we pondered if reaction conditions could be tuned to suppress guanylation in amide couplings, a common side reactivity and source of impurities of this industrially highly relevant C–N bond forming reaction. Considering that condition A involves a base-assisted addition of aniline to HATU (Scheme 4), we were curious whether alternative bases with more bulk than TEA could prevent guanylation. In this regard, DIPEA, DIPA, TMP, and PMP were tested (Table 3, entries 2–5). These bases have p*K*_BH_ values similar to those of TEA (ca. 18 in ACN) but are sterically more hindered. We found that the guanylation yield was only marginally affected by the increase of the sterics of the base. Less basic pyridine was explored next, and we observed that guanylation yield was reduced by nearly an order of magnitude (entry 6), and the more hindered 2,6-lutidine and 2,6-di*tert*-butylpyridine inhibited guanylation further (entries 7 and 8). In contrast, DMAP, similar in basicity to TEA, gave a high guanylation yield (entry 9). During optimization, we observed that guanylation proceeded without an external base, although in low yield, suggesting that aniline **1a** may act as a base (Table 1, entry 1). Consequently, we also studied 4-methoxy-*N*,*N*-dimethylaniline, similar in basicity to pyridine, but more hindered than **1a** (Table 3, entry 10). In this case, no guanylation was observed. In summary, our results imply that sterically hindered weaker bases (p*K*_BH_ ≤ 14) do not mediate guanylation and could therefore be a smart choice in HATU-induced coupling reactions to avoid guanidine-related impurities.

**Table 3:**
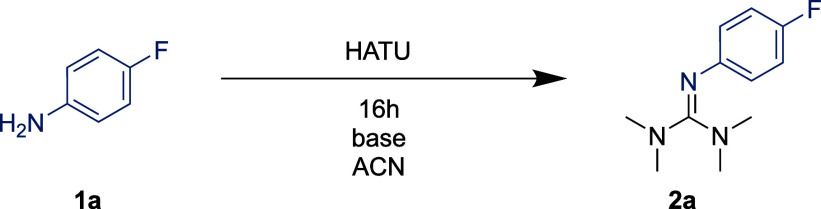
Base Screening in the Guanylation of **1a** Using HATU in ACN

entry	base	p*K*_BH_ in ACN^a^	yield of **2a** (%)
1	triethylamine	18.83	77
2	*N*,*N*-diisopropylethylamine	18.60	64
3	*N*,*N*-diisopropylamine	18.81	52
4	2,2,6,6-tetramethylpiperidine	18.64	44
5	1,2,2,6,6-pentamethylpiperidine	18.62	54
6	pyridine	12.53	17
7	2,6-lutidine	14.16	11
8	2,6-di(*t*Bu)pyridine	8.05	0
9	4-(*N*,*N*-dimethylamino)pyridine	17.95	47
10	4-methoxy-*N*,*N*-dimethylaniline	12.72	0

a Refs.^35–40^

In order to validate this side observation, the results of the base screen were applied in an amide coupling study using 4-fluorobenzoic acid **3a** (Table 4). The intermolecular competition between carboxylic acid **3a** and aniline **1a** was probed with both HATU and HBTU. Using TEA, trace amounts of guanylated byproduct **2a** were observed (entries 1 and 2) while switching to 2,6-lutidine as base effectively suppressed guanylation while preserving very high amidation yields (entries 3 and 4).

**Table 4:**
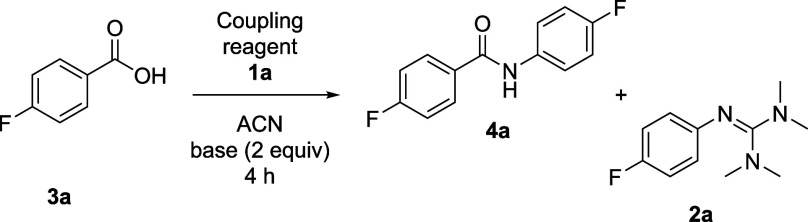
Intermolecular Competition Studies on the Effect of Base in Amidation Reactions^a^

entry	coupling reagent	base	**4a** (%)	**2a** (%)
1	HATU	triethylamine	86	<1
2	HBTU	triethylamine	84	4
3	HATU	2,6-lutidine	87	0
4	HBTU	2,6-lutidine	83	0

a Yields determined by GC-FID using mesitylene as an internal standard.

We then proceeded to a more challenging scenario of both intermolecular and intramolecular competition with carboxylic acid **3b** (Table 5). Previous work had reported **3b** to undergo exhaustive guanylation of the sulfonamide using HBTU and *N*,*N*-diisopropylethylamine, similar in its basicity as TEA but a more hindered base, in CH_2_Cl_2_, producing **6b** as the main product.^29^ In our hands, HATU and TEA did yield the desired amide **4b**, yet with small amounts of guanidine **2a** (entry 1). Quantitative ^19^F{^1^H} NMR showed an additional two minor peaks at −118.32 and −118.59 ppm. The former and more abundant one was assigned to **6b** based on literature reactivity.^29^ While we cannot rule out formation of **5b**, it is clear from ^1^H NMR results that **5b** is not formed in significant quantities (see Supporting Information, Section 16). Similar to the simple amide coupling test, changing the base to 2,6-lutidine not only improved amidation yield but also eliminated the impurities at −118.32 ppm (**6b**) and −118.59 ppm (entry 2).

**Table 5:**
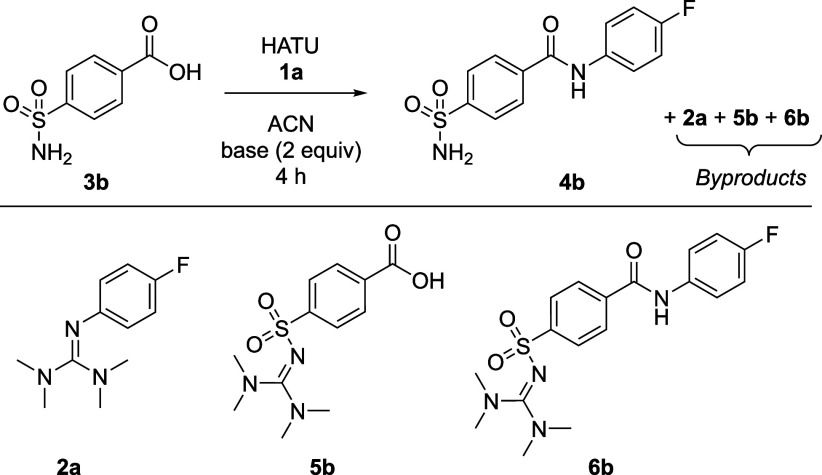
Intramolecular and Intermolecular Competition Studies on the Effect of Base in Amidation reactions^a^

entry	base	**4b** (%)	**2a** (%)	**6b** (%)	unknown (%)
1	triethylamine	23	8	3	2
2	2,6-lutidine	44	5	0	0

a Yield was determined with ^19^F{^1^H}-NMR using α,α,α-trifluorotoluene as an internal standard.

## Conclusion

In summary, we have shown HATU to be an efficient guanylation reagent. Two complementary methods were developed to form 2-substituted TMGs with a wide substrate scope and good isolated yields under mild conditions. Our HATU-based system was shown to be well-suited for late-stage functionalization of pharmaceuticals, possessing superior reactivity over conventional methods. The appropriate base must be chosen by taking the nature of the nucleophile into account. Strong nucleophiles are optimally paired with mildly basic TEA and react via uncharged intermediates. In contrast, weak nucleophiles are not sufficiently activated by TEA, but deprotonation using KO*t*Bu, a strong base, results in fast guanylation. The lessons learned in the optimization of the guanylation reaction were applied to improve the chemoselectivity of amide couplings when using guanidium-based coupling reagents.

## Data Availability

The data underlying this study are available in the published article and its Supporting Information, and openly available in Zenodo at 10.5281/zenodo.14652609.

## Supporting Information Available

The electronic Supporting Information provide general information, general experimental procedures, reaction optimization, synthetic methods, compound characterization, and NMR spectra (PDF)

## References

[ref1] Kim S.-H.; Semenya D.; Castagnolo D. Antimicrobial drugs bearing guanidine moieties: A review. Eur. J. Med. Chem. 2021, 216, 113293 10.1016/j.ejmech.2021.113293. 33640673

[ref2] Wexselblatt E.; Esko J. D.; Tor Y. On Guanidium and Cellular Uptake. J. Org. Chem. 2014, 79, 6766–6774. 10.1021/jo501101s. 25019333 PMC4120969

[ref3] del Fresno M.; El-Faham A.; Carpino L. A.; Royo M.; Albericio F. Substituted Guanidines: Introducing Diversity in Combinatorial Chemistry. Org. Lett. 2000, 2, 3539–3542. 10.1021/ol006322p. 11073639

[ref4] Somberg N. H.; Sučec I.; Medeiros-Silva J.; Jo H.; Beresis R.; Syed A. M.; Doudna J. A.; Hong M. Oligomeric State and Drug Binding of the SARS-CoV-2 Envelope Protein Are Sensitive to the Ectodomain. J. Am. Chem. Soc. 2024, 146, 24537–24552. 10.1021/jacs.4c07686. 39167680 PMC12227306

[ref5] de Vega J. P.; Garcia-Lopez T.; Zaccaro L.; Royo M.; Albericio F.; Fernandez-Carvajal A.; Ferrer-Montiel A.; Gonzalez-Muniz R. Solid-Phase Synthesis of New Trp(Nps)-Containing Dipeptide Derivatives as TRPV1 Channel Blockers. Molecules 2010, 15, 4924–4933. 10.3390/molecules15074924. 20657400 PMC6257583

[ref6] Puleo T. R.; Sujansky S. J.; Wright S. E.; Bandar J. S. Organic Superbases in Recent Synthetic Methodology Research. Chem.—Eur. J. 2021, 27, 4216–4229. 10.1002/chem.202003580. 32841442

[ref7] Leffek K. T.; Pruszynski P.; Thanapaalasingham K. Basicity of substituted 2-phenyl-1,1,3,3-tetramethylguanidines and other bases in acetonitrile solvent. Can. J. Chem. 1989, 67, 590–595. 10.1139/v89-089.

[ref8] Maji B.; Stephenson D. S.; Mayr H. Guanidines: Highly Nucleophilic Organocatalysts. ChemCatChem 2012, 4, 993–999. 10.1002/cctc.201200143.

[ref9] Smedley C. J.; Homer J. A.; Gialelis T. L.; Barrow A. S.; Koelln R. A.; Moses J. E. Accelerated SuFEx Click Chemistry For Modular Synthesis. Angew. Chem., Int. Ed. 2022, 61, e202112375 10.1002/anie.202112375. PMC8867595 34755436

[ref10] Muzyka C.; Renson S.; Grignard B.; Detrembleur C.; Monbaliu J. M. Intensified Continuous Flow Process for the Scalable Production of Bio-Based Glycerol Carbonate. Angew. Chem., Int. Ed. 2024, 63, e202319060 10.1002/anie.202319060. 38197641

[ref11] Mannisto J. K.; Pavlovic L.; Tiainen T.; Nieger M.; Sahari A.; Hopmann K. H.; Repo T. Mechanistic insights into carbamate formation from CO2 and amines: the role of guanidine-CO2 adducts. Catal. Sci. Technol. 2021, 11, 6877–6886. 10.1039/D1CY01433A.

[ref12] Mannisto J. K.; Sahari A.; Lagerblom K.; Niemi T.; Nieger M.; Sztano G.; Repo T. One-Step Synthesis of 3,4-Disubstituted 2-Oxazolidinones by Base-Catalyzed CO2 Fixation and Aza-Michael Addition. Chem.—Eur. J. 2019, 25, 10284–10289. 10.1002/chem.201902451. 31141227

[ref13] Mannisto J. K.; Pavlovic L.; Heikkinen J.; Tiainen T.; Sahari A.; Maier N. M.; Rissanen K.; Nieger M.; Hopmann K. H.; Repo T. N-Heteroaryl Carbamates from Carbon Dioxide via Chemoselective Superbase Catalysis: Substrate Scope and Mechanistic Investigation. ACS Catal. 2023, 13, 11509–11521. 10.1021/acscatal.3c02362.

[ref14] Morack T.; Myers T. E.; Karas L. J.; Hardy M. A.; Mercado B. Q.; Sigman M. S.; Miller S. J. An Asymmetric Aromatic Finkelstein Reaction: A Platform for Remote Diarylmethane Desymmetrization. J. Am. Chem. Soc. 2023, 145, 22322–22328. 10.1021/jacs.3c08727. 37788150 PMC10591928

[ref15] Chinn A. J.; Kim B.; Kwon Y.; Miller S. J. Enantioselective Intermolecular C–O Bond Formation in the Desymmetrization of Diarylmethines Employing a Guanidinylated Peptide-Based Catalyst. J. Am. Chem. Soc. 2017, 139, 18107–18114. 10.1021/jacs.7b11197. 29116792 PMC5738244

[ref16] Burkart L.; Eith A.; Hoffmann A.; Herres-Pawlis S. Open Loop Recycling – Guanidine Iron(II) Polymerization Catalyst for the Depolymerization of Polylactide. Chem.—Asian. J. 2023, 18, e202201195 10.1002/asia.202201195. 36577118

[ref17] Conrads C.; Burkart L.; Soerensen S.; Noichl S.; Kara Y.; Heck J.; Hoffmann A.; Herres-Pawlis S. Understanding structure–activity relationships: iron(ii) complexes of “Legacy Guanidines” as catalysts for the synthesis of polylactide. Catal. Sci. Technol. 2023, 13, 6006–6021. 10.1039/d3cy01117h.

[ref18] Kröckert K. W.; Garg F.; Heinz M. V.; Lange J.; Simoes P. P.; Schmidt R.; Bienemann O.; Hoffmann A.; Herres-Pawlis S. Understanding the structure–activity relationship and performance of highly active novel ATRP catalysts. Dalton Trans. 2022, 51, 13272–13287. 10.1039/d2dt01954j. 35983714

[ref19] Barton D. H. R.; Elliott J. D.; Gero S. D. Synthesis and properties of a series of sterically hindered guanidine bases. J. Chem. Soc., Perkin Trans. 1 1982, 1, 2085–2090. 10.1039/P19820002085.

[ref20] Wittmann H.; Raab V.; Schorm A.; Plackmeyer J.; Sundermeyer J. Complexes of Manganese, Iron, Zinc, and Molybdenum with a Superbasic Tris(guanidine) Derivative of Tris(2-ethylamino)amine (Tren) as a Tripod Ligand. Eur. J. Inorg. Chem. 2001, 2001, 1937–1948. 10.1002/1099-0682(200108)2001:8<1937::aid-ejic1937>3.0.co;2-i.

[ref21] Walter P.; Kaifer E.; Herrmann H.; Wadepohl H.; Hubner O.; Himmel H.-J. Redox-Active Guanidines with One or Two Guanidino Groups and Their Integration in Low-Dimensional Perovskite Structures. Eur. J. Inorg. Chem. 2019, 2019, 4147–4160. 10.1002/ejic.201900975.

[ref22] An T.; Lee Y. Nucleophilic Substitution at the Guanidine Carbon Center via Guanidine Cyclic Diimide Activation. Org. Lett. 2021, 23, 9163–9167. 10.1021/acs.orglett.1c03473. 34766783

[ref23] Wester A.; Björkling F.; Franzyk H. Evaluation of 1H-Triazole-1-[N,N′-Bis(tert-butoxycarbonyl)]carboxamidine in Solution-Phase and On-Resin Guanidinylation. J. Org. Chem. 2021, 86, 14371–14380. 10.1021/acs.joc.1c00994. 34661410

[ref24] Hickey S. M.; Ashton T. D.; Pfeffer F. M. Facile Synthesis of Guanidine Functionalised Building Blocks. Asian J. Org. Chem. 2015, 4, 320–326. 10.1002/ajoc.201402242.

[ref25] Montalbetti C. A. G. N.; Falque V. Amide Bond Formation and Peptide Coupling. Tetrahedron 2005, 61, 10827–10852. 10.1016/j.tet.2005.08.031.

[ref26] Trampellini N.; Mercado B. Q.; Miller S. J. Scaffold-Oriented Asymmetric Catalysis: Conformational Modulation of Transition State Multivalency during a Catalyst-Controlled Assembly of a Pharmaceutically Relevant Atropisomer. Chem.—Eur. J. 2024, e202401109 10.1002/chem.202401109. 38507249 PMC11132932

[ref27] Kim B.; Chinn A. J.; Fandrick D. R.; Senanayake C. H.; Singer R. A.; Miller S. J. Distal Stereocontrol Using Guanidinylated Peptides as Multifunctional Ligands: Desymmetrization of Diarylmethanes via Ullman Cross-Coupling. J. Am. Chem. Soc. 2016, 138, 7939–7945. 10.1021/jacs.6b03444. 27254785 PMC5127171

[ref28] Albericio F.; Bofill J. M.; El-Faham A.; Kates S. A. Use of Onium Salt-Based Coupling Reagents in Peptide Synthesis. J. Org. Chem. 1998, 63, 9678–9683. 10.1021/jo980807y.

[ref29] Gluszok S.; Goossens L.; Depreux P.; Henichart J.-P. Efficient synthesis of tetramethylsulfonylguanidines between a free sulfonamide group and HBTU. Tetrahedron Lett. 2006, 47, 6087–6090. 10.1016/j.tetlet.2006.06.094.

[ref30] Vrettos E. I.; Sayyad N.; Mavrogiannaki E. M.; Stylos E.; Kostagianni A. D.; Papas S.; Mavromoustakos T.; Theodorou V.; Tzakos A. G. Unveiling and tacknlong guanidium peptide coupling reagent side reactions towards the recent development of peptide-drug conjugates. RSC Adv. 2017, 7, 50519–50526. 10.1039/C7RA06655D.

[ref31] Yang Q.; Sheng M.; Henkelis J. J.; Tu S.; Wiensch E.; Zhang H.; Zhang Y.; Tucker C.; Ejeh D. E. Explosion Hazards of Sodium Hydride in Dimethyl Sulfoxide, *N,N-*Dimethylformamide, and *N,N*-Dimethylacetamide. Org. Process Res. Dev. 2019, 23, 2210–2217. 10.1021/acs.oprd.9b00276.

[ref32] Hansch C.; Leo A.; Taft R. W. A survey of Hammett substituent constants and resonance and field parameters. Chem. Rev. 1991, 91, 165–195. 10.1021/cr00002a004.

[ref33] McKnight E. A.; Arora R.; Pradhan E.; Fujisato Y. H.; Ajayi A. J.; Lautens M.; Zeng T.; Le C. M. BF_3_-Catalyzed Intramolecular Fluorocarbamoylation of Alkynes via Halide Recycling. J. Am. Chem. Soc. 2023, 145, 11012–11018. 10.1021/jacs.3c03982. 37172320

[ref34] Wang M.; Rowshanpour R.; Guan L.; Ruskin J.; Nguyen P. M.; Wang Y.; Zhang Q. A.; Liu R.; Ling B.; Woltornist R.; Stephens A. M.; Prasad A.; Dudding T.; Lectka T.; Pitts C. R. Competition between C–C and C–H Bond Fluorination: A Continuum of Electron Transfer and Hydrogen Atom Transfer Mechanisms. J. Am. Chem. Soc. 2023, 145, 22442–22455. 10.1021/jacs.3c06477. 37791901

[ref35] Kaljurand I.; Kütt A.; Sooväli L.; Rodima T.; Mäemets V.; Leito I.; Koppel I. A. Extension of the Self-Consistent Spectrophotometric Basicity Scale in Acetonitrile to a Full Span of 28 pKa Units: Unification of Different Basicity Scales. J. Org. Chem. 2005, 70, 1019–1028. 10.1021/jo048252w. 15675863

[ref36] Tshepelevitsh S.; Kütt A.; Lokov M.; Kaljurand I.; Saame J.; Heering A.; Plieger P. G.; Vianello R.; Leito I. On the Basicity of Organic Bases in Different Media. Eur. J. Org Chem. 2019, 40, 6735–6748. 10.1002/ejoc.201900956.

[ref37] Rõõm E.; Kütt A.; Kaljurand I.; Koppel I. A.; Leito I.; Koppel I.; Mishima M.; Goto K.; Miyahara Y. Brønsted Basicities of Diamines in the Gas Phase, Acetonitrile, and Tetrahydrofuran. Chem.—Eur. J. 2007, 13, 7631–7643. 10.1002/chem.200700097. 17594707

[ref38] Heldebrant D. J.; Koech P. K.; Rainbolt J. E.; Zheng F.; Smurthwaite T.; Freeman C. J.; Oss M.; Leito I. Performance of single-component CO2-binding organic liquids (CO2BOLs) for post combustion CO2 capture. J. Chem. Eng. 2011, 171, 794–800. 10.1016/j.cej.2011.02.012.

[ref39] Benoit R. L.; Frechette M.; Lefebvre D. 2,6-Di-tert-butylpyridine: an unusually weak base in dimethylsulfoxide. Can. J. Chem. 1988, 66, 1159–1162. 10.1139/v88-190.

[ref40] Traditional Strong and Hindered Bases, can be found under https://www.sigmaaldrich.com/FI/en/technical-documents/technical-article/chemistry-and-synthesis/reaction-design-and-optimization/traditional-strong-and-hindered-bases, 2024 (accessed May 12, 2024).

