# Supplementary material for: Guanidium Unmasked: Repurposing Common Amide Coupling Reagents for the Synthesis of Pentasubstituted Guanidine Bases
Source: J Org Chem. 2025 Feb 11;90(7):2636–43. doi: 10.1021/acs.joc.4c02645 (PMC11852210; doi:10.1021/acs.joc.4c02645)
Supplement: Supplementary file 1 — jo4c02645_si_001.pdf [file jo4c02645_si_001.pdf]

Supporting Information

Guanidium Unmasked: Repurposing Common Amide Coupling Reagents for the Synthesis of Pentasubstituted Guanidine Bases

Juhana A. S. Aho, Jere K. Mannisto,* Saku P. M. Mattila, Marleen Hallamaa, Jan Deska*

Department of Chemistry, University of Helsinki, P.O. Box 55, A.I. Virtasen aukio 1, 00014 Helsinki, Finland.

*Correspondence to:

jere.mannisto@helsinki.fi

jan.deska@helsinki.fi

Table of Contents

1. General Experimental Information…… S3
2. General Protocol for 0.5 mmol scale…… S4
3. Supplementary Figure S1…… S4
4. Supplementary Figure S2…… S5
5. Solvent screening table S1…… S5
6. Expanded screening table S2…… S6
7. Expanded screening table S3…… S7
8. Hammett competition protocol…… S8
9. Hammett competition figure using σ- values…… S8
10. Oxalyl chloride-based route test……S9
11. Product 2u proposed mechanism…… S9
12. Urea 1ad proposed decomposition mechanism…… S10
13. Product nitrogen lone pair isomerization in 1H-NMR…… S10
14. General protocols for 2 mmol scale…… S11
15. Procedures and Analytical Data…… S12
16. Competition experiment…… S73

EXPERIMENTAL SECTION

1. General Experimental Information

All reagents employed were at the highest commercially available purity levels and used as received, unless otherwise specified. Deuterated solvents were purchased at the highest purity level. Solvents were dried over activated 3 Å molecular sieves. Silica gel from Merck (Millipore 60, 40-60 µm, 240-400 mesh) was used for column chromatography. Reaction monitoring was performed via thin layer chromatography (TLC) using precoated silica gel plates from Macherey-Nagel (TLC Silica gel 60 F_{254}). The spots were identified using irradiation with UV-light and a staining solution (basic potassium permanganate solution).

The nuclear magnetic resonance (NMR) characterization of isolated compounds (^{1}H, ^{13}C, ^{19}F) were performed on Bruker Avance Neo 400 (^{1}H-frequency 400 MHz) spectrometer operating with the frequency, deuterated solvent, and at the temperature indicated in parentheses. The Hammett competition experiments, in situ experiments (section 16), and characterization of compounds 4a and 4b were instead performed on a Bruker Avance NEO 500 (^{1}H-frequency 500 MHz) spectrometer. All ^{13}C and ^{19}F spectra were proton-decoupled. The chemical shifts are reported in ppm related to the signal of residual solvent of $CDCl_3$ (^{1}H: ($CDCl_3$) = 7.26 ppm, ^{13}C: ($CDCl_3$) = 77.2 ppm) or DMSO-d_6 (1H: (DMSO-d_6) = 2.50 ppm, 13C: (DMSO-d_6) = 39.52 ppm).

High-resolution mass spectrometry (HRMS) was performed on an Agilent 6230 electrospray ionization time-of-flight (ESI-TOF) spectrometer.

Agilent 7890B GC system with an Agilent 7693 Autosampler was used for gas chromatography using flame ionization detector. The GC-FID was used for reaction optimization for compounds **2a** and **2b**. Fused silica column Agilent 190915-433UI was used with the following parameters: Flow 1.7 ml/min, inlet 300 °C, split ratio 10:1, FID 300 °C, injection 1 µl and the oven temperature program below.

Ramp (°C/min)	Temperature	Hold (min)
	60	0.5
10	100	0
15	200	2
20	300	2

Agilent 6890N GC system equipped with Agilent 7683 autosampler and Agilent 5973N Mass Detector was used for solvent screening and for determining whether condition A or condition B would be better for some compounds for 2 mmol scale. Fused silica column Agilent 190915-433 was used with the following parameters: Flow 1.1 ml/min, inlet 280 °C, split ratio 30:1, injection 1 µl and the oven temperature program below.

Ramp (°C/min)	Temperature	Hold (min)
	60	0.5
10	90	0
20	300	6

2. General protocol for 0.5 mmol scale:

Guanidium reagent (1 eq, 0.5 mmol) is added into a 8 ml vial loaded with a magnetic stir bar. If the amine (1.0 eq, 0.5 mmol) is solid, it is also weighed in. Dry solvent (2 ml) is added and the mixture is stirred until everything dissolves. If the amine (1.0 eq, 0.5 mmol) is liquid, it is added after dissolving the guanidium reagent. Base (2.0 eq, 1 mmol) is added and the reaction is left to run for a desired time.

Workup:

Mesitylene (60 µl) is added into the vial. Toluene (2.5 ml) and 2M NaOH (2.5 ml) are added and the vial is vigorously shaken. From the organic layer either 100 µl (if using ACN as solvent) or 50 µl (when using DMF) is transferred to a 1.8 ml GC vial containing 1.5 ml EtOAc. If there is something insoluble, the resulting mixture is filtered through a PTFE membrane filter. The vial is then analyzed with the described GC method.

3. Supplementary Figure S1:

Calibration curve for GC-FID using isolated product **2a**.

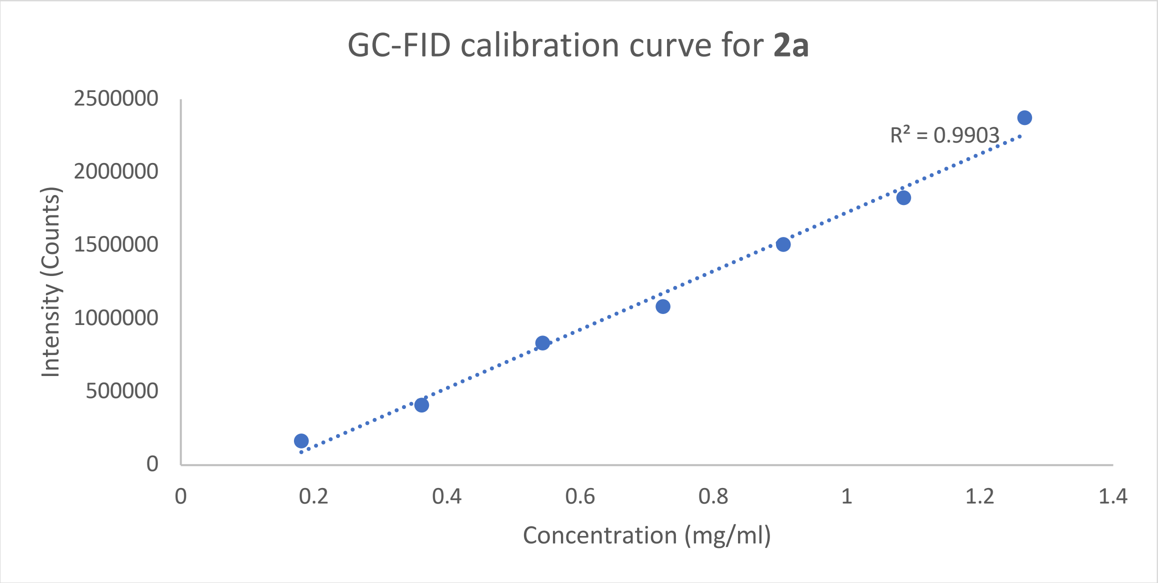

4. Supplementary Figure S2:

Calibration curve for GC-FID using isolated product **2b**.

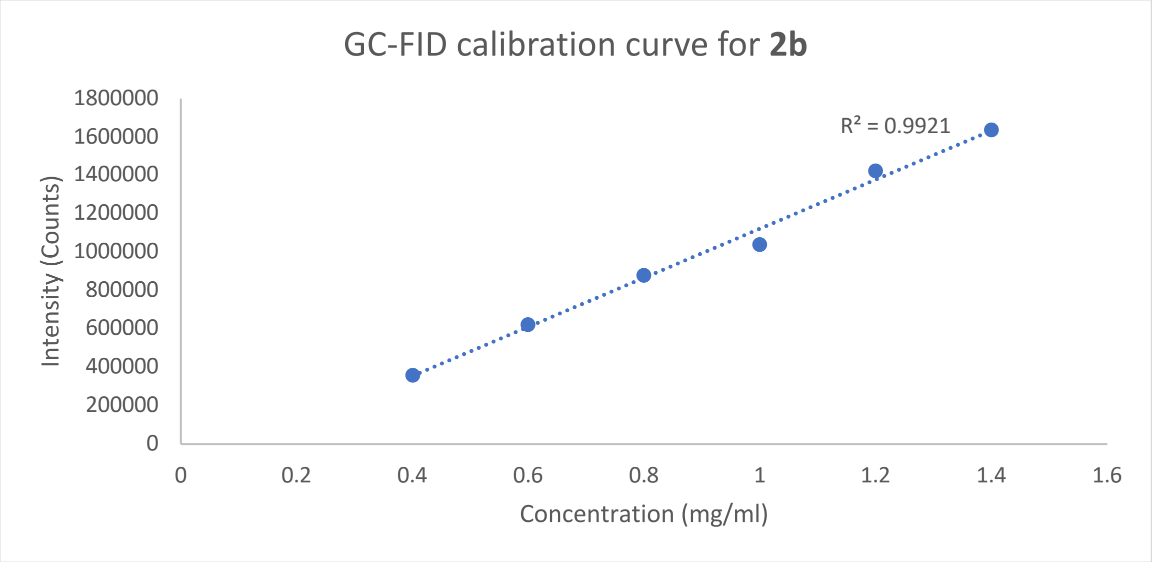

5. Solvent screening table S1:

Performed with general 0.5 mmol protocol with substrate **1c** using GC-MS. Reported as conversion percentages.

	KO*t*Bu	TEA
DMF	88 %	92 %
DMSO	82 %	91 %
DMA	86 %	95 %
THF	0 %	0 %
EtOAc	Traces	0 %
ACN	0 %	32 %
Pivalonitrile	31 %	0 %
IPA	0 %	0 %
Toluene	0 %	0 %
Dioxane	0 %	0 %

6. Expanded screening table S2:

Expanded screening table for **2a**.

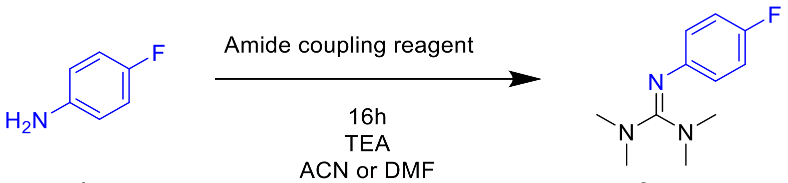

Entry	Notes	Reagent[a]	Solvent	TEA (Equiv.)	Yield of **2a** (%)
1		HATU	ACN	2.0	77 (9)[b]
2		HATU	DMF	2.0	78 (81)[b]
3		HBTU	ACN	2.0	17 (0)[b]
4		HBTU	DMF	2.0	59 (18)[b]
5		HCTU	ACN	2.0	68 (0)[b]
6		HCTU	DMF	2.0	83 (62)[b]
7		HATU	ACN	0.2	70
8		HATU	ACN	1.0	71
9		HATU	ACN	3.0	70
10	2 eq water	HATU	ACN	2.0	0
11	0.5 eq water	HATU	ACN	2.0	20
12		HATU (1.2)	ACN	2.0	81
13		HATU (1.5)	ACN	2.0	99
14	Under argon	HATU	ACN	2.0	70

7. Expanded screening table S3:

Expanded screening table for **2b**.

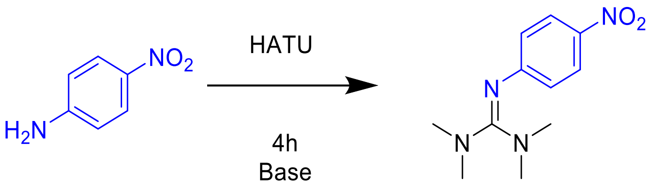

1b **2b**

Entry	Notes	HATU (equiv)	Solvent	Base	Yield of **2b** (%)
1		1	ACN	TEA (2.0)	0
2		1	DMF	TEA (2.0)	0
3		1	DMF	No base	0
4		1	DMF	KO*t*Bu (1.0)	9
5		1	DMF	KO*t*Bu (2.0)	63
6		1	ACN	KO*t*Bu (2.0)	40[c]
7		1.5	DMF	KO*t*Bu (2.0)	45
8		1.5	DMF	KO*t*Bu (3.0)	70
9		HBTU 1 eq	DMF	KO*t*Bu (2.0)	40
10	18-crown-6 (2 eq) added along HATU to the reaction vial	1	DMF	KO*t*Bu (2.0)	28
11	18-crown-6 (2 eq) added by dissolving it and KO*t*Bu in DMF prior to addition	1	DMF	KO*t*Bu (2.0)	39
12		1	DMF	KO*t*Bu + TEA (1 eq both)	30
13		1	DMF	KOH	8
14		1	DMF	DMAP	0
15		1	DMF	DIPEA	0
16		1	DMF	DBU	3
17		1	DMF	DABCO	0
18		1	DMF	PMP	0
19	Under argon	1	DMF	KHMDS	0
20	NaH in mineral oil (60%)	1	DMF	NaH	68

8. Hammett competition protocol:

Into an 8 ml vial was weighed HATU (0.5 mmol) and the vial was loaded with a magnetic stir bar. Solvent (2 ml) was added. When HATU had dissolved, 1 eq of 4-substituted aniline and 1 eq of aniline were added in rapid succession. After loading the competing compounds, base was added (2 eq TEA or 4 eq KO*t*Bu) and the reaction was allowed to stir for 16h with TEA and 1h with KO*t*Bu.

Afterwards, toluene (2.5 ml) and 2M NaOH (2.5 ml) are added and the vial is vigorously shaken. From the organic layer either 1000 µl (if using ACN as solvent) or 500 µl (when using DMF) is transferred to a 1.8 ml GC vial. The solvents are evaporated using an argon flow. The residue is dissolved in DMSO-d_6 and analyzed on Bruker Avance Neo 500.

To acquire the 1H spectra, a relaxation delay of 60 seconds was used with 16 scans. The spectra of isolated products were used as reference for identifying peaks. This gave the conversion of aniline (P_H) and the competing 4-substituted analog (P_X). Values for y-axis were obtained by the following equation:

$$y = \log\left(\frac{P_x}{P_H}\right)$$

Each competition experiment was run in triplicate.

9. Hammett competition figure using σ- values:

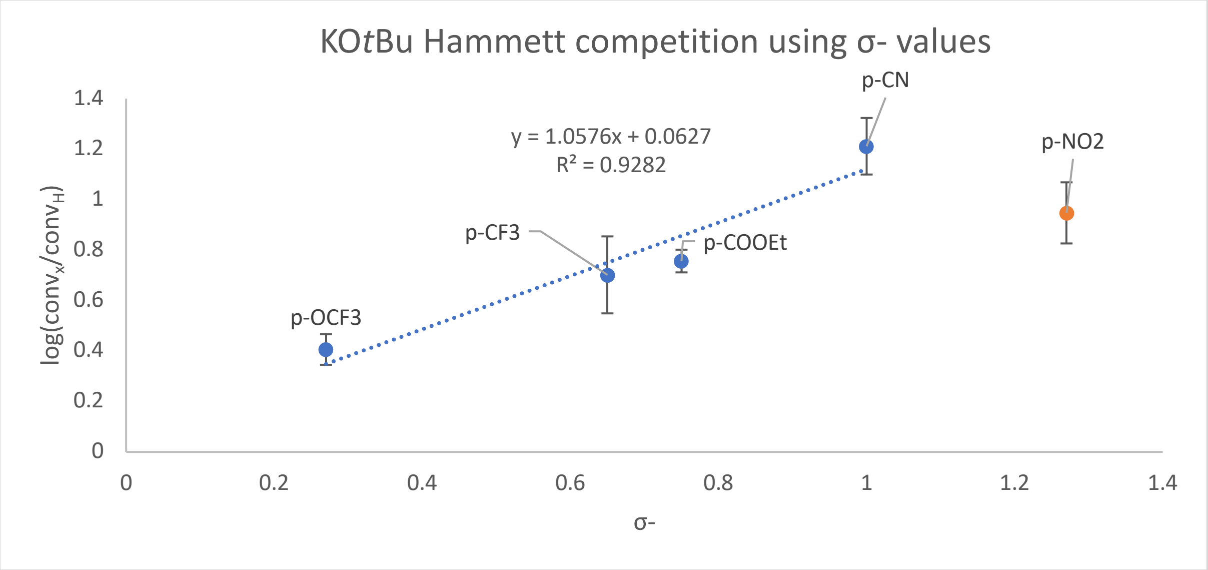

10. Oxalyl chloride-based route test:

All manipulations were performed in anhydrous conditions under argon. Oxalyl chloride (1.1 mL, 1.0 equiv.) was added dropwise via syringe to a stirred solution of tetramethylurea (15.25 mL, 12.7 mmol) in DCM (10 mL). The reaction was refluxed for 2 hours, cooled to room temperature. Then condition 1 or 2 was followed in 1.0 mmol scale.

Condition 1: Nucleophile (1.0 eq, 1.0 mmol) in DCM (2 ml) was slowly added under cooling in an ice bath. After the exothermic reaction had occurred, the mixture was refluxed for 3 h. Solvents were evaporated, and the residue analyzed.

Condition 2: Nucleophile (1.0 eq, 1.0 mmol) and triethylamine (1.0 equiv.) in DCM (2 ml) was slowly added under cooling in an ice bath. After the exothermic reaction had occurred, the mixture was refluxed for 3 h. Solvents were evaporated, and the residue analyzed.

Nucleophile	Condition	Conversion
1b	1	0[a]
1b	2	0[a]
1m	1	100[b]
1m	2	100[b]
1x	1	0[b]
1x	2	0[b]

[a] Conversion determined with GC-FID using mesitylene as an internal standard. [b] Conversion determined in with 19F{1H}-NMR using α,α,α-trifluorotoluene as an internal standard.

11. Product 2u proposed mechanism:

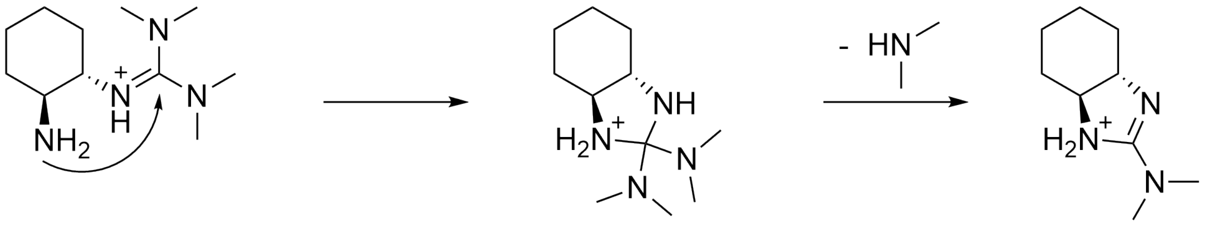

12. Urea 1ad proposed decomposition mechanism:

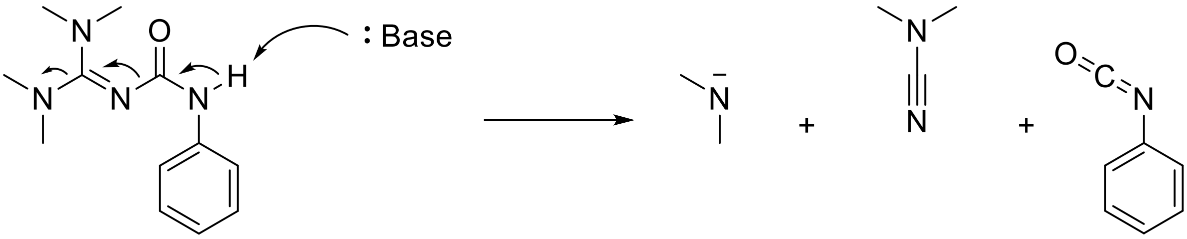

1ad

13. Product nitrogen lone pair isomerization in ^{1}H-NMR:

For electron withdrawing groups such as in compound **2b** the TMG protons are visible as a sharp singlet due to the lone electron pair of the nitrogen being partly delocalized in the aromatic ring. This allows for very fast isomerization of the TMG, resulting in a single TMG peak. However, certain aliphatic amines such as **2m** show a doublet as the TMG peak, indicating a far slower isomerization speed. Electron donating aromatic products such as **2e** are in-between these extremes and therefore show a very broad singlet.

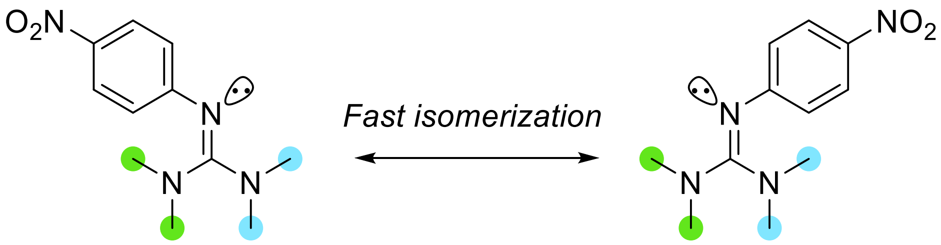

2b **2b**

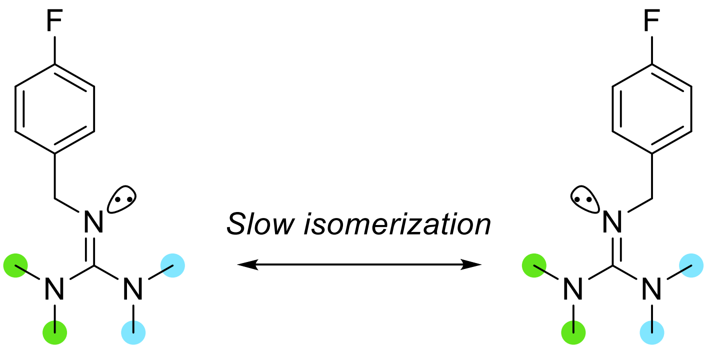

2m **2m**

14. General protocols for 2 mmol scale:

Condition A

HATU (1.2 eq, 2.4 mmol) is added into a 25 ml flask loaded with a magnetic stir bar. If the amine (1.0 eq, 2 mmol) is solid, it is also weighed in. Dry solvent (8 ml of ACN or DMF) is added and the mixture is stirred until everything dissolves. If the amine is liquid, it is added after dissolving the HATU. Then trietylamine (2.4 eq, 4.8 mmol) is added and the mixture is left to stir overnight.

Condition B

HATU (1.2 eq, 2.4 mmol) is added into a 25 ml flask loaded with a magnetic stir bar. If the amine (1.0 eq, 2 mmol) is solid, it is also weighed in. Dry DMF (8 ml) is added and the mixture is stirred until everything dissolves. If the amine is liquid, it is added after dissolving the HATU. Then KO*t*Bu (2.4 eq) is added and the mixture is allowed to stir for 4 h.

Workup 1: Add the reaction mixture into a separatory funnel. Add 40 ml 2M NaOH solution. Extract the aqueous phase with 3 x 30 ml toluene:EtOAc (3:1). Combine the organic phases and dry with anhydrous Na_2SO_4 and evaporate the solvent. Dry the residue under vacuum.

Workup 2: Add the reaction mixture into a separatory funnel. Add 40 ml 2M NaOH solution. Extract the aqueous phase three times with toluene:EtOAc (3:1). Combine the organic phases and dry with anhydrous Na_2SO_4 and evaporate the solvent. In a separate beaker, dissolve 900 mg oxalic acid in 30 ml diethyl ether. Add the evaporation residue dropwise to the oxalic acid solution while stirring. Once addition is complete, turn off stirring and let the precipitate settle. Decant the diethyl ether and wash the remaining solid with fresh diethyl ether (2 x 30 ml). Dissolve the solid in 40 ml 2M NaOH and transfer the solution to a separatory funnel. extracted with 3 x 30 ml toluene:EtOAc (3:1). The organics are combined and dried with anhydrous Na_2SO_4 and the solvent is evaporated to yield the product.

Workup 3: Add the reaction mixture into a separatory funnel. Add 40 ml 2M NaOH solution. Extract the aqueous phase three times with 3 x 30 ml toluene:EtOAc (3:1). Combine the organic phases and dry with anhydrous Na_2SO_4 and evaporate the solvent. Purify the residue by silica gel column chromatography using 90:10 EtOAc:MeOH as solvent.

15. Procedures and Analytical Data:

2a

1,1,3,3-Tetramethyl-2-(4-fluorophenyl)guanidine

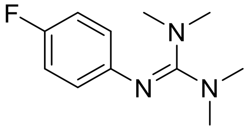

Condition A with ACN. Workup 2. Light brown oil. Yield 380 mg (90 %).

1H NMR (400 MHz, $CDCl_3$) δ [ppm]: 6.93-6.85 (m, 2H), 6.66-6.59 (m, 2H), 2.69 (bs, 12H).

13C{1H} NMR (101 MHz, CDCl3) δ [ppm]: 160.1, 157.4 (d, J = 237.2), 147.9 (d, J = 2.4 Hz), 122.4 (d, J = 7.6), 115.2 (d, J = 21.9 Hz), 39.6 (bs).

19F{1H} NMR (377 MHz, CDCl3) δ [ppm]: -125.3

HRMS (ESI-TOF) m/z: [M+H]+ Calcd for C11H17FN3 210.1401; found 210.1401

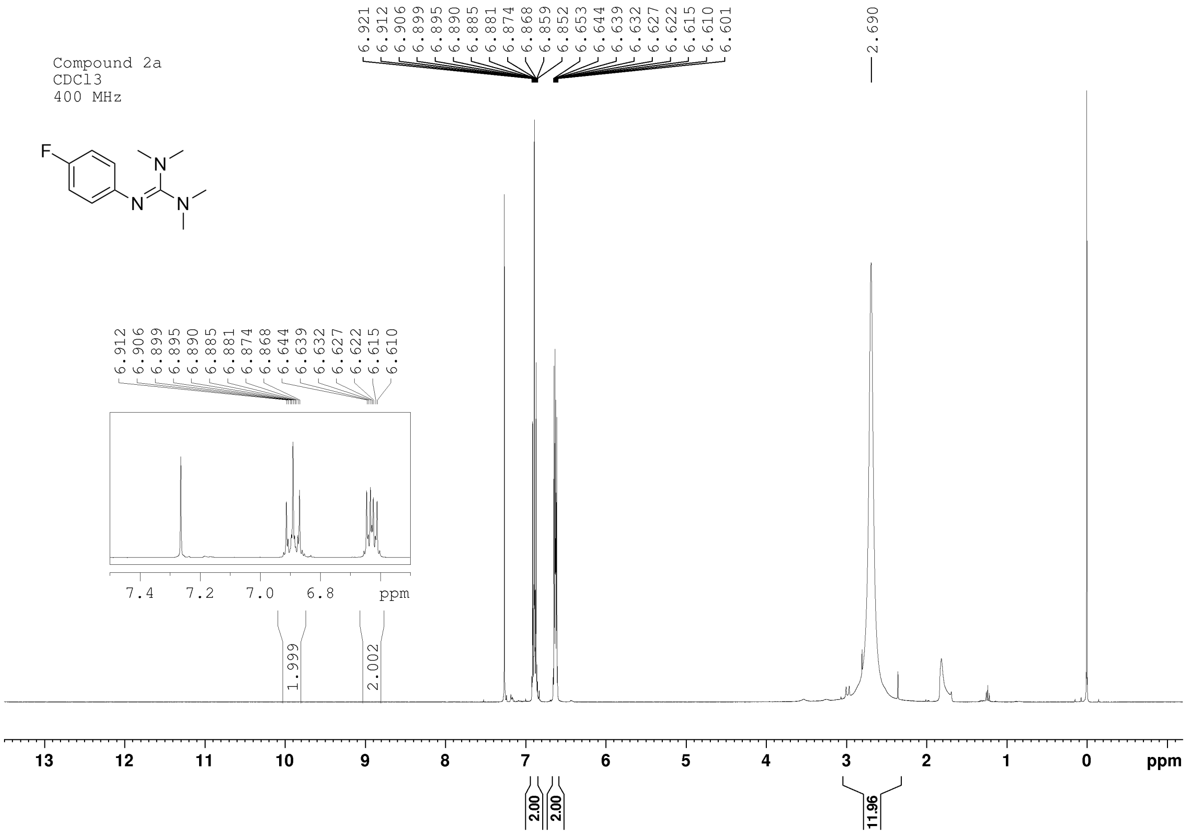

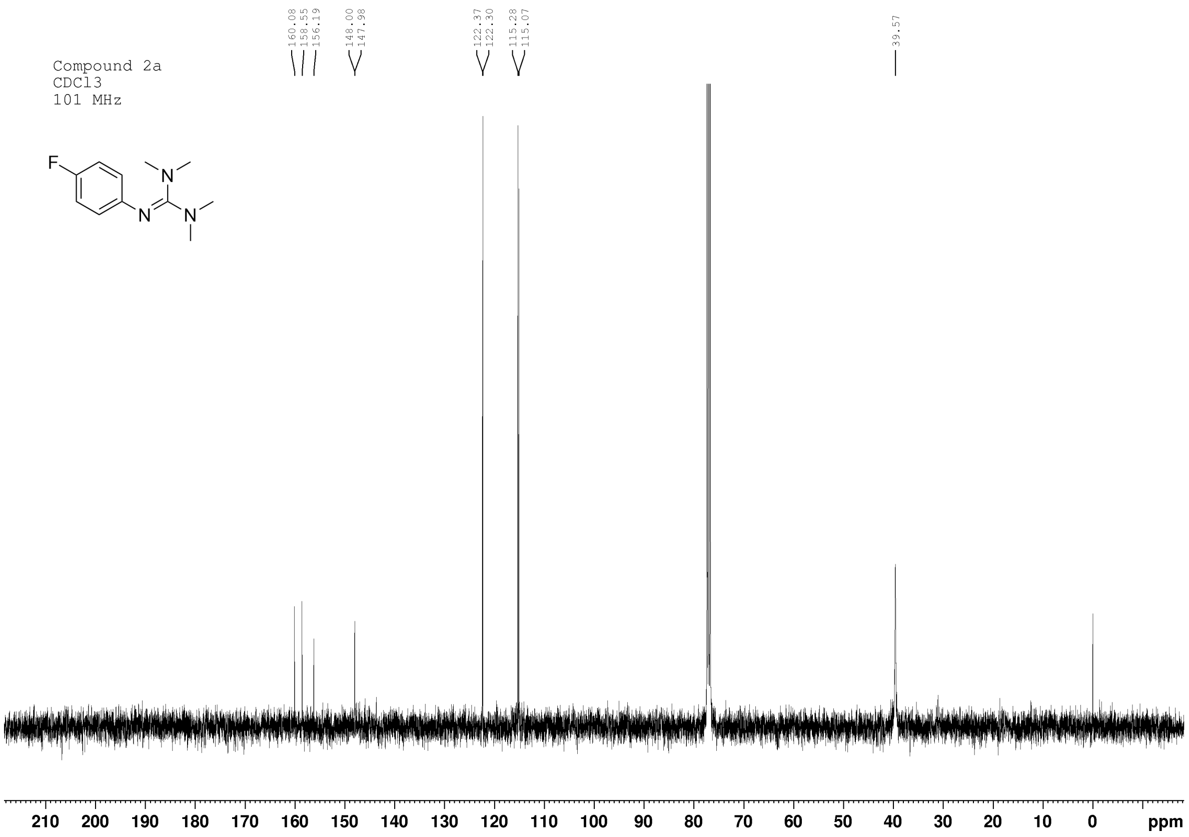

Compound 2a
CDCl3
101 MHz
160.08
158.55
156.19
148.00
147.98
122.37
122.30
115.28
115.07
39.57
F
N
N
N
210 200 190 180 170 160 150 140 130 120 110 100 90 80 70 60 50 40 30 20 10 0 ppm

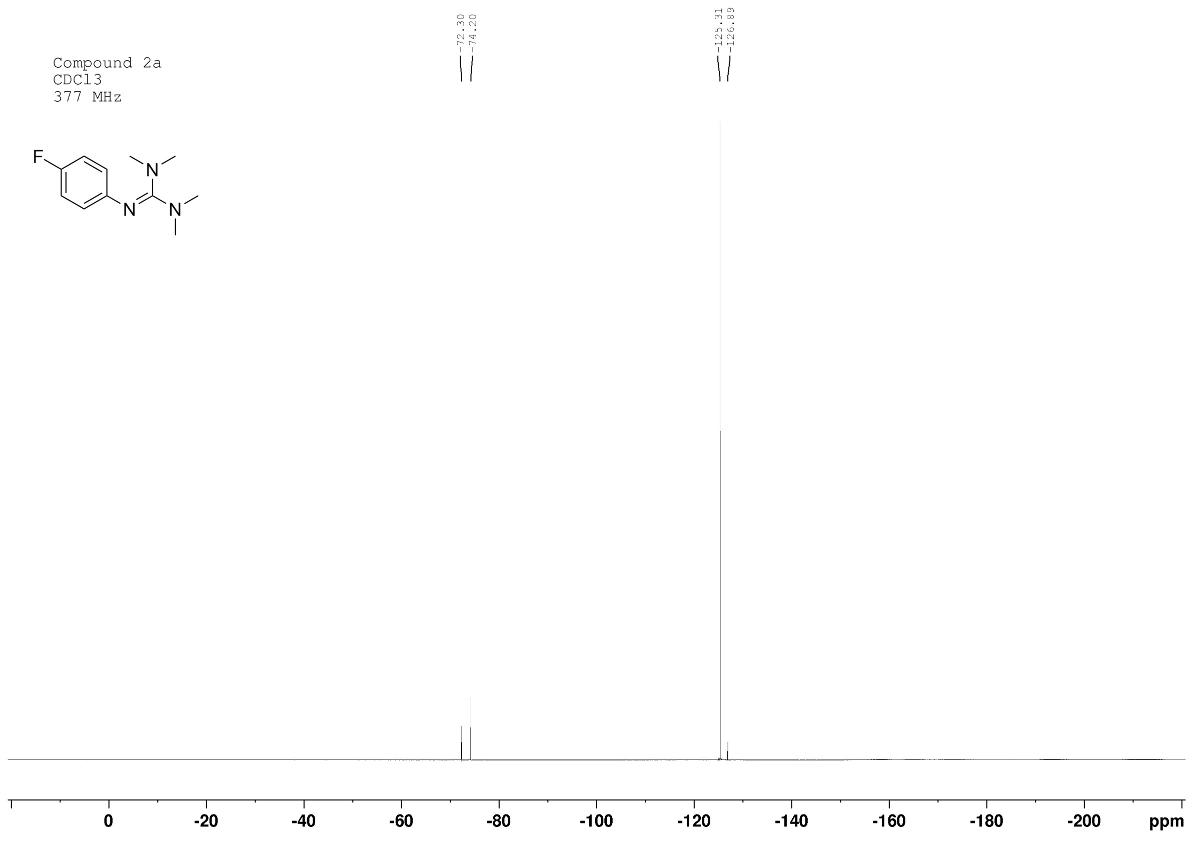

Compound 2a
CDCl3
377 MHz
-72.30
-74.20
-125.31
-126.89
F
N
N
N
0 -20 -40 -60 -80 -100 -120 -140 -160 -180 -200 ppm

2b

1,1,3,3-Tetramethyl-2-(4-nitrophenyl)guanidine

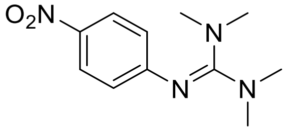

Condition B. Workup 2. Bright orange solid. Yield 430 mg (91 %)

1H NMR (400 MHz, $CDCl_3$) δ [ppm]: 8.15-8.01 (m, 2H), 6.70-6.59 (m, 2H), 2.77 (s, 12H)

13C{1H} NMR (101 MHz, CDCl3) δ [ppm]: 161.4, 159.5, 139.9, 125.4, 120.8, 39.8

HRMS (ESI-TOF) m/z: [M+H]+ Calcd for C11H17N4O2 237.1346; found 237.1348

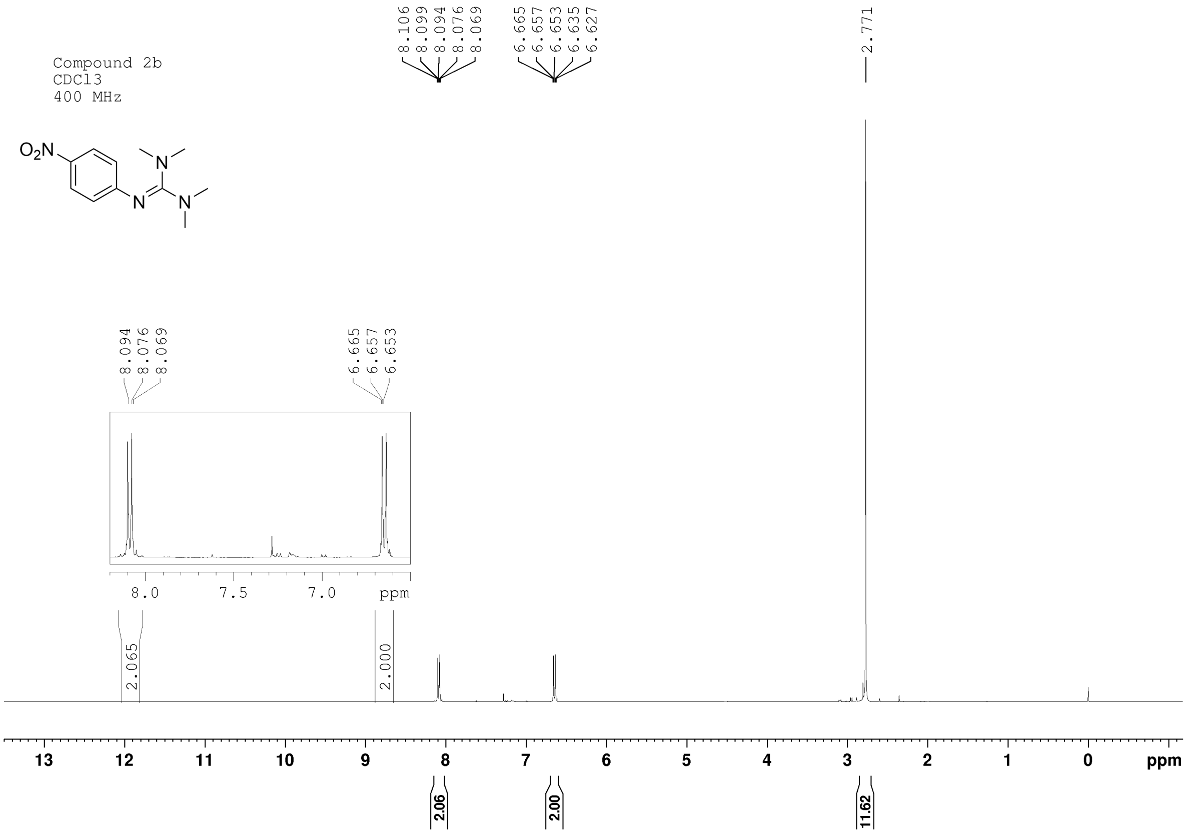

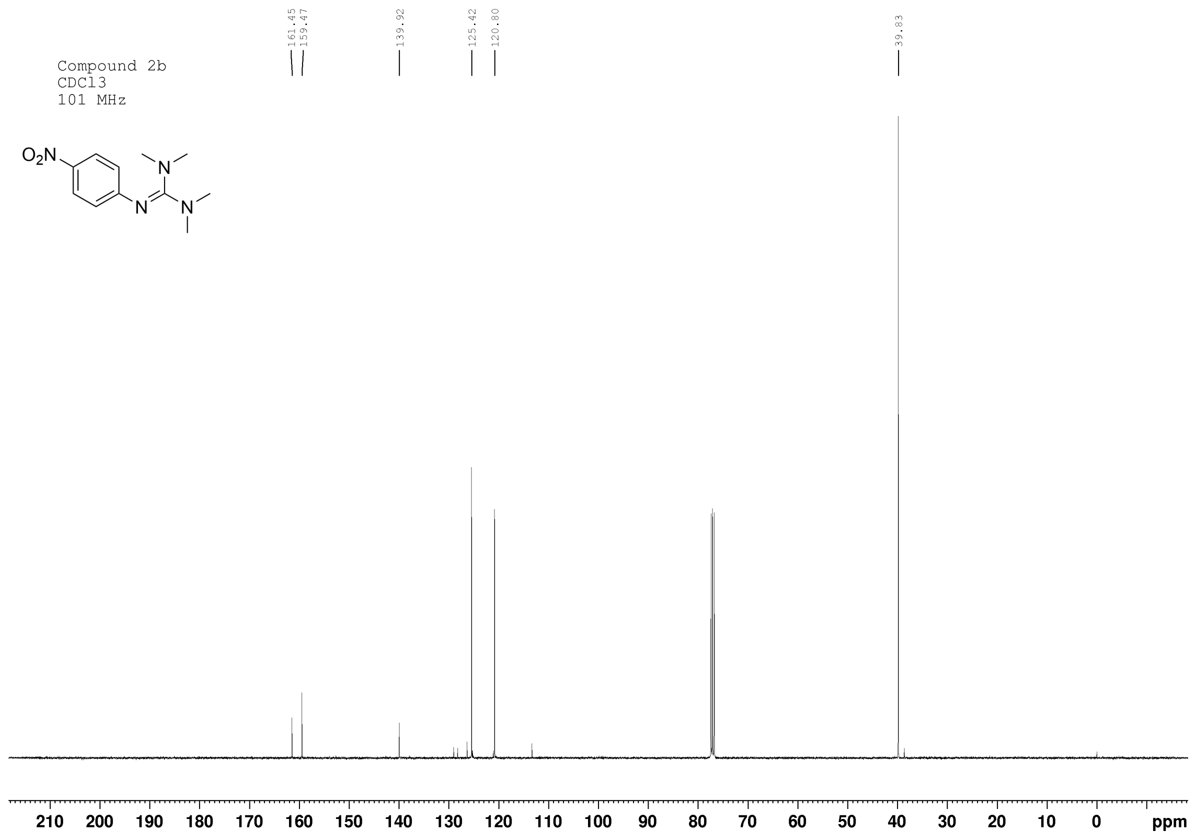

2c

1,1,3,3-Tetramethyl-2-phenylguanidine

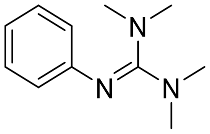

Condition A with ACN. Workup 2. Light brown oil. Yield 341 mg (89 %).

1H NMR (400 MHz, $CDCl_3$) δ [ppm]: 7.22-7.16 (m, 2H), 6.86-6.80 (tt, 1H, J = 7.3, 1), 6.73-6.67 (m, 2H), 2.70 (bs, 12H).

13C{1H} NMR (101 MHz, CDCl3) δ [ppm]: 159.9, 151.9, 128.7, 121,7, 119.8, 39.6 (bs)

HRMS (ESI-TOF) m/z: [M+H]+ Calcd for C11H18N3 192.1495; found 192.1499

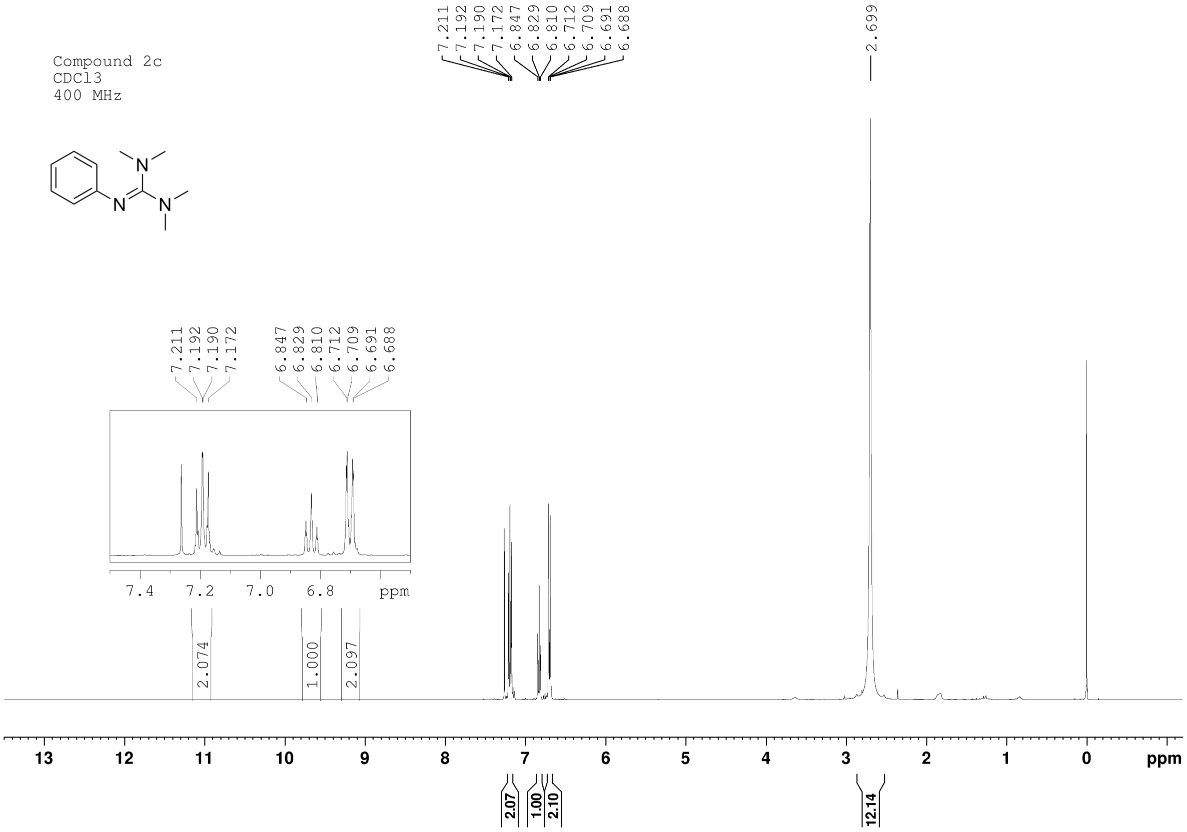
Compound 2c
CDCl3
400 MHz
N
N
N
7.211
7.192
7.190
7.172
6.847
6.829
6.810
6.712
6.709
6.691
6.688
2.699
7.4 7.2 7.0 6.8 ppm
2.074
1.000
2.097
13 12 11 10 9 8 7 6 5 4 3 2 1 0 ppm
2.07
1.00
2.10
12.14

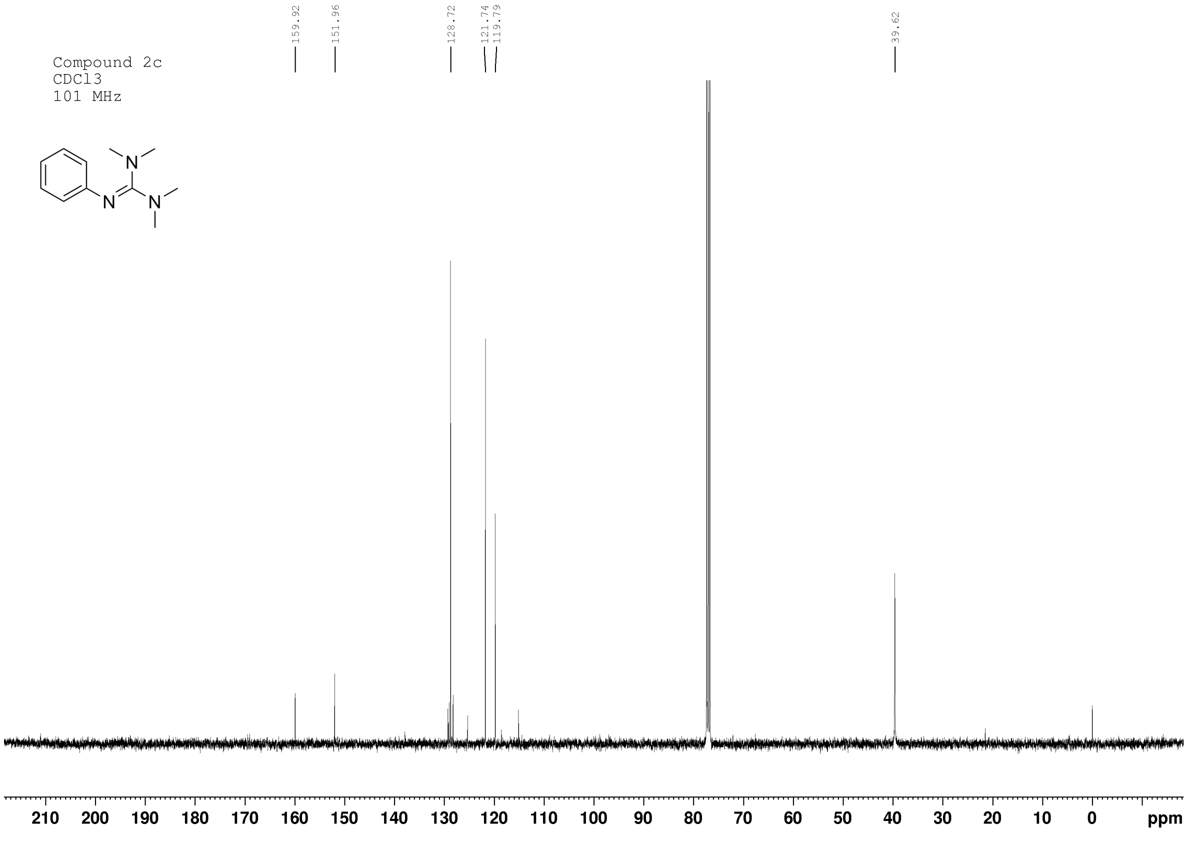
Compound 2c
CDCl3
101 MHz
N
N
N
159.92
151.96
128.72
121.74
119.79
39.62
210 200 190 180 170 160 150 140 130 120 110 100 90 80 70 60 50 40 30 20 10 0 ppm

2d

1,1,3,3-Tetramethyl-2-(p-tolyl)guanidine

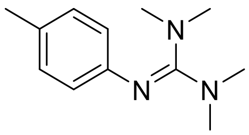

Condition A with ACN. Workup 1. Dark brown oil. Yield 362 mg (88 %).

1H NMR (400 MHz, $CDCl_3$) δ [ppm]: 7.00 (d, J= 8.0, 2H), 6.60 (d, J = 8.2, 2H), 2.69 (bs, 12H), 2.26 (s, 3H).

13C{1H} NMR (101 MHz, CDCl3) δ [ppm]: 159.7, 149.2, 129.3, 128.9, 121.5, 39.6 (bs), 20.7

HRMS (ESI-TOF) m/z: [M+H]+ Calcd for $C_{12}H_{20}N_3$ 206.1652; found 206.1655

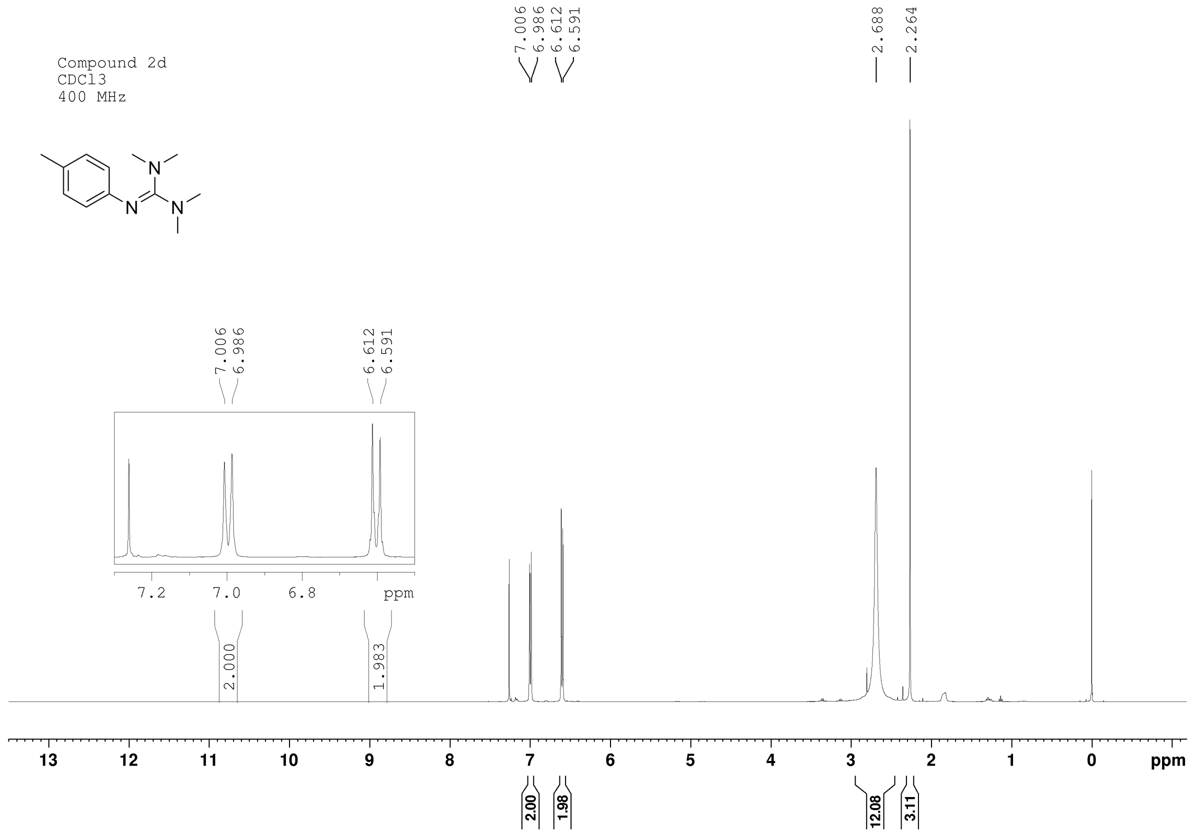

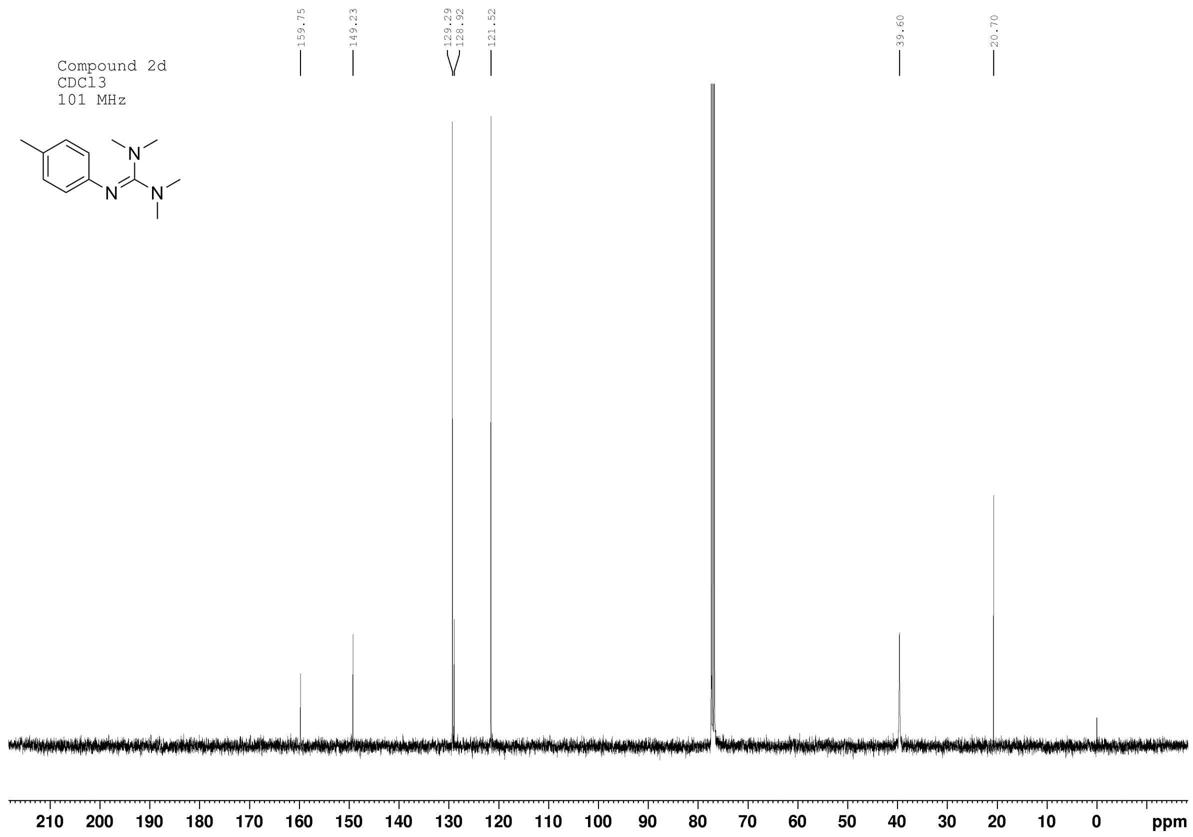

2e

1,1,3,3-Tetramethyl-2-(4-methoxyphenyl)guanidine

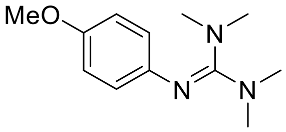

Condition A with ACN. Workup 1. Red-brown oil. Yield 433 mg (98 %).

1H NMR (400 MHz, $CDCl_3$) δ [ppm]: 6.81-6.75 (m, 2H), 6.67-6.61 (m, 2H), 3.76 (s, 3H), 2.68 (bs, 12H).

13C{1H} NMR (101 MHz, CDCl3) δ [ppm]: 159.8, 153.5, 145.4, 122.3, 114.2, 55.6, 39.5 (bs)

HRMS (ESI-TOF) m/z: [M+H]+ Calcd for $C_{12}H_{20}N_3O$ 222.1601; found 222.1597

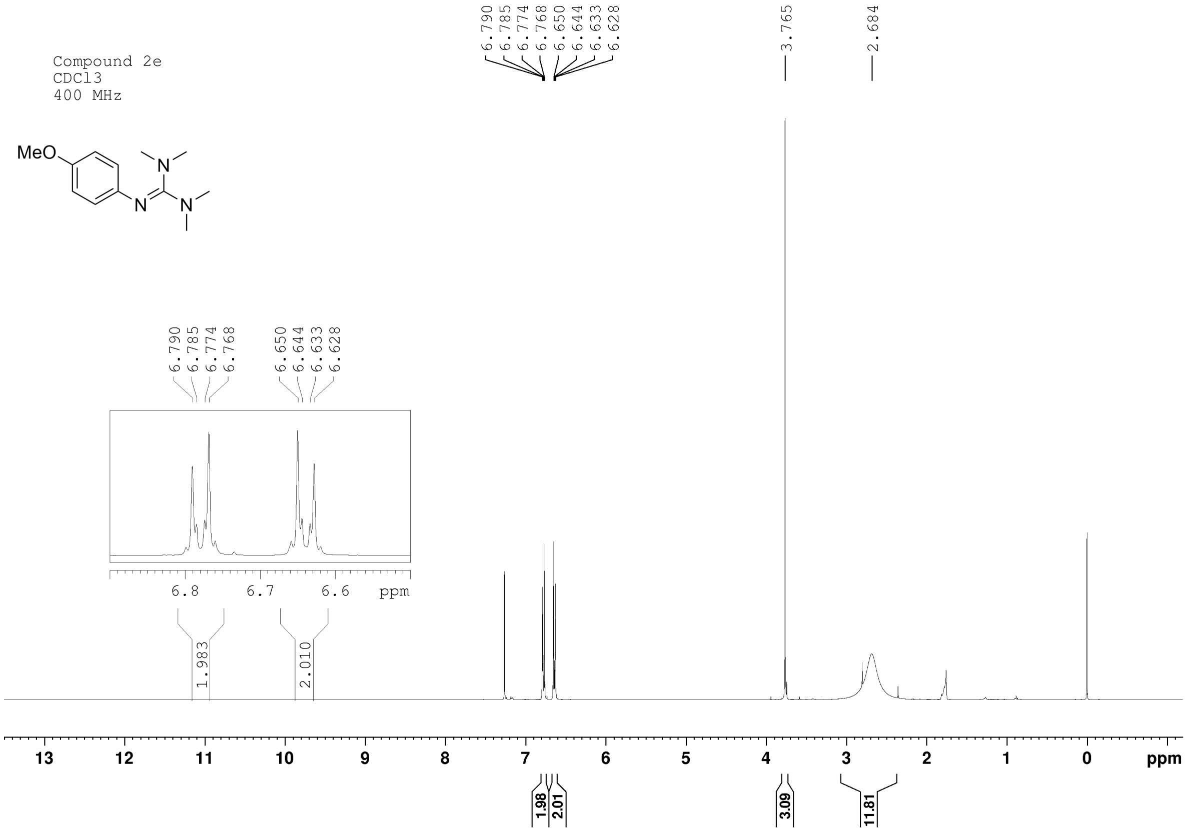
6.790
6.785
6.774
6.768
6.650
6.644
6.633
6.628
3.765
2.684
Compound 2e
CDCl3
400 MHz
MeO
N
N
N
6.8
6.7
6.6
ppm
1.983
2.010
13
12
11
10
9
8
7
6
5
4
3
2
1
0
ppm
1.98
2.01
3.09
11.81

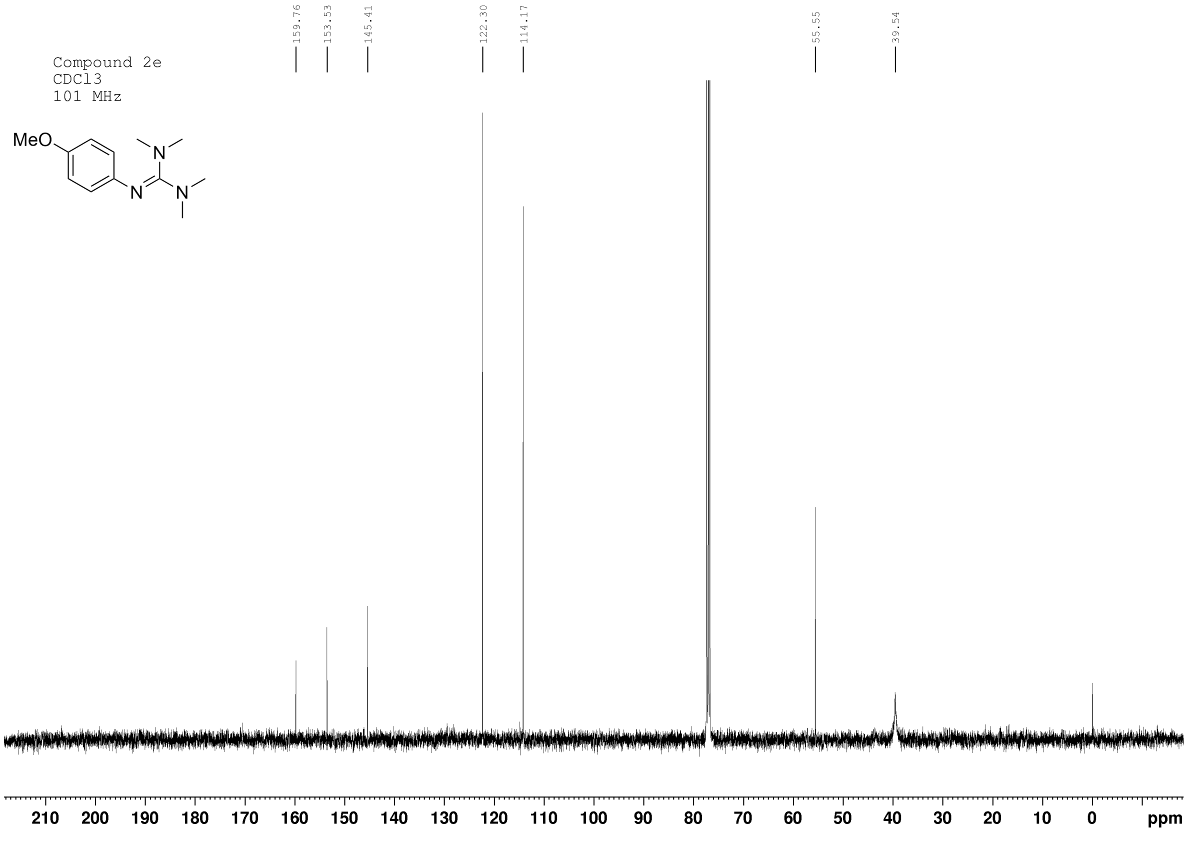
159.76
153.53
145.41
122.30
114.17
55.55
39.54
Compound 2e
CDCl3
101 MHz
MeO
N
N
N
210
200
190
180
170
160
150
140
130
120
110
100
90
80
70
60
50
40
30
20
10
0
ppm

2f

1,1,3,3-Tetramethyl-2-(4-(trifluoromethyl)phenyl)guanidine

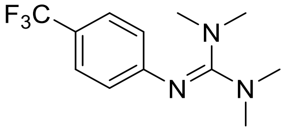

Condition B. Workup 2. Brown oil. Yield 299 mg (58 %).

1H NMR (400 MHz, $CDCl_3$) δ [ppm]: 7.43 (d, J = 8.3, 2H), 6.72 (d, J = 8.2, 2H), 2.72 (s, 12H).

13C{1H} NMR (101 MHz, CDCl3) δ [ppm]: 160.6, 155.4, 126.0 (q, J = 3.8), 121.3, 121.3 (q, J = 32.4), 39.7

19F{1H} NMR (377 MHz, CDCl3) δ [ppm]: -61.2

HRMS (ESI-TOF) m/z: [M+H]+ Calcd for C12H17F3N3 260.1369; found 260.1369

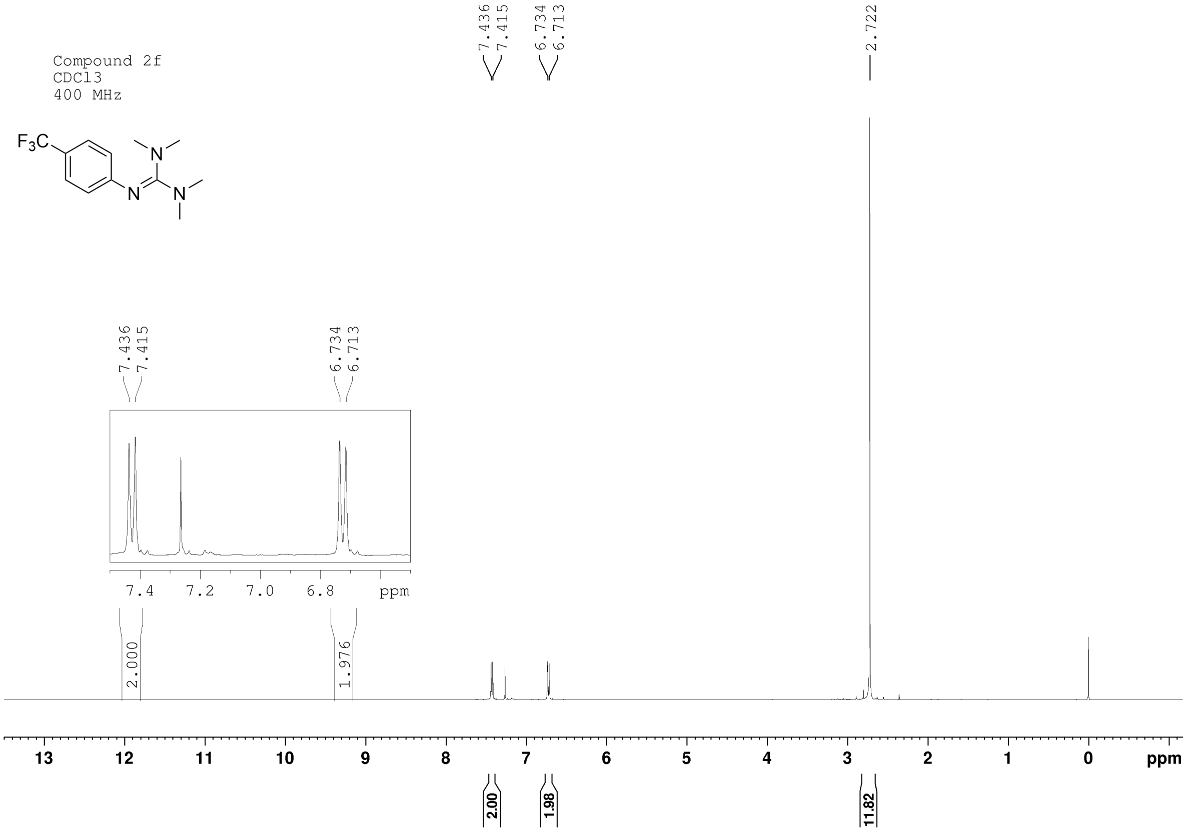

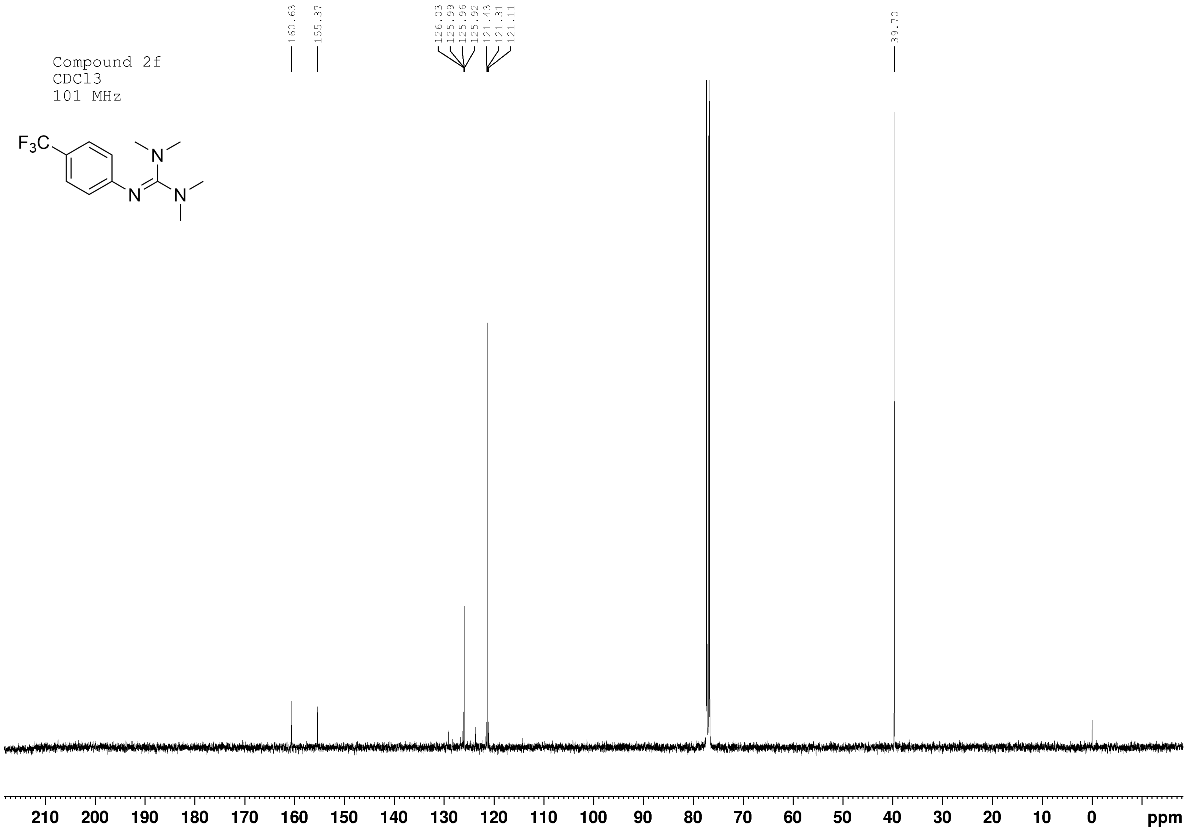
Compound 2f
CDCl3
101 MHz
F_3C
N
N
N
160.63
155.37
126.03
125.99
125.96
125.92
121.43
121.31
121.11
39.70
210 200 190 180 170 160 150 140 130 120 110 100 90 80 70 60 50 40 30 20 10 0 ppm

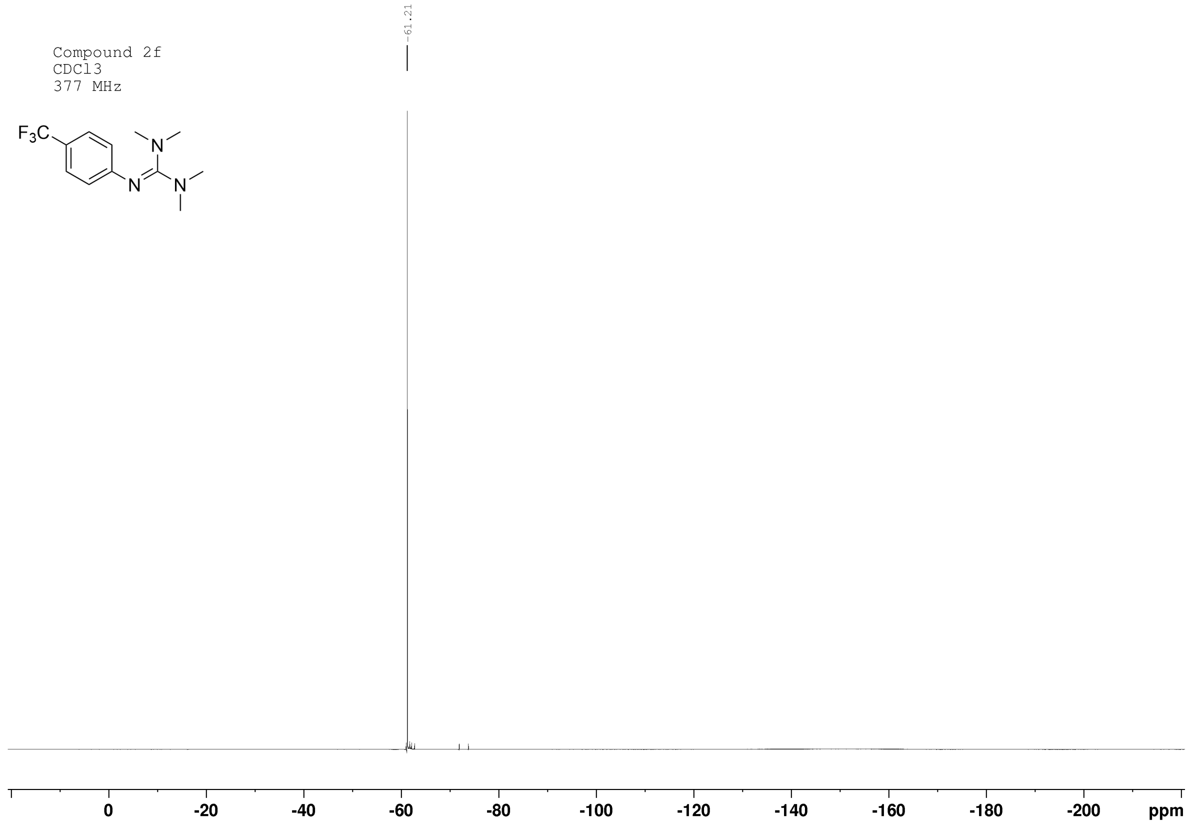
Compound 2f
CDCl3
377 MHz
F_3C
N
N
N
-61.21
0 -20 -40 -60 -80 -100 -120 -140 -160 -180 -200 ppm

2g

1,1,3,3-Tetramethyl-2-(3,5-bis(trifluoromethyl)phenyl)guanidine

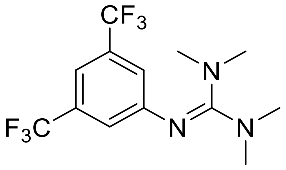

Condition B. Workup 2. Brown solid. Yield 486 mg (74 %)

1H NMR (400 MHz, $CDCl_3$) δ [ppm]: 7.28 (bs, 1H), 7.06 (bs, 2H), 2.74 (bs, 12H).

13C{1H} NMR (101 MHz, CDCl3) δ [ppm]: 161.2, 153.4, 131.8 (q, J = 32.4), 123.7 (q, J = 272.5), 121.1 (d, J = 1.5), 112.5 (p, J = 4.0), 39.7 (bs).

19F{1H} NMR (377 MHz, CDCl3) δ [ppm]: -63.0

HRMS (ESI-TOF) m/z: [M+H]+ Calcd for C13H16F6N3 328.1243; found 328.1248

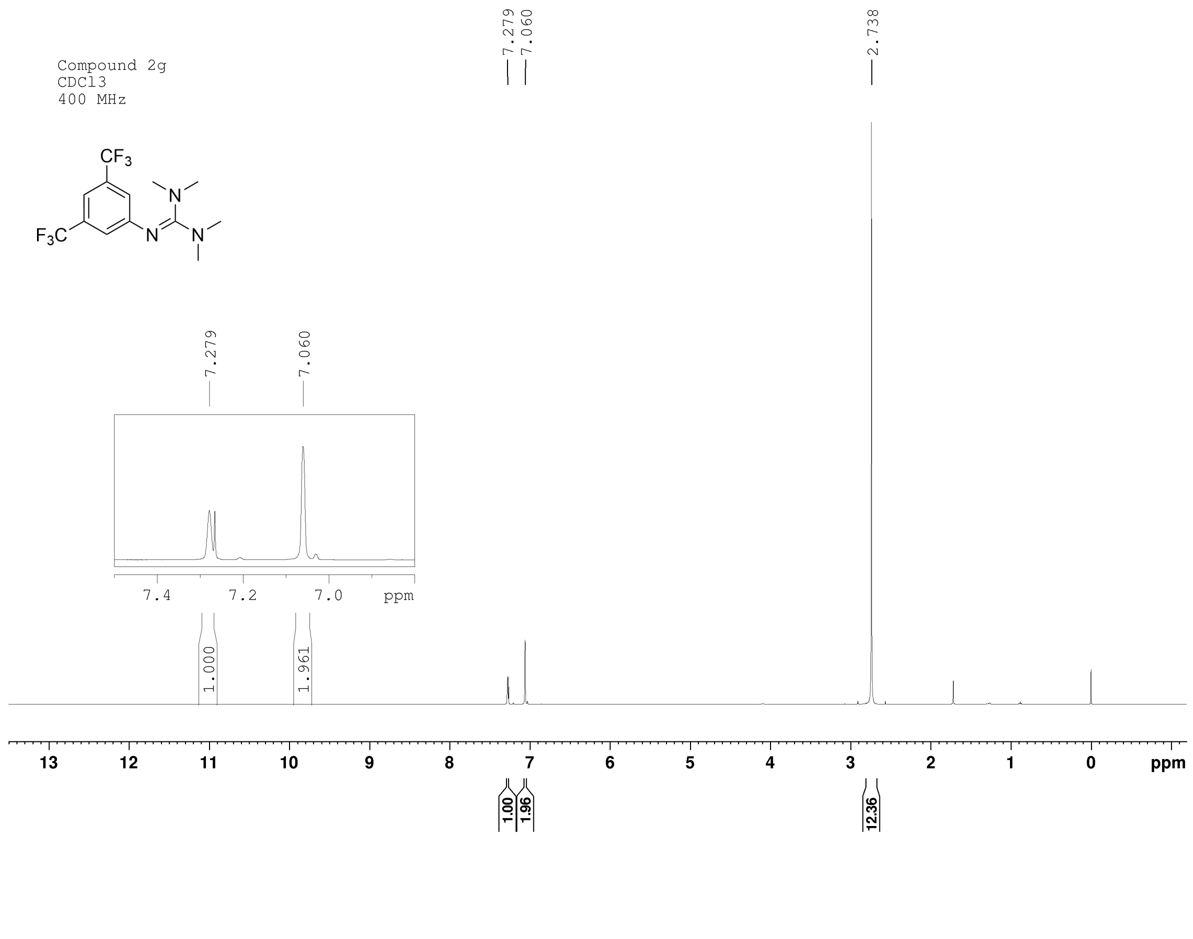

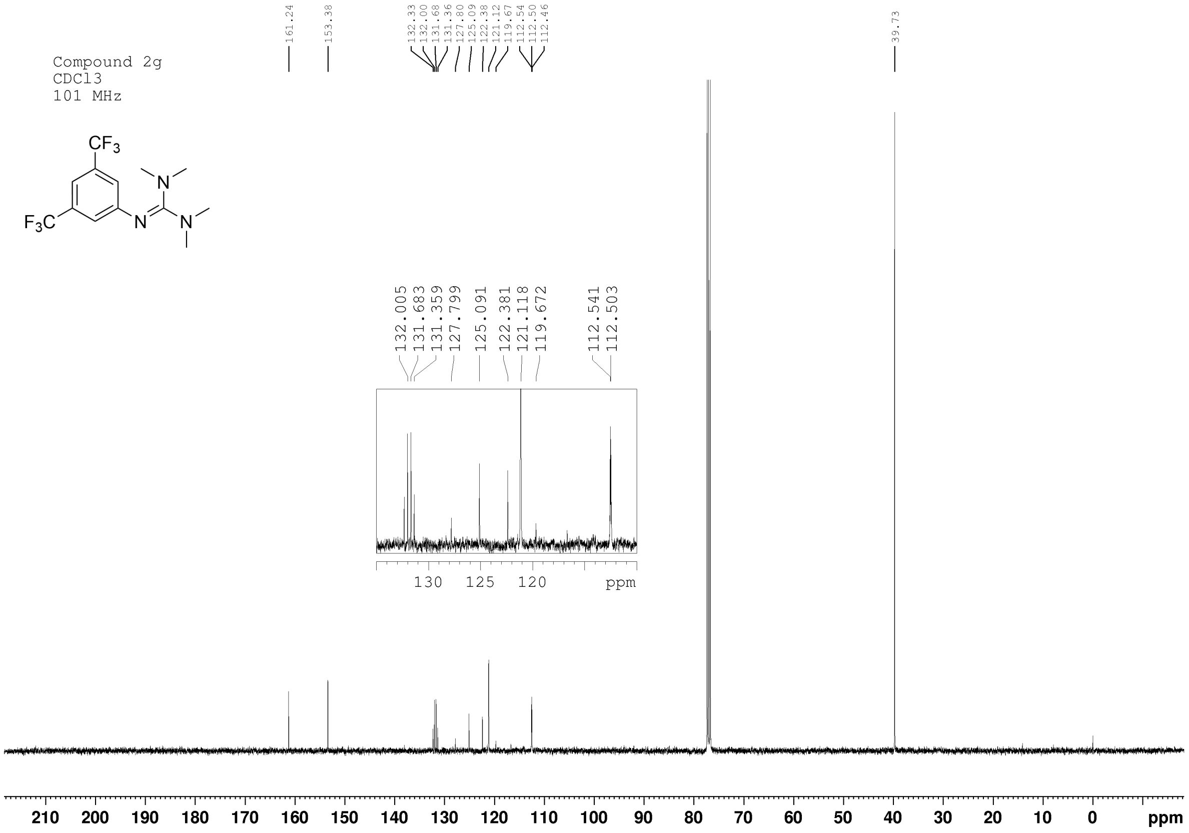
Compound 2g
CDCl3
101 MHz
CF3
F3C
N
161.24
153.38
132.33
132.00
131.68
131.36
127.80
125.09
122.38
121.12
119.67
112.54
112.50
112.46
39.73
132.005
131.683
131.359
127.799
125.091
122.381
121.118
119.672
112.541
112.503
ppm

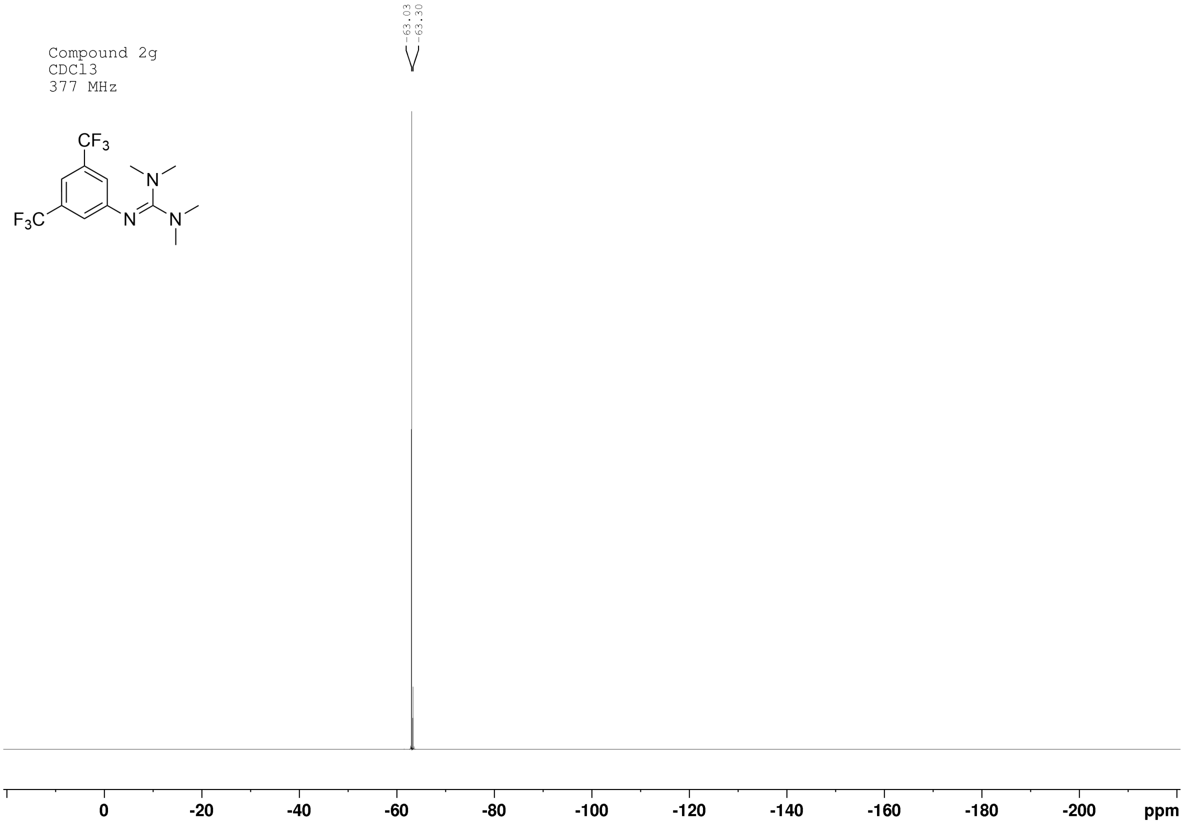
Compound 2g
CDCl3
377 MHz
CF3
F3C
N
-63.03
-63.30
ppm

2h

1,1,3,3-Tetramethyl-2-(4-(pentafluoro-λ6-sulfaneyl)phenyl)guanidine

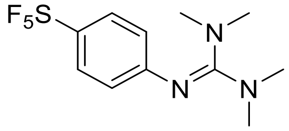

Condition B. Workup 2. Orange oil. Yield 320 mg (51 %).

1H NMR (400 MHz, $CDCl_3$) δ [ppm]: 7.55 (d, J = 9.0, 2H), 6.65 (d, J = 8.74, 2H), 2.73 (s, 12H).

13C{1H} NMR (101 MHz, CDCl3) δ [ppm]: 160.9, 155.1, 145.7, 126.7 (m), 120.6, 39.7

19F{1H} NMR (377 MHz, CDCl3) δ [ppm]: 87.9 (quint, J = 149.9), 64.7, 64.3

HRMS (ESI-TOF) m/z: [M+H]+ Calcd for C11H17F5N3S 318.1058; found 318.1069

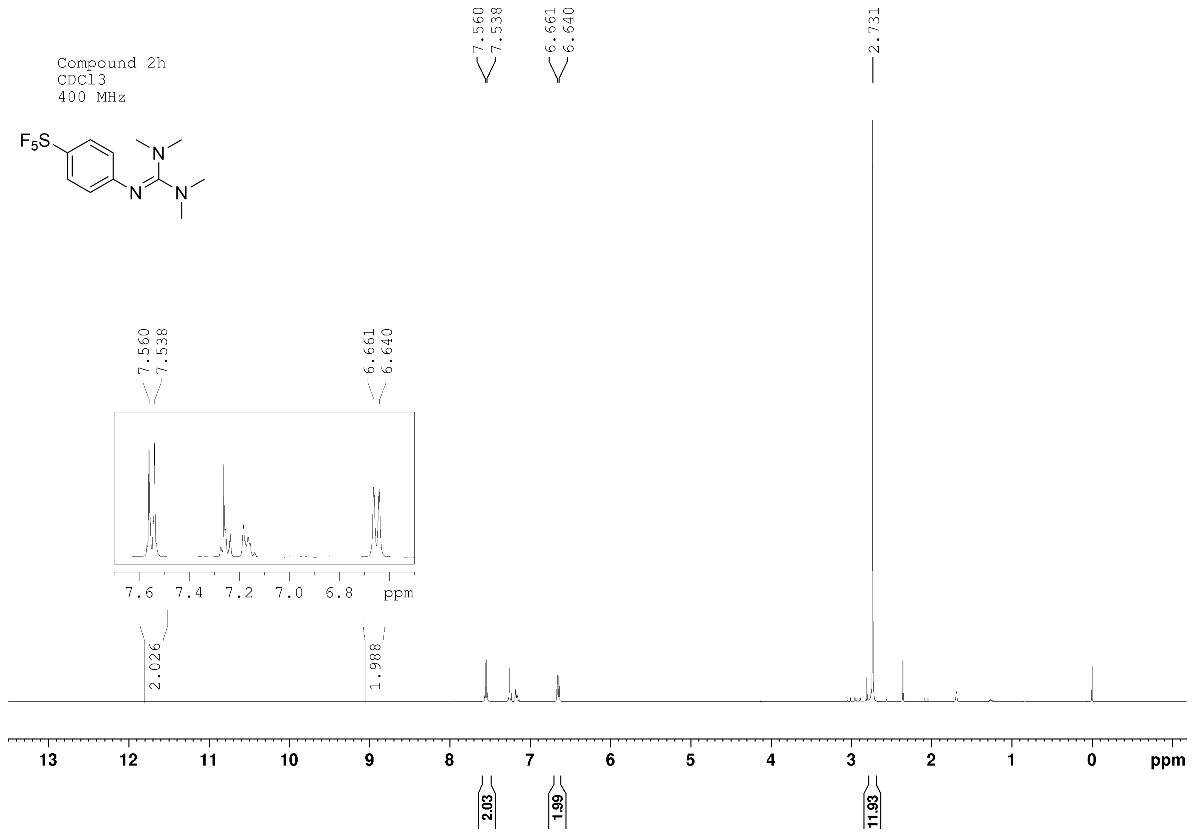

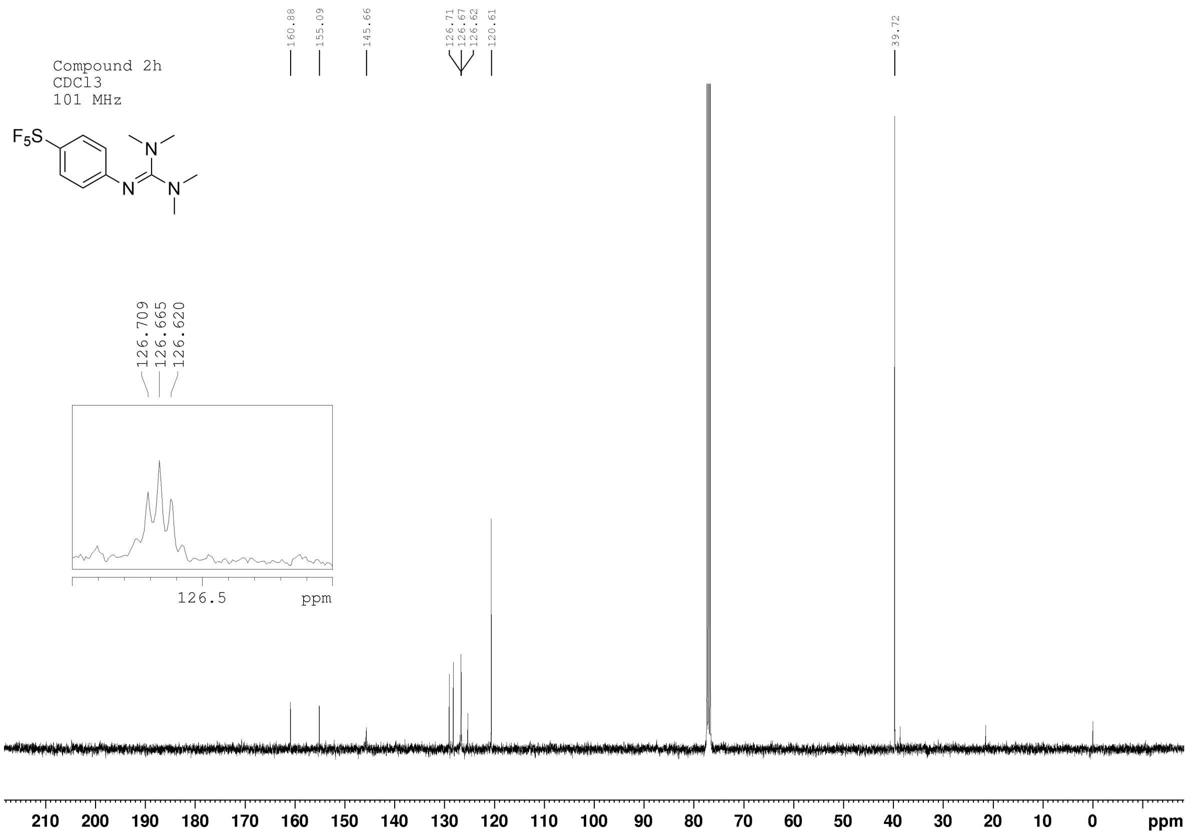
Compound 2h
CDCl3
101 MHz
F5S
N
N
N
160.88
155.09
145.66
126.71
126.67
126.62
120.61
39.72
126.709
126.665
126.620
126.5
ppm
210 200 190 180 170 160 150 140 130 120 110 100 90 80 70 60 50 40 30 20 10 0
ppm

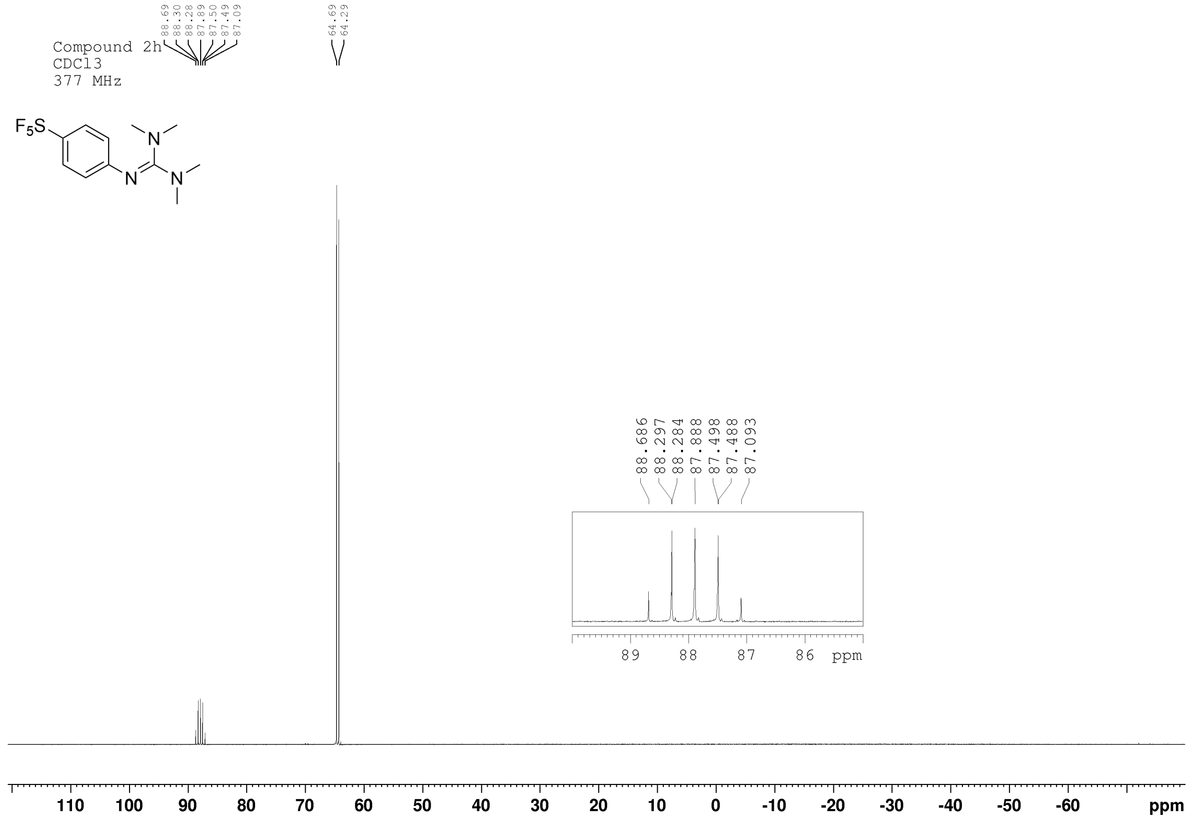
Compound 2h
CDCl3
377 MHz
F5S
N
N
N
88.69
88.30
88.28
87.89
87.50
87.49
87.09
64.69
64.29
88.686
88.297
88.284
87.888
87.498
87.488
87.093
89 88 87 86 ppm
110 100 90 80 70 60 50 40 30 20 10 0 -10 -20 -30 -40 -50 -60
ppm

2i

1,1,3,3-Tetramethyl-2-(perfluorophenyl)guanidine

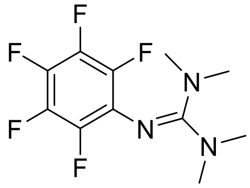

Condition B. Workup 2. Purple solid. Yield 340 mg (61 %).

1H NMR (400 MHz, $CDCl_3$) δ [ppm]: 2.77 (s, 12H).

13C{1H} NMR (101 MHz, CDCl3) δ [ppm]: 163.3, 141.4 (m), 139.1 (m), 136.8 (m), 135.5 (m), 133.1 (m), 127.7 (m), 39.2

19F{1H} NMR (377 MHz, CDCl3) δ [ppm]: -156.8, -165.8, -170.5

HRMS (ESI-TOF) m/z: [M+H]+ Calcd for C11H13F5N3 282.1024; found 282.1036

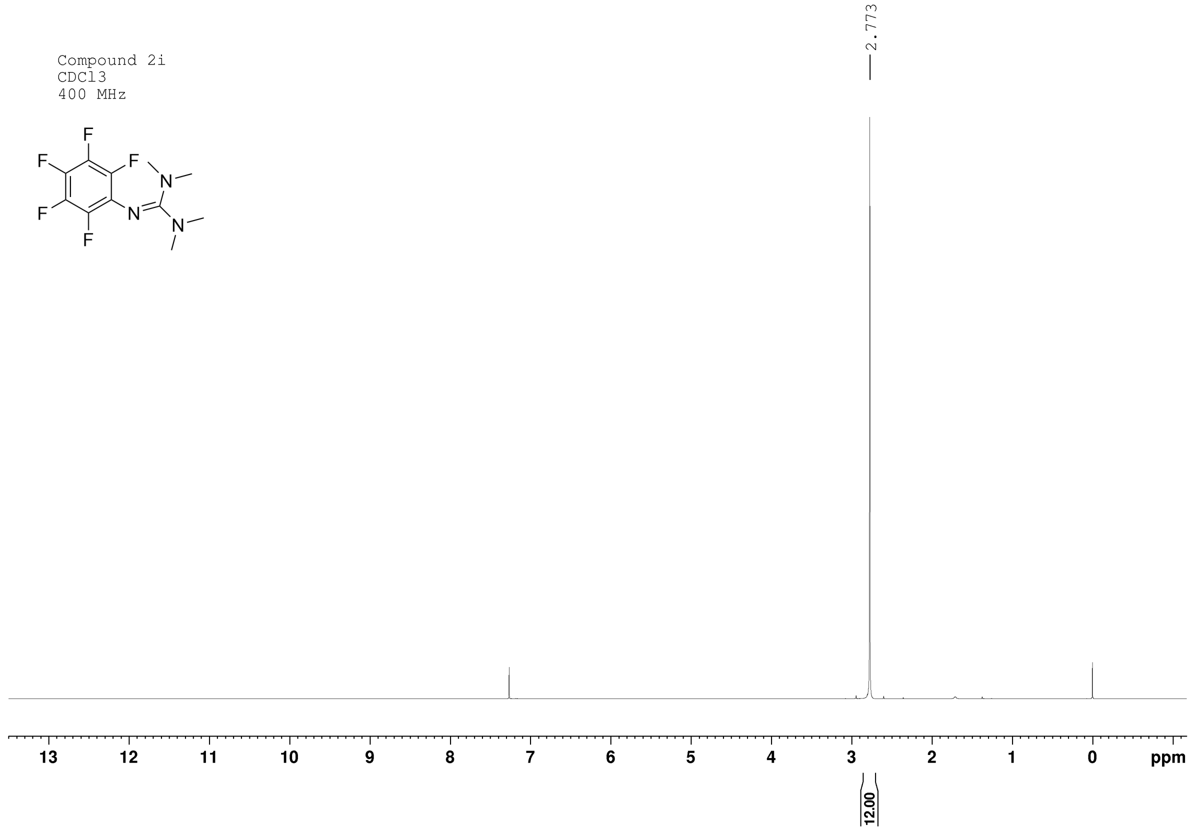

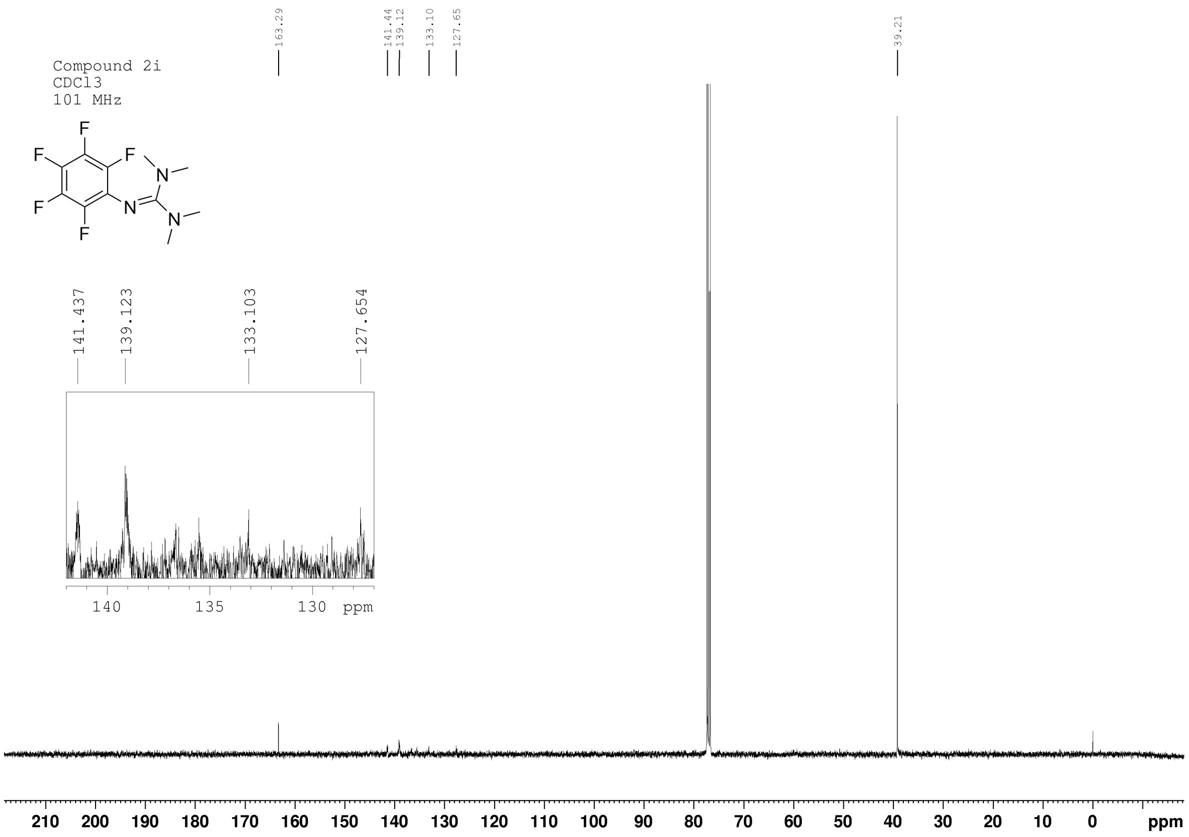
163.29
141.44
139.12
133.10
127.65
39.21
Compound 2i
CDCl3
101 MHz
141.437
139.123
133.103
127.654
140
135
130 ppm
210 200 190 180 170 160 150 140 130 120 110 100 90 80 70 60 50 40 30 20 10 0 ppm

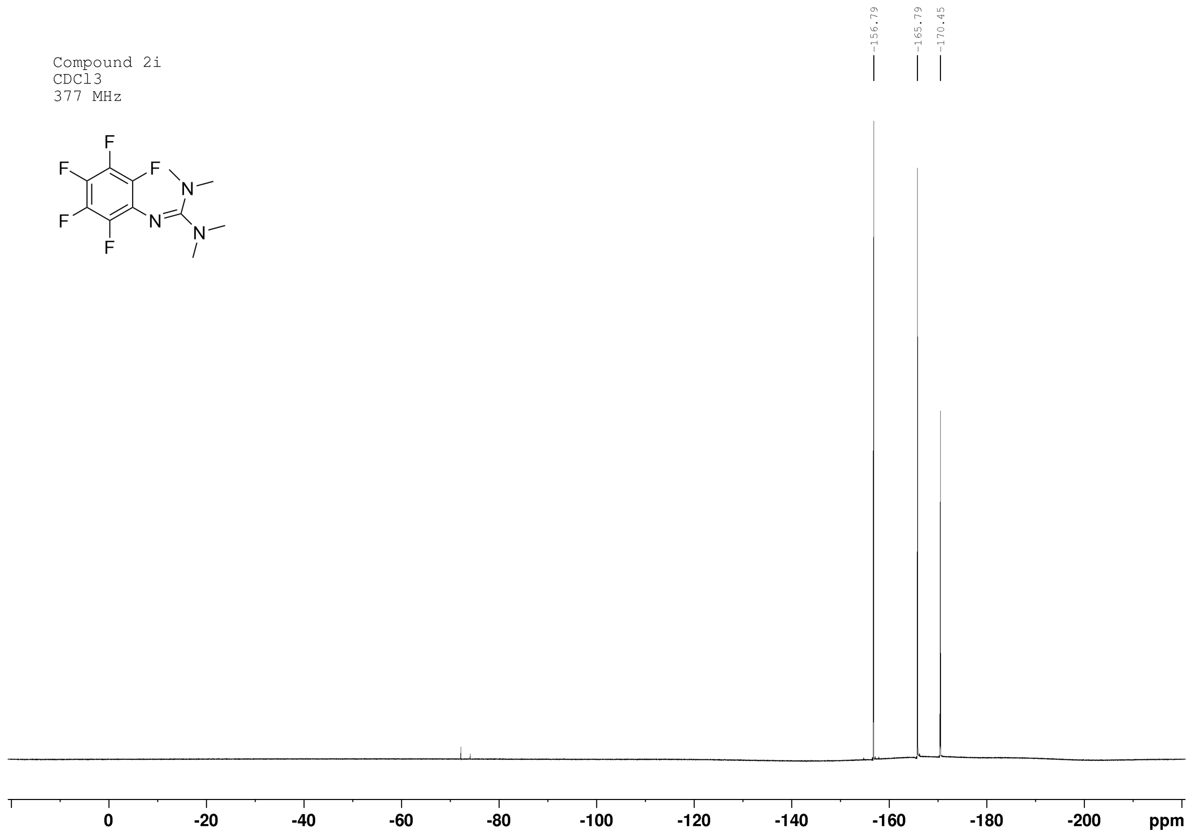
-156.79
-165.79
-170.45
Compound 2i
CDCl3
377 MHz
0 -20 -40 -60 -80 -100 -120 -140 -160 -180 -200 ppm

2j

1,1,3,3-Tetramethyl-2-(4-(4,4,5,5-tetramethyl-1,3,2-dioxaborolan-2-yl)phenyl)guanidine

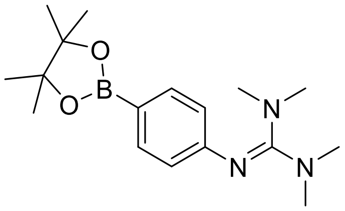

Condition A with DMF. Workup 2. Brown solid. Yield 486 mg (36 %).

1H NMR (400 MHz, $CDCl_3$) δ [ppm]: 7.66 (d, J = 8.2, 2H), 6.69 (d, J = 8.2, 2H), 2.71 (s, 12H), 1.33 (s, 12H).

13C{1H} NMR (101 MHz, CDCl3) δ [ppm]: 160.2, 154.3, 136.4, 135.8, 121.0, 83.3, 39.7, 24.9

HRMS (ESI-TOF) m/z: [M+H]+ Calcd for C17H29BN3O2 318.2347; found 318.2347

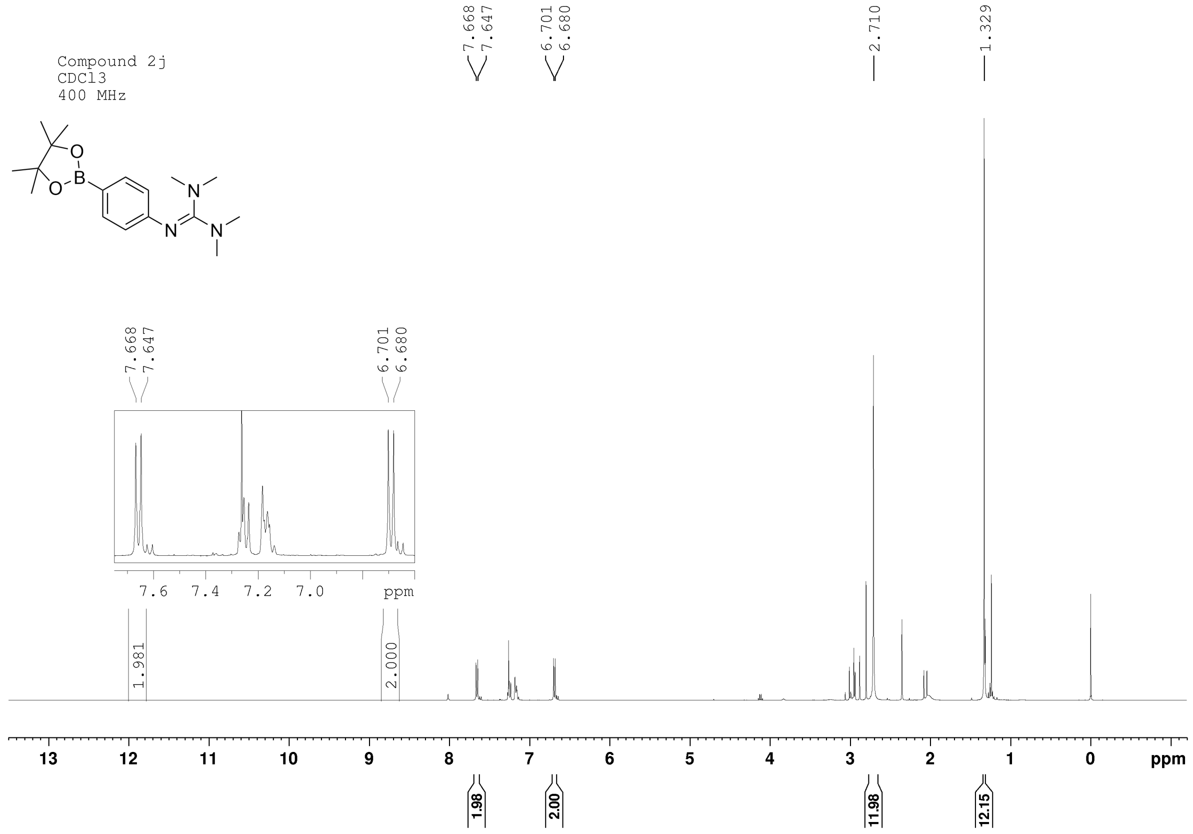

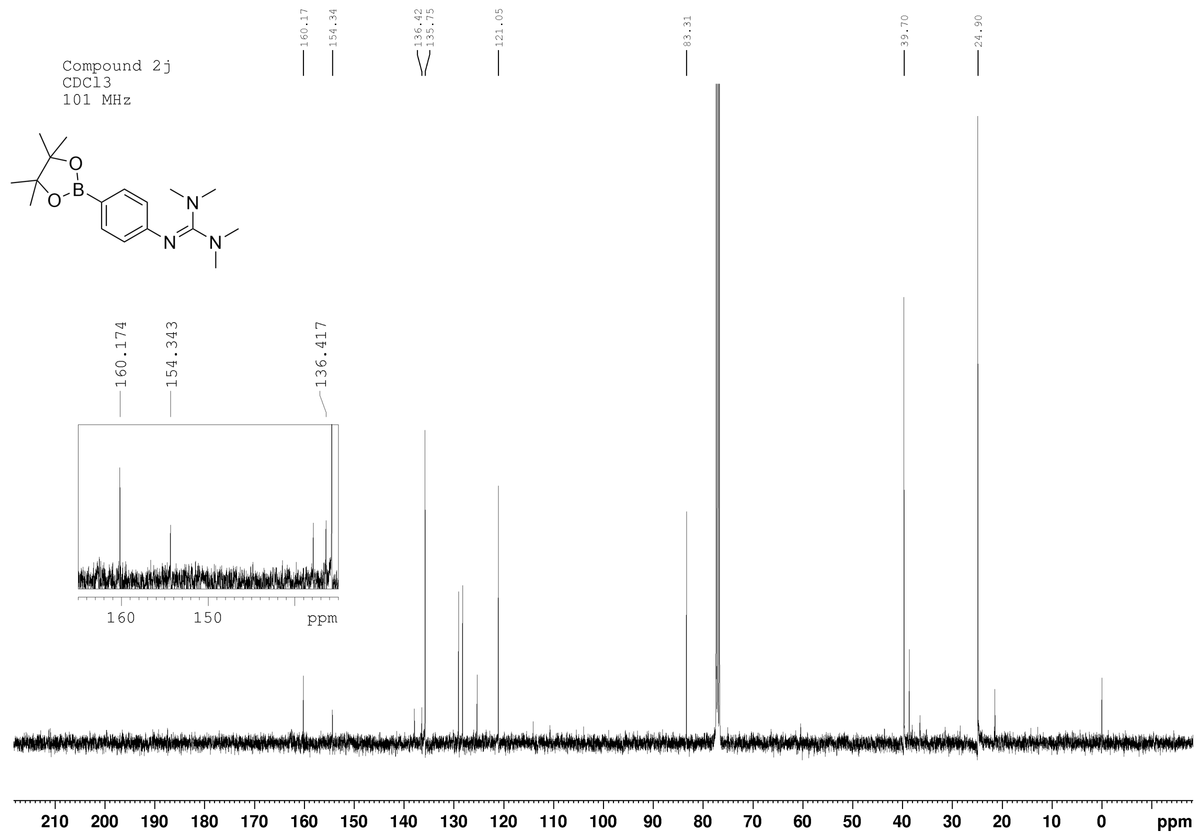

2k

1,1,3,3-Tetramethyl-2-(2,6-dichlorophenyl)guanidine

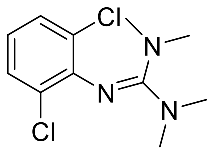

Condition B. Workup 2. Purple solid. Yield 226 mg (43 %).

1H NMR (400 MHz, DMSO-d_6) δ [ppm]: 7.28 (d, J = 8.0, 2H), 6.75 (t, J = 8.0, 1H), 2.65 (s, 12H).

13C{1H} NMR (101 MHz, DMSO-d_6) δ [ppm]: 160.6, 147.0, 128.5, 127.2, 120.7, 39.1

HRMS (ESI-TOF) m/z: [M+H]+ Calcd for $C_{11}H_{16}Cl_2N_3$ 260.0716; found 260.0717

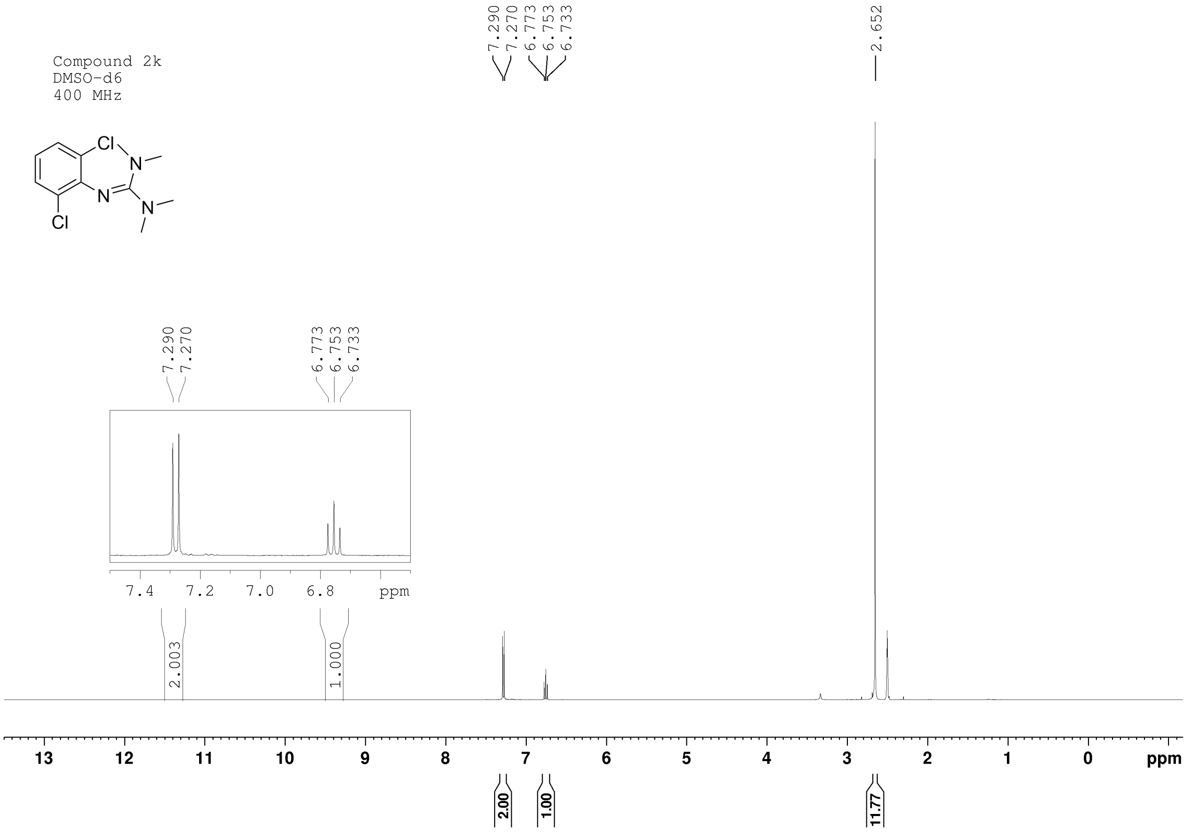
Compound 2k
DMSO-d6
400 MHz
7.290
7.270
6.773
6.753
6.733
2.652
2.003
1.000
2.00
1.00
11.77
ppm

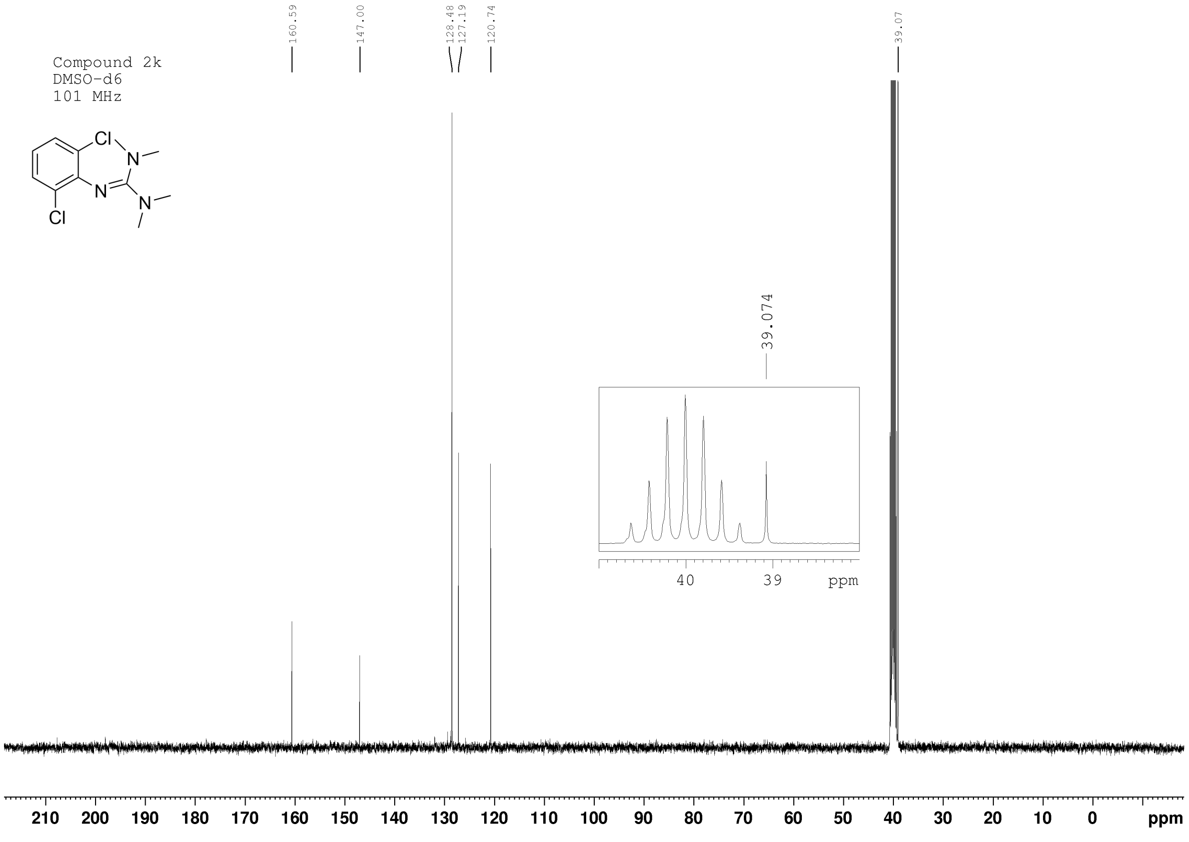
Compound 2k
DMSO-d6
101 MHz
160.59
147.00
128.48
127.19
120.74
39.07
39.074
ppm

2l

1,1,3,3-Tetramethyl-2-(thiazol-2-yl)guanidine

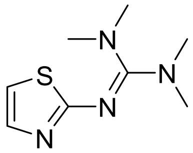

Condition A with ACN, Workup 2. Brown oil. Yield 249 mg (63 %)

1H NMR (400 MHz, $CDCl_3$) δ [ppm]: 7.25 (d, J = 3.7, 1H), 6.67 (d, J = 3.7, 1H), 2.82 (s, 12H).

13C{1H} NMR (101 MHz, CDCl3) δ [ppm]: 173.6, 162.6, 139.2, 110.9, 39.7

HRMS (ESI-TOF) m/z: [M+H]+ Calcd for C8H15N4S 199.1012; found 199.1011

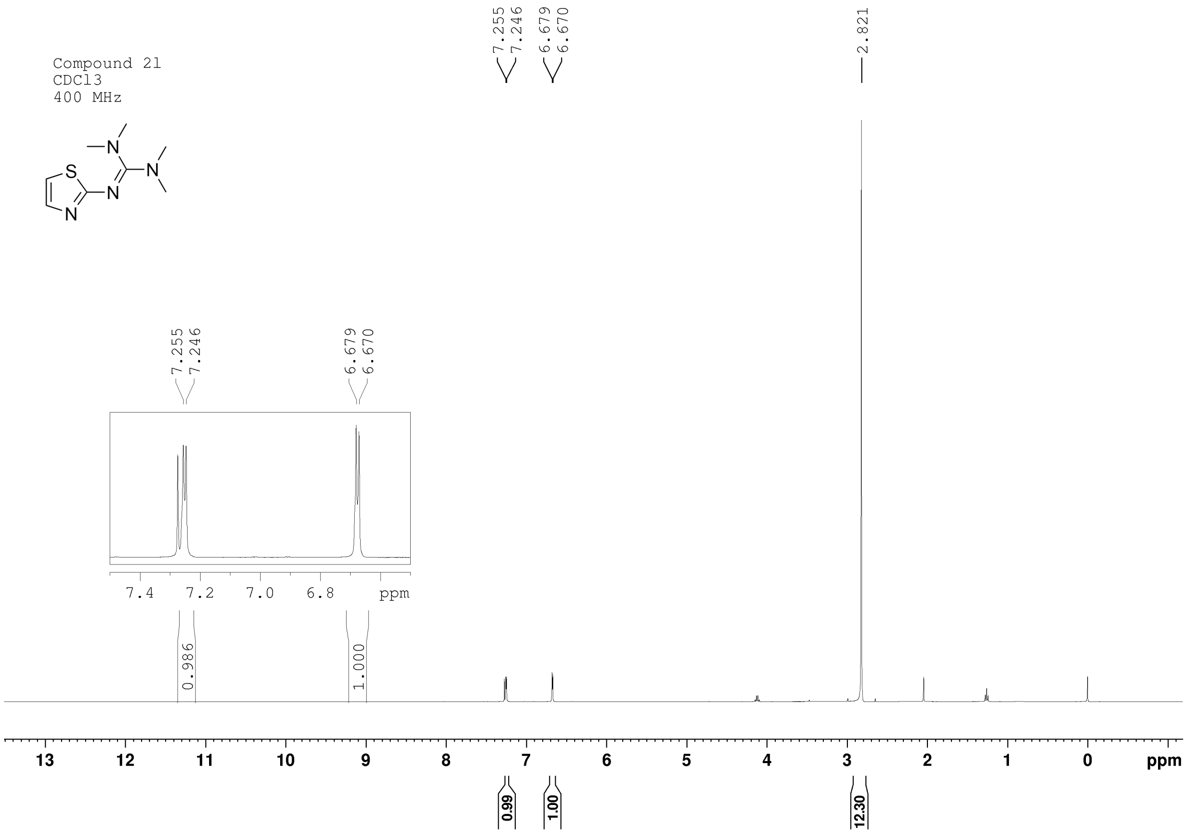

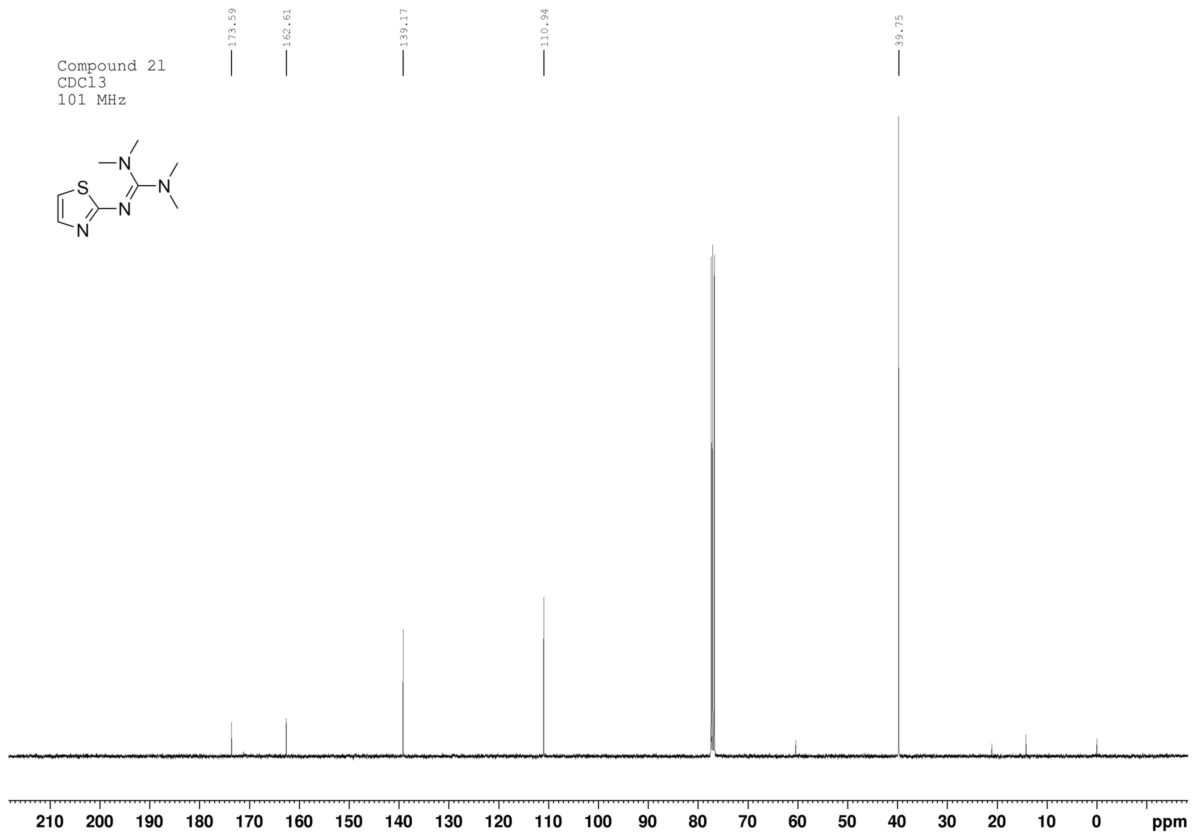

2m

1,1,3,3-Tetramethyl-2-(4-fluorobenzyl)guanidine

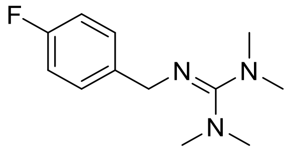

Condition A with ACN. Workup 2. Light yellow oil. Yield 316 mg (71 %).

1H NMR (400 MHz, $CDCl_3$) δ [ppm]: 7.33 (dd, J = 8.5, 5.7, 2H), 6.97 (t, J = 8.88, 2H), 4.34 (s, 2H), 2.76 (d, J = 24.7, 12H).

13C{1H} NMR (101 MHz, CDCl3) δ [ppm]: 161.3 (d, J = 242.6), 160.9, 139.4 (d, J = 3.0), 128.4 (d, J = 7.8), 114.6 (d, J = 21.0), 52.4, 39.3 (d, J = 78.2)

19F{1H} NMR (377 MHz, CDCl3) δ [ppm]: -118.2

HRMS (ESI-TOF) m/z: [M+H]+ Calcd for C12H19FN3 224.1558; found 224.1560

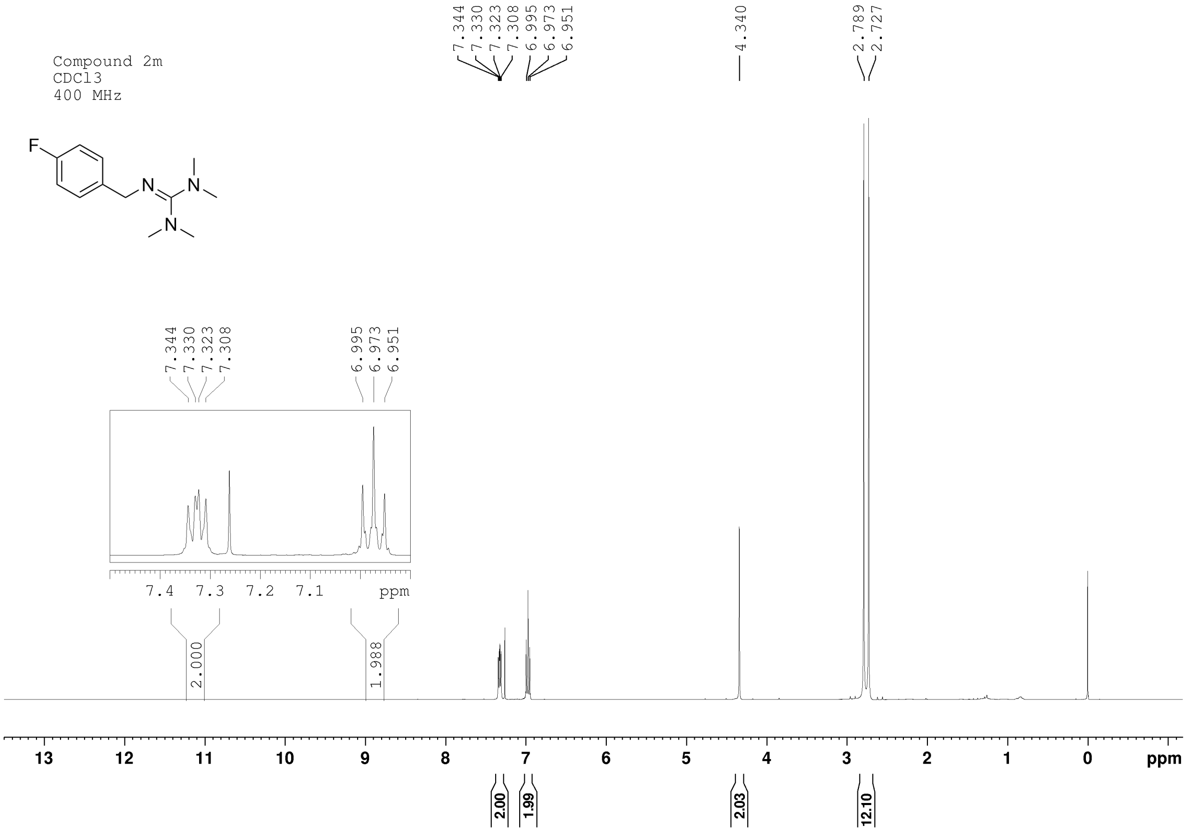
Compound 2m
CDCl3
400 MHz
F
N
N
N
7.344
7.330
7.323
7.308
6.995
6.973
6.951
4.340
2.789
2.727
7.4 7.3 7.2 7.1 ppm
2.000
1.988
13 12 11 10 9 8 7 6 5 4 3 2 1 0 ppm
2.00
1.99
2.03
12.10

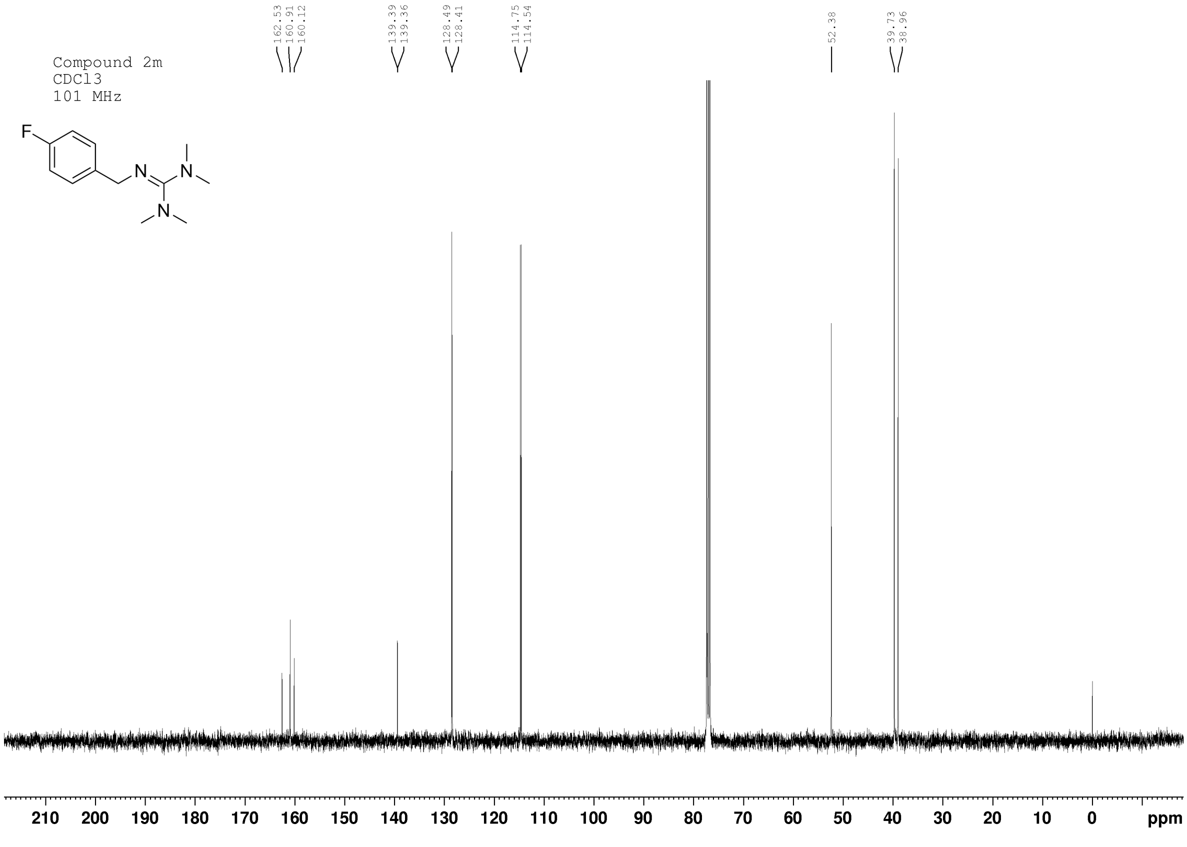
Compound 2m
CDCl3
101 MHz
F
N
N
N
162.53
160.91
160.12
139.39
139.36
128.49
128.41
114.75
114.54
52.38
39.73
38.96
210 200 190 180 170 160 150 140 130 120 110 100 90 80 70 60 50 40 30 20 10 0 ppm

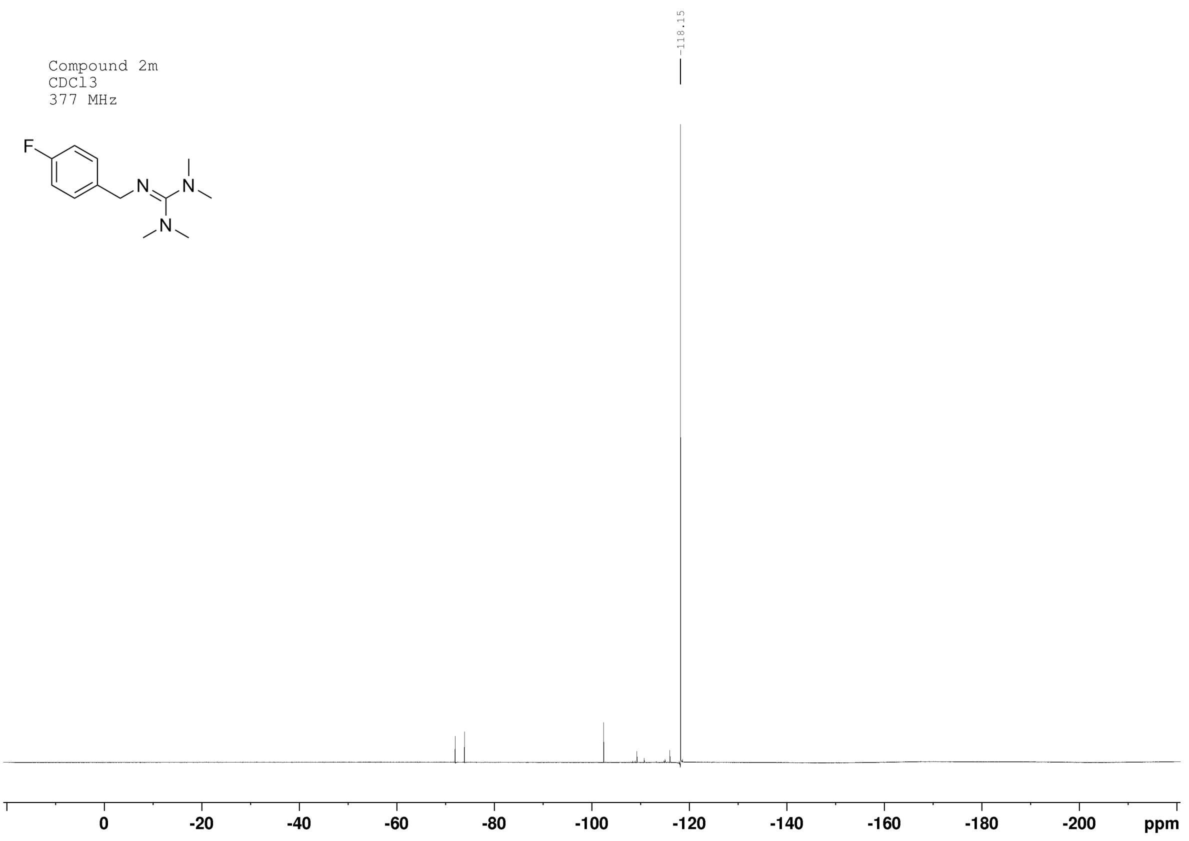

2n

1,1,3,3-Tetramethyl-2-(3,5-bis(trifluoromethyl)benzyl)guanidine

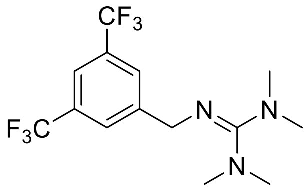

Condition A with DMF. Workup 2. Brown oil. Yield 497 mg (73 %).

1H NMR (400 MHz, $CDCl_3$) δ [ppm]: 7.86 (s, 2H), 7.71 (s, 1H), 4.48 (s, 2H), 2.80 (d, J = 10.2, 12H).

13C{1H} NMR (101 MHz, CDCl3) δ [ppm]: 161.8, 145.9, 131.1 (q, J = 32.8), 127.5 (d, J = 3.2), 123.6 (d, J = 272.6), 120.0 (m), 51.9, 39.4 (d, J = 74.1).

19F{1H} NMR (377 MHz, CDCl3) δ [ppm]: -62.8

HRMS (ESI-TOF) m/z: [M+H]+ Calcd for $C_{14}H_{18}F_6N_3$ 342.1406; found 342.1399

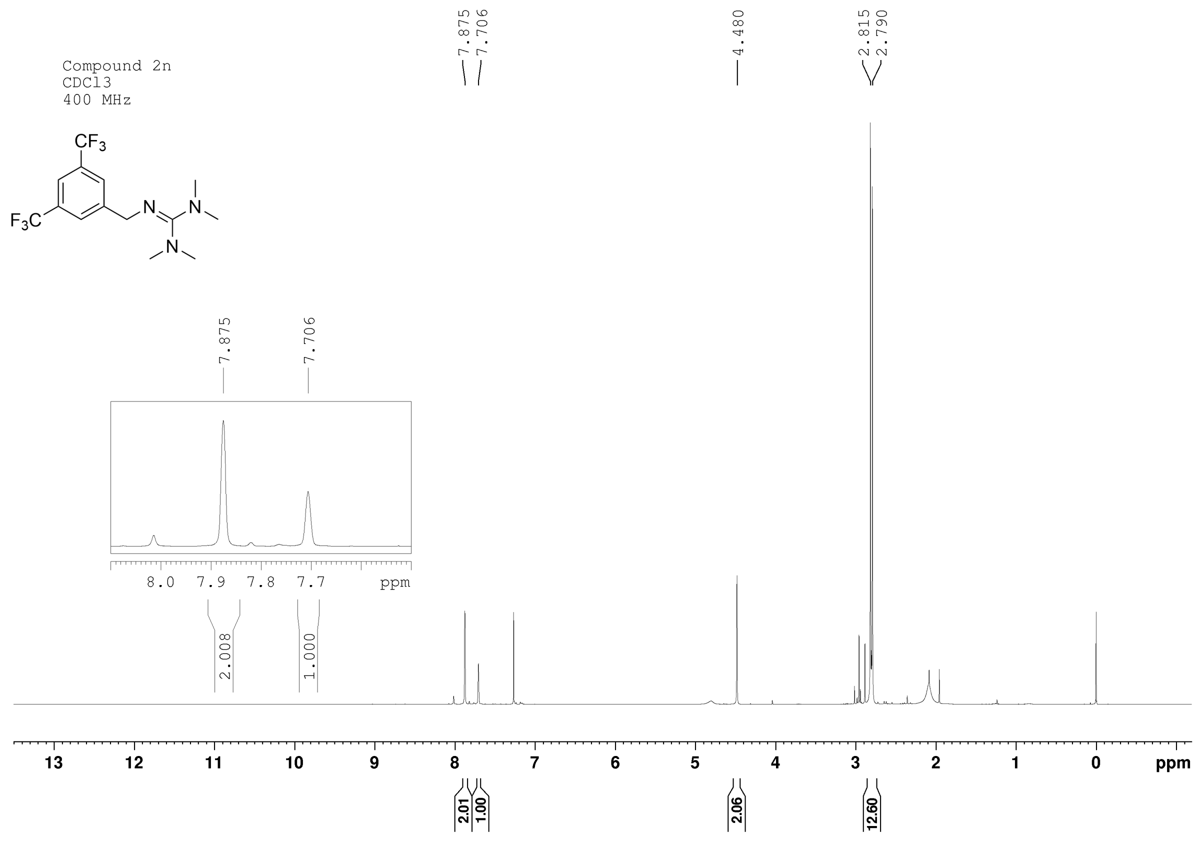
Compound 2n
CDCl3
400 MHz
CF3
F3C
N
N
N
7.875
7.706
4.480
2.815
2.790
2.008
1.000
2.01
1.00
2.06
12.60
ppm

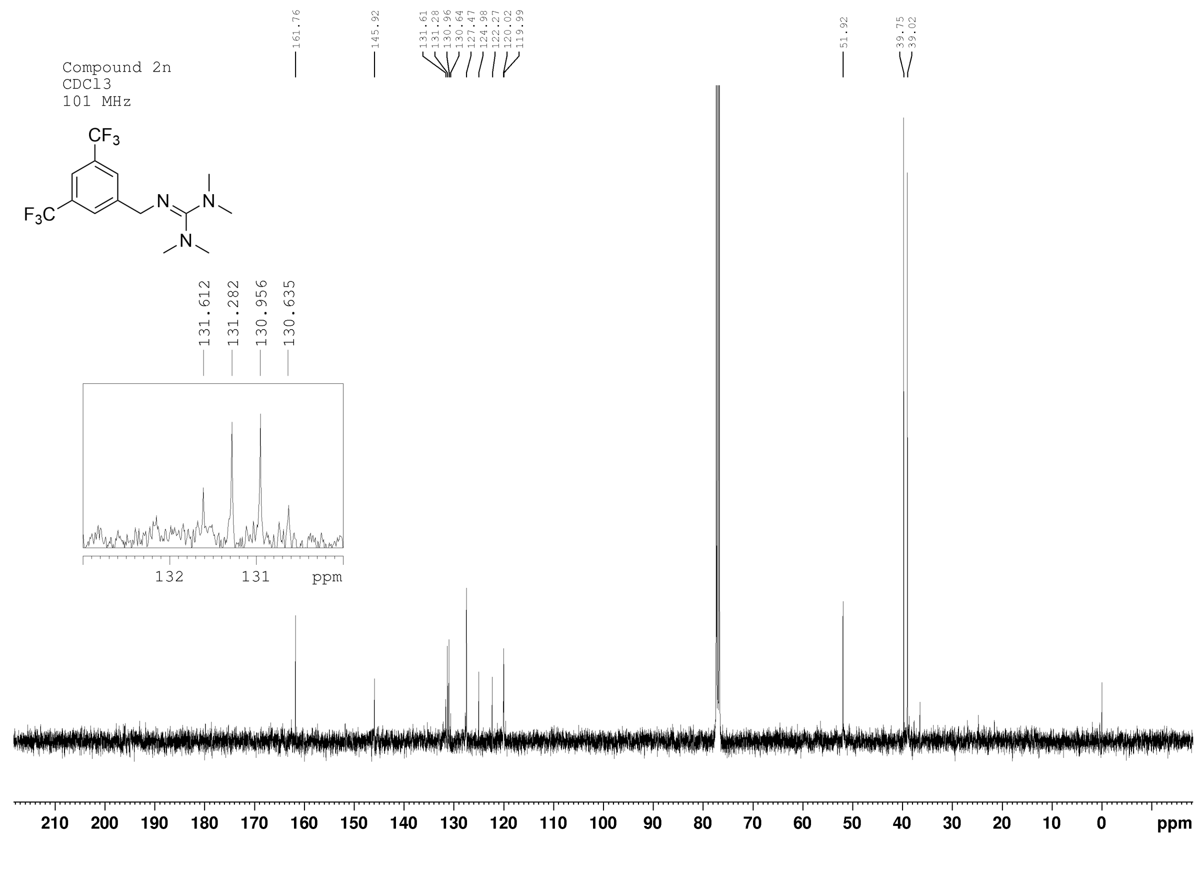
Compound 2n
CDCl3
101 MHz
CF3
F3C
N
N
N
161.76
145.92
131.61
131.28
130.96
130.64
127.47
124.98
122.27
120.02
119.99
51.92
39.75
39.02
131.612
131.282
130.956
130.635
ppm

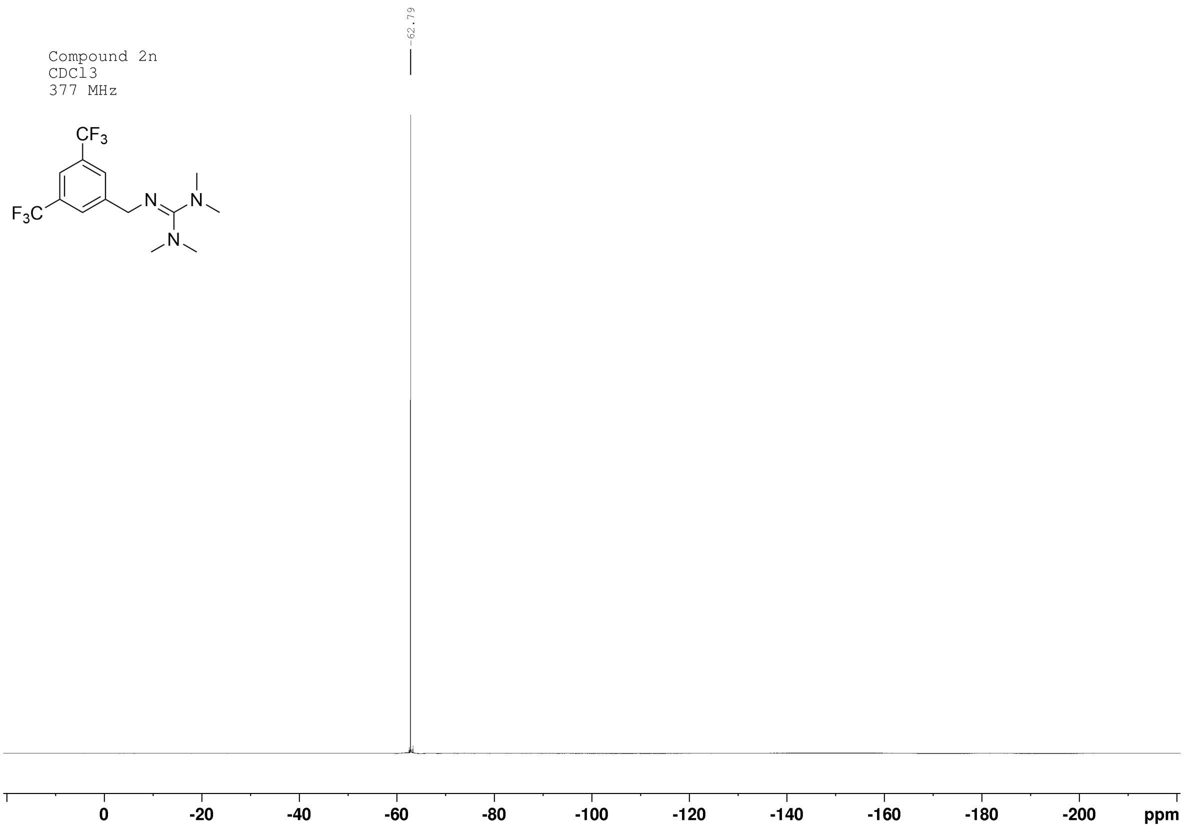

2o

1,1,3,3-Tetramethyl-2-cyclohexylguanidine

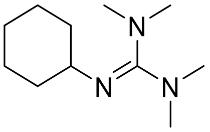

Condition A with DMF. Workup 2 with Na_2SO_4 added to aqueous phases during extractions. Light brown oil. Yield 272 mg (69 %).

1H NMR (400 MHz, $CDCl_3$) δ [ppm]: 2.69 (d, J = 25.0, 12H), 1.80-1.67 (m, 2H), 1.67-1.53 (m, 3H), 1.38-1.11 (m, 5H).

13C{1H} NMR (101 MHz, CDCl3) δ [ppm]: 159.2, 56.4, 40.0, 37.3 (bd, J = 352.4), 25.9, 25.6.

HRMS (ESI-TOF) m/z: [M+H]+ Calcd for C11H24N3 198.1965; found 198.1957

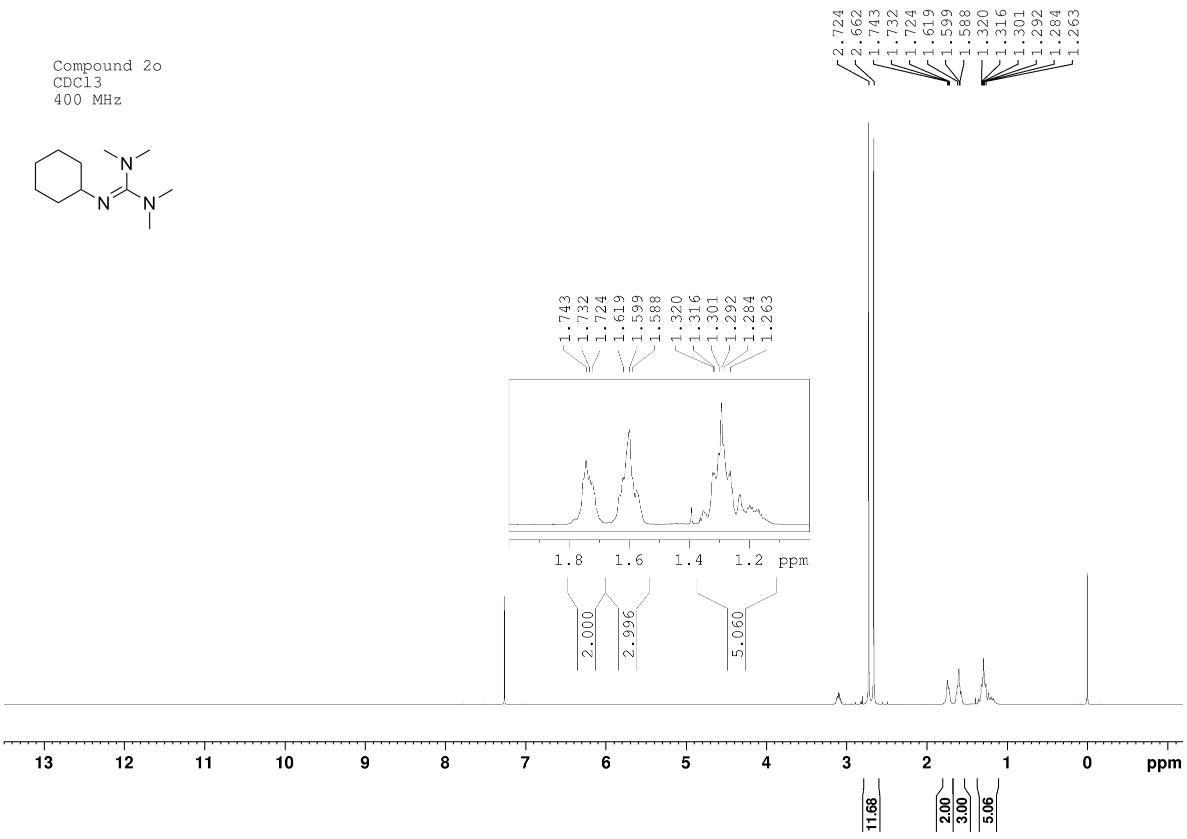
Compound 2o
CDCl3
400 MHz
N
N
N
2.724
2.662
1.743
1.732
1.724
1.619
1.599
1.588
1.320
1.316
1.301
1.292
1.284
1.263
2.000
2.996
5.060
1.8 1.6 1.4 1.2 ppm
11.68
2.00
3.00
5.06
13 12 11 10 9 8 7 6 5 4 3 2 1 0 ppm

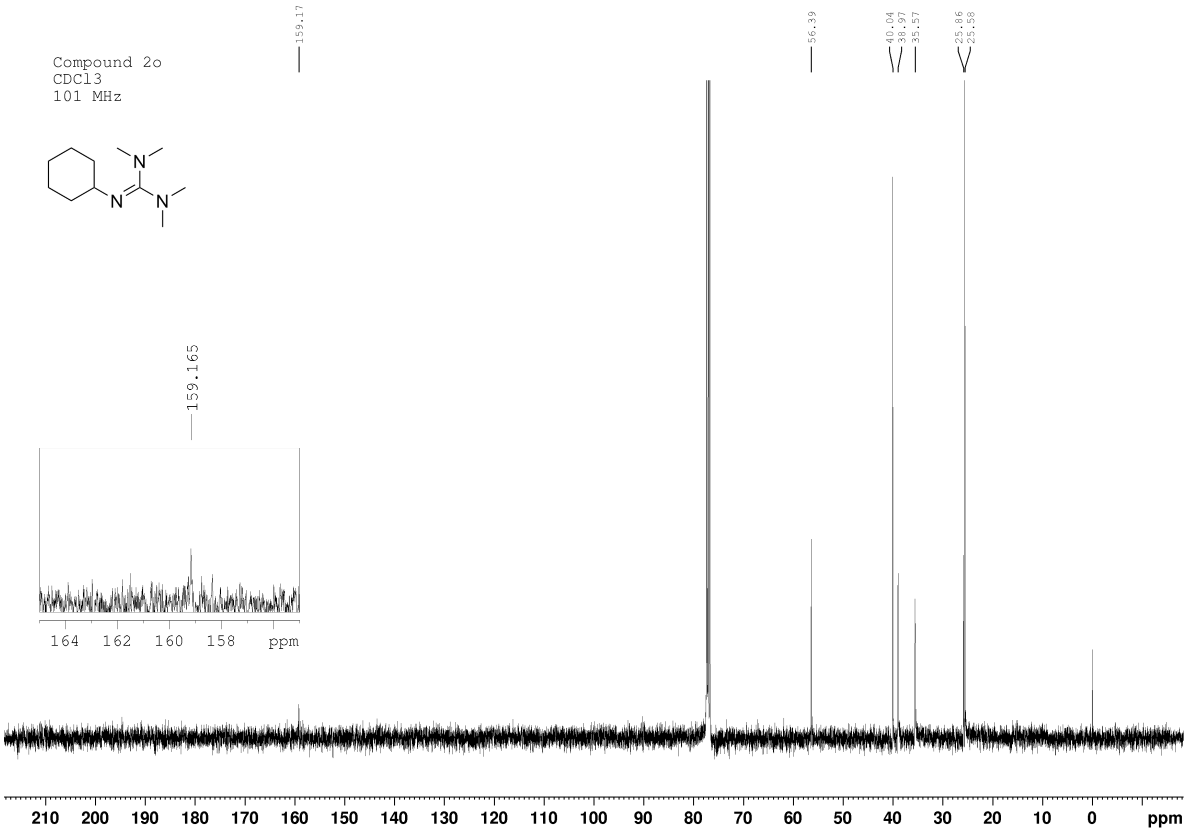
Compound 2o
CDCl3
101 MHz
N
N
N
159.17
56.39
40.04
38.97
35.57
25.86
25.58
159.165
164 162 160 158 ppm
210 200 190 180 170 160 150 140 130 120 110 100 90 80 70 60 50 40 30 20 10 0 ppm

2q

1,1,3,3-Tetramethyl-2-(thiazol-2-ylmethyl)guanidine

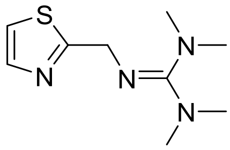

Condition A with DMF. Workup 2. Orange solid. Yield 305 mg (72 %).

1H NMR (400 MHz, $CDCl_3$) δ [ppm]: 7.71 (d, J = 3.3, 1H), 7.22 (d, J = 3.3, 1H), 4.66 (s, 2H), 2.83 (s, 12H).

13C{1H} NMR (101 MHz, CDCl3) δ [ppm]: 176.7, 162.0, 142.4, 118.4, 51.1, 39.4 (bd, J = 53.8).

HRMS (ESI-TOF) m/z: [M+H]+ Calcd for C9H17N4S 213.1168; found 213.1161

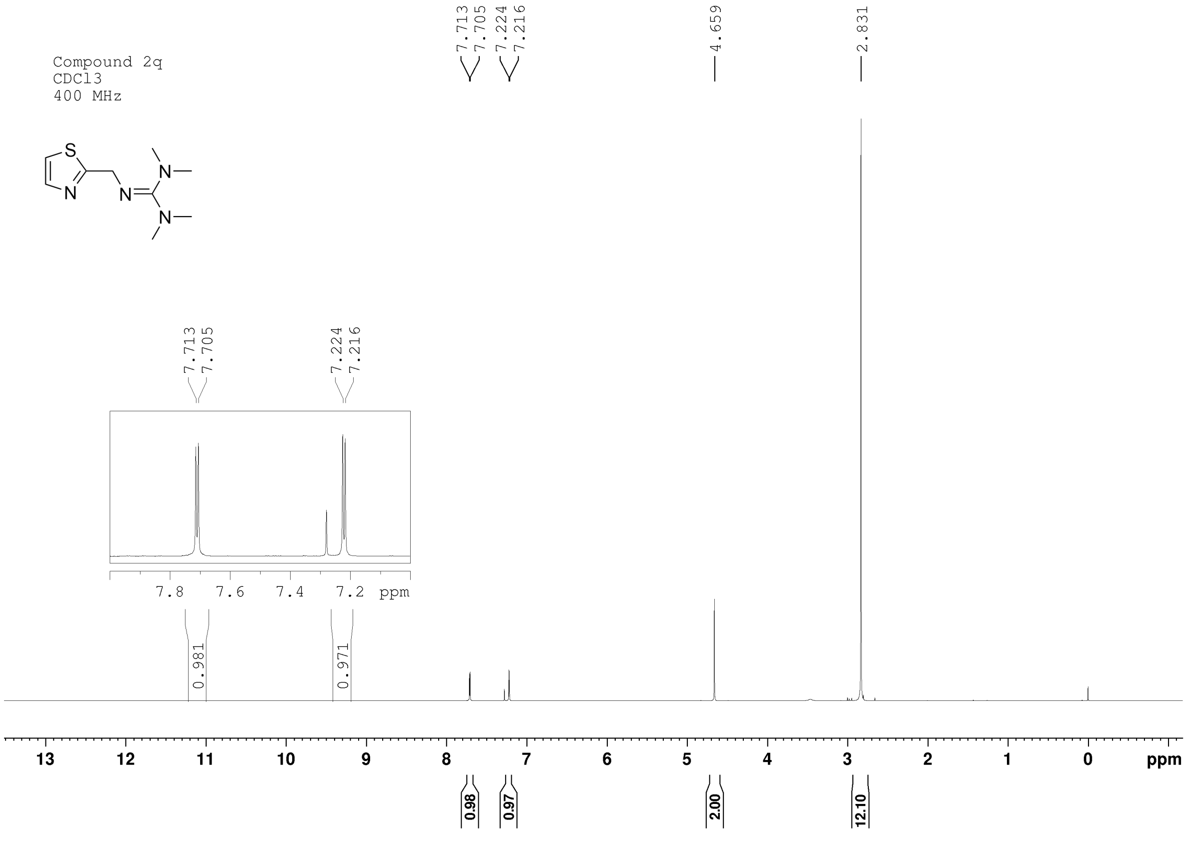

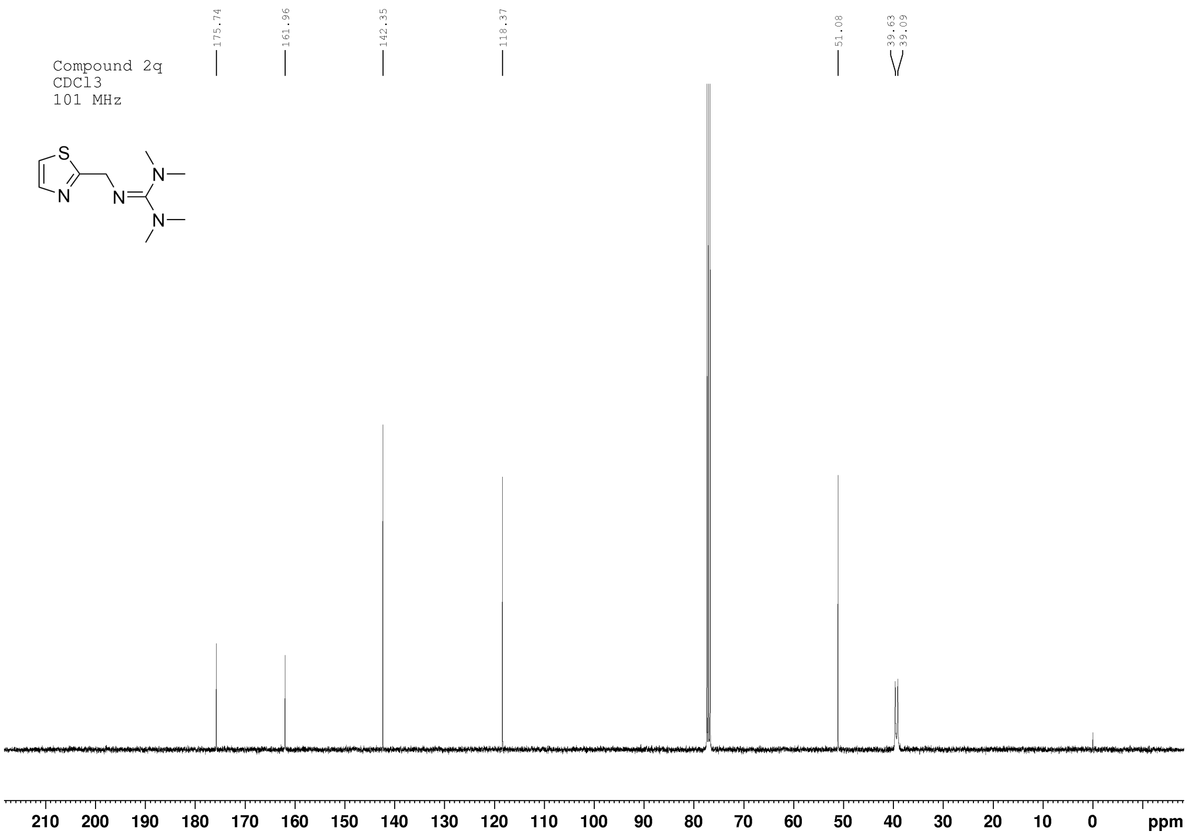

2r

1,1,3,3-Tetramethyl-2-(2,2,2-trifluoroethyl)guanidine

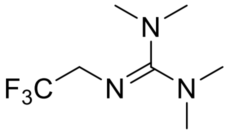

Add the reaction mixture into a separatory funnel. Add 40 ml 2M NaOH solution. Extract the aqueous phase with 3 x 30 ml toluene:EtOAc (3:1). Combine the organic phases and dry with anhydrous Na2SO4 and evaporate the solvent. Vacuum distillation of the residues gives a clear liquid. Bp 61-63°C (11 mmHg). Yield 124 mg (32 %).

1H NMR (400 MHz, $CDCl_3$) δ [ppm]: 3.66 (q, J = 9.5, 2H), 2.74 (d, J = 16.9, 12H).

13C{1H} NMR (101 MHz, CDCl3) δ [ppm]: 163.2, 125.9 (q, J = 277.1), 52.0 (q, J = 30.3), 39.1 (d, J = 87.1)

19F{1H} NMR (377 MHz, $CDCl_3$) δ [ppm]: -72.8

HRMS (ESI-TOF) m/z: [M+H]+ Calcd for C7H15F3N3 198.1213; found 198.1210

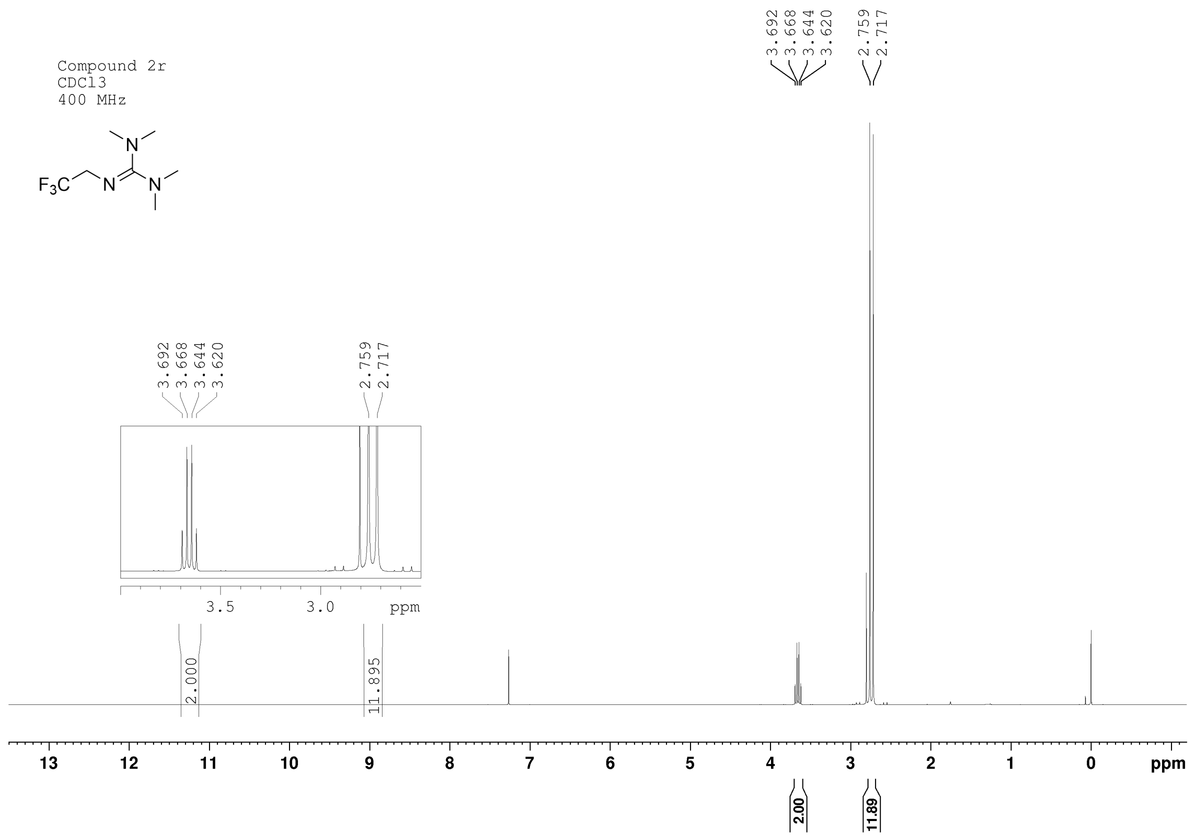
Compound 2r
CDCl3
400 MHz
F3C N N N
3.692
3.668
3.644
3.620
2.759
2.717
3.5
3.0
ppm
2.000
11.895
13 12 11 10 9 8 7 6 5 4 3 2 1 0 ppm
2.00
11.89

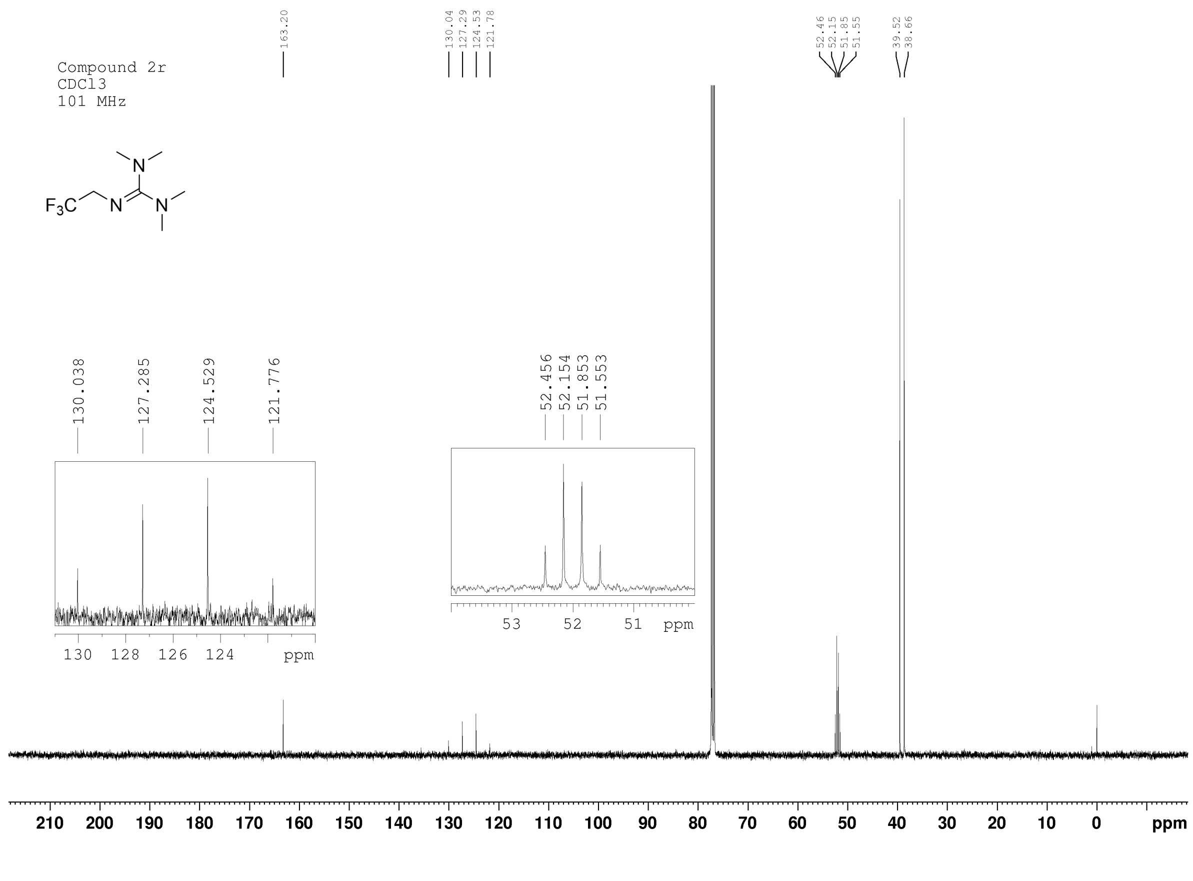
Compound 2r
CDCl3
101 MHz
F3C N N N
163.20
130.04
127.29
124.53
121.78
52.46
52.15
51.85
51.55
39.52
38.66
130.038
127.285
124.529
121.776
130 128 126 124 ppm
52.456
52.154
51.853
51.553
53 52 51 ppm
210 200 190 180 170 160 150 140 130 120 110 100 90 80 70 60 50 40 30 20 10 0 ppm

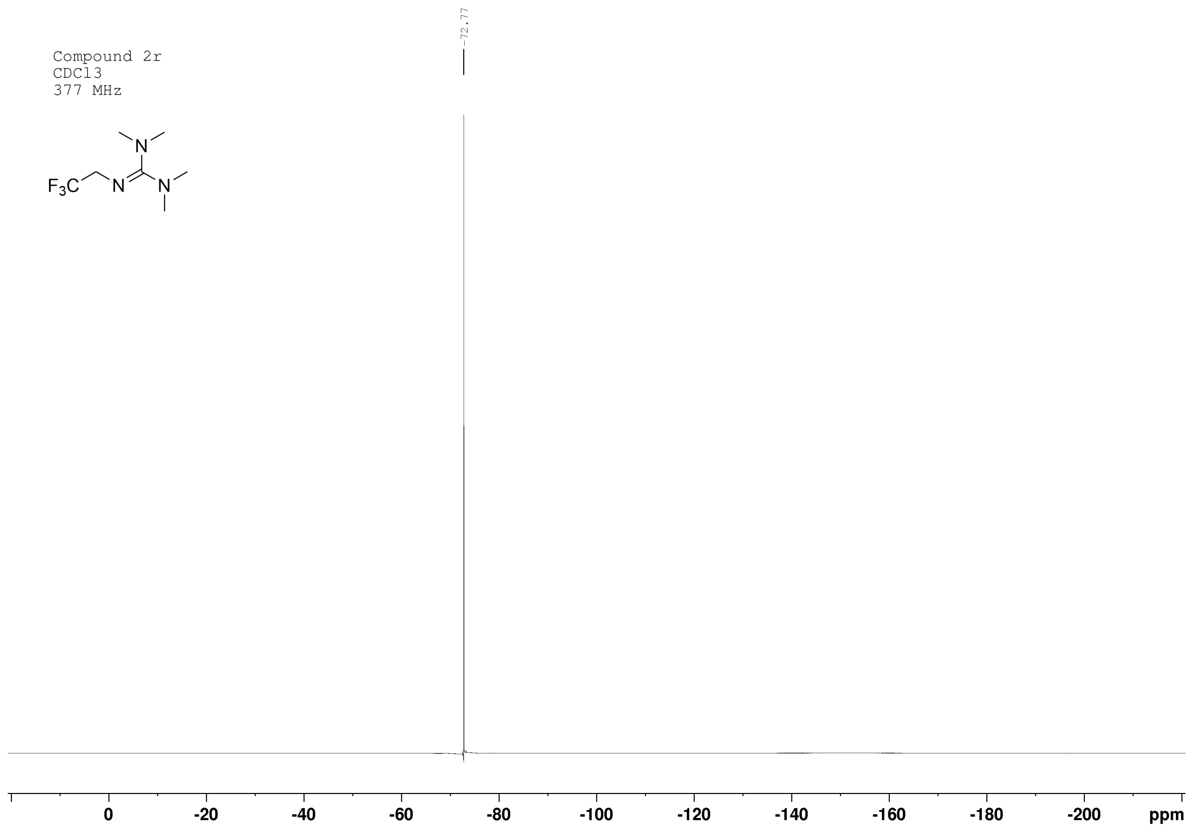

2s

1,1,3,3-Tetramethyl-2-(4,4-difluorocyclohexyl)guanidine

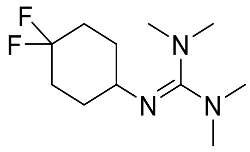

Condition A with DMF. Workup 2 with Na_2SO_4 added to aqueous phases during extractions. Off-white oil. Yield 431 mg (92 %).

1H NMR (400 MHz, DMSO-d_6) δ [ppm]: 3.48-3.37 (m, 1H), 2.62 (d, J = 48.8, 12H), 2.15-1.99 (m, 2H), 1.90-1.73 (m, 2H), 1.65-1.55 (m, 2H), 1.50-1.38 (m, 2H).

13C{1H} NMR (101 MHz, DMSO-d_6) δ [ppm]: 158.4, 129.0 (d, J = 69.9), 126.5 (d, J = 147.4), 51.9, 39.0, 31.4 (t, J = 23.5), 31.3 (t, J = 4.8).

19F{1H} NMR (377 MHz, DMSO-d_6) δ [ppm]: -94.4 (bs)

HRMS (ESI-TOF) m/z: [M+H]+ Calcd for $C_{11}H_{22}F_2N_3$ 234.1776; found 234.1779

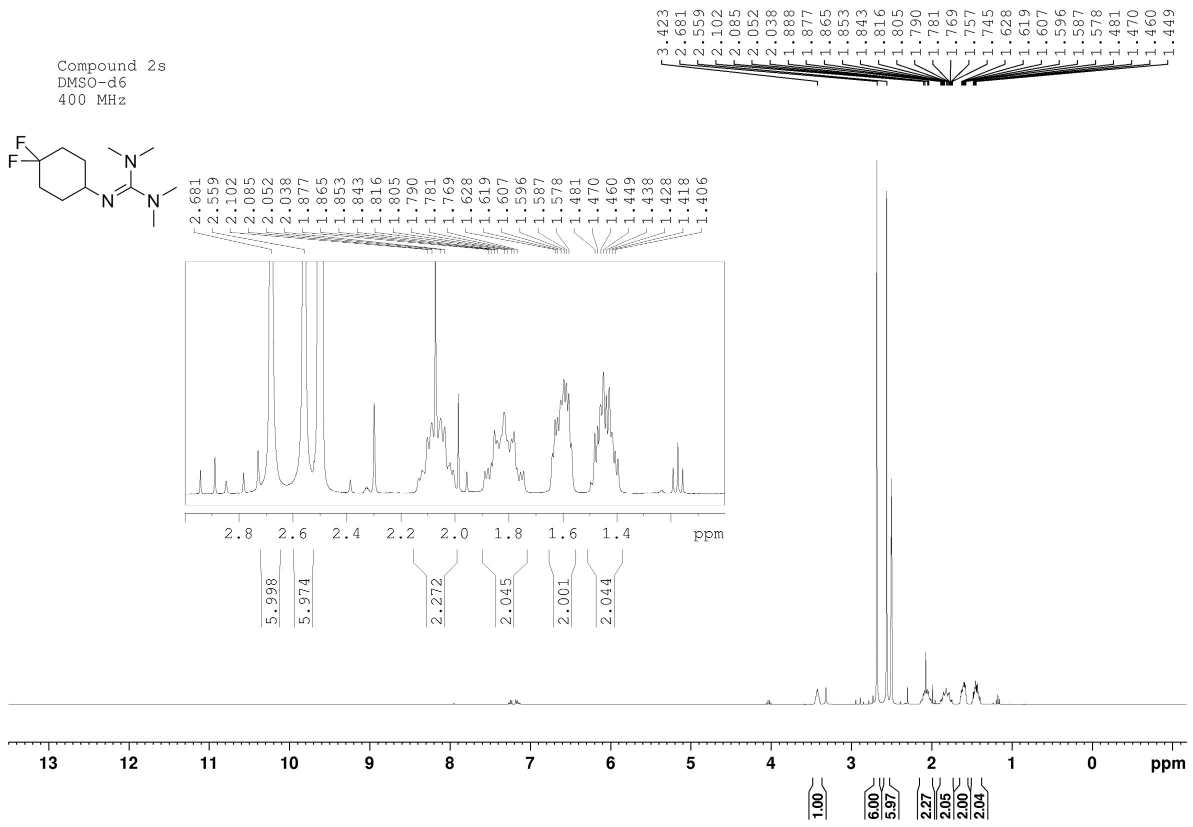
Compound 2s
DMSO-d6
400 MHz

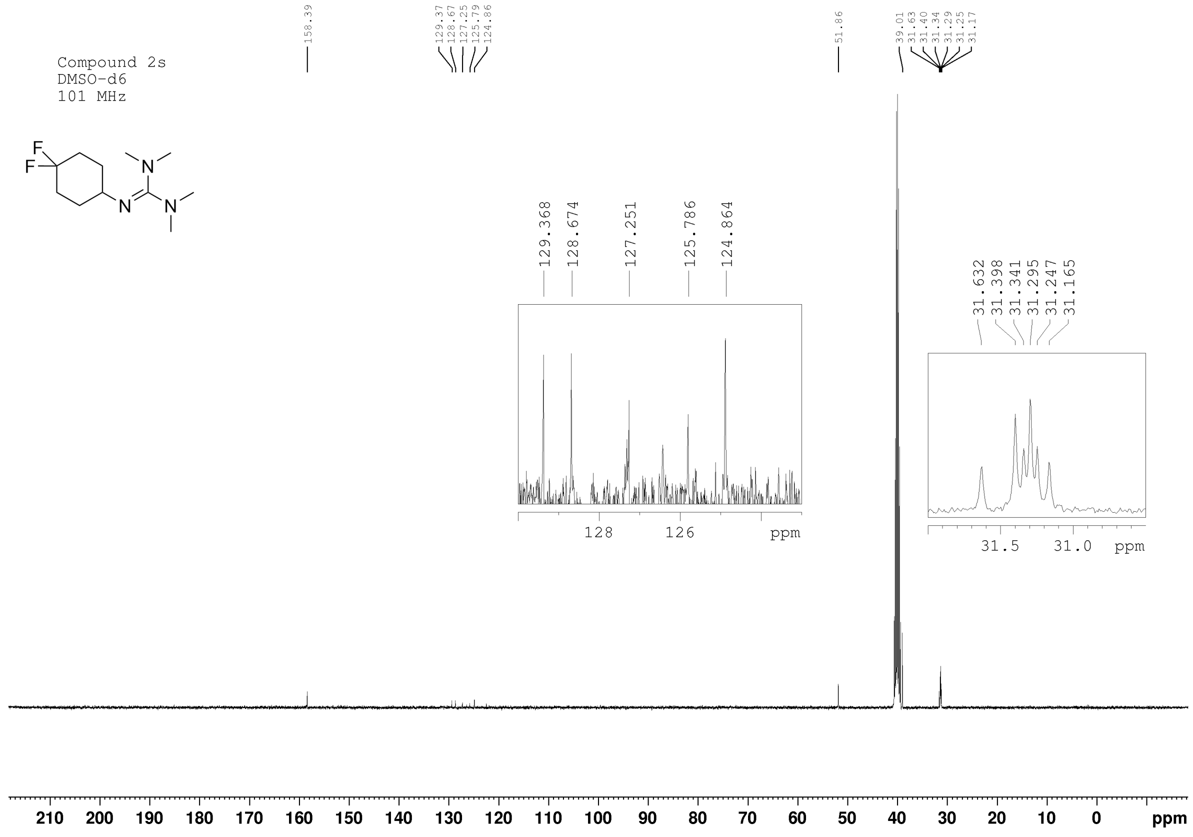
Compound 2s
DMSO-d6
101 MHz

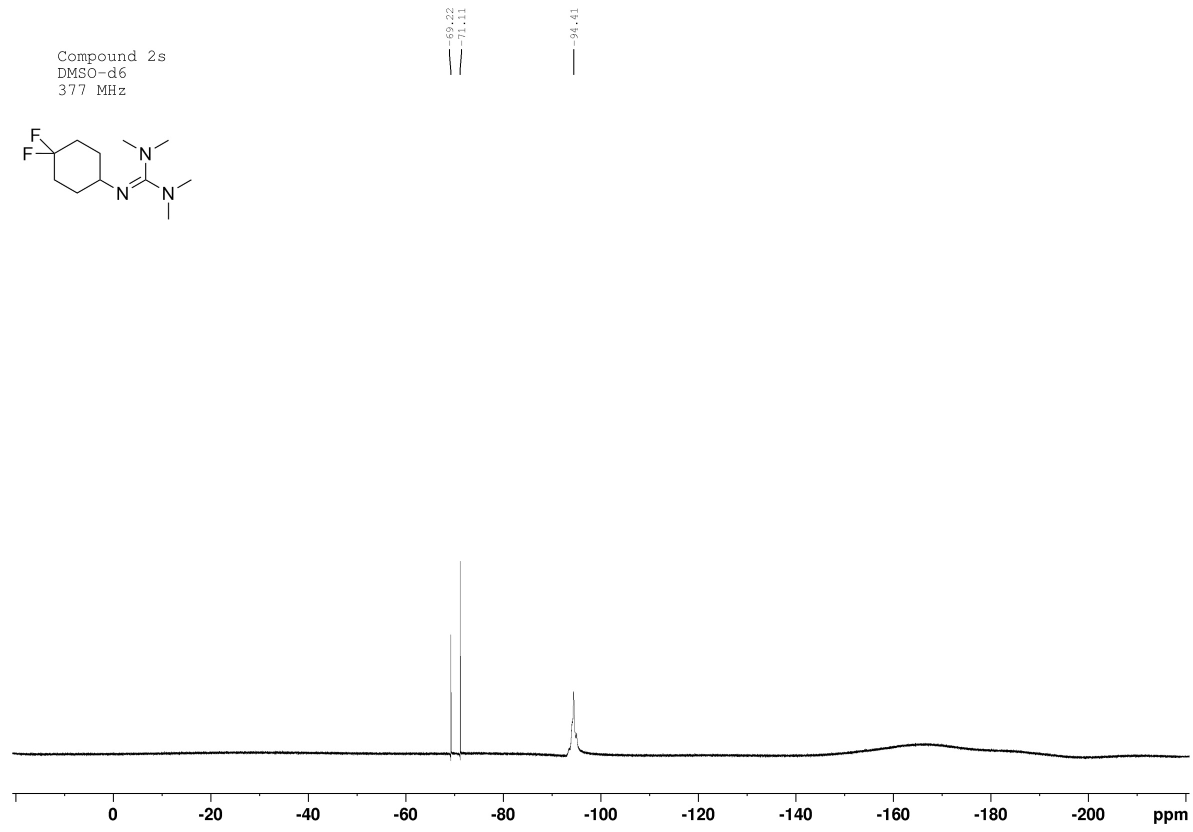

2u

(3aS,7aS)-2-(dimethylamino)-3a,4,5,6,7,7a-hexahydro-1H-benzo[d]imidazol-1-ium

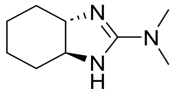

Condition A with DMF. Workup 2 with Na_2SO_4 added to aqueous phases during extractions. Orange solid. Yield 64 mg (19 %).

1H NMR (400 MHz, DMSO-d_6) δ [ppm]: 6.12 (bs, 1H), 2.75 (s, 6H), 2.74-2.67 (m, 2H), 2.03-1.90 (m, 2H), 1.74-1.60 (m, 2H), 1.36-1.15 (m, 4H).

13C{1H} NMR (101 MHz, DMSO-d_6) δ [ppm]: 164.3, 68.5, 38.0, 31.3, 25.0

HRMS (ESI-TOF) m/z: [M+H]+ Calcd for C9H18N3 168.1495; found 168.1499

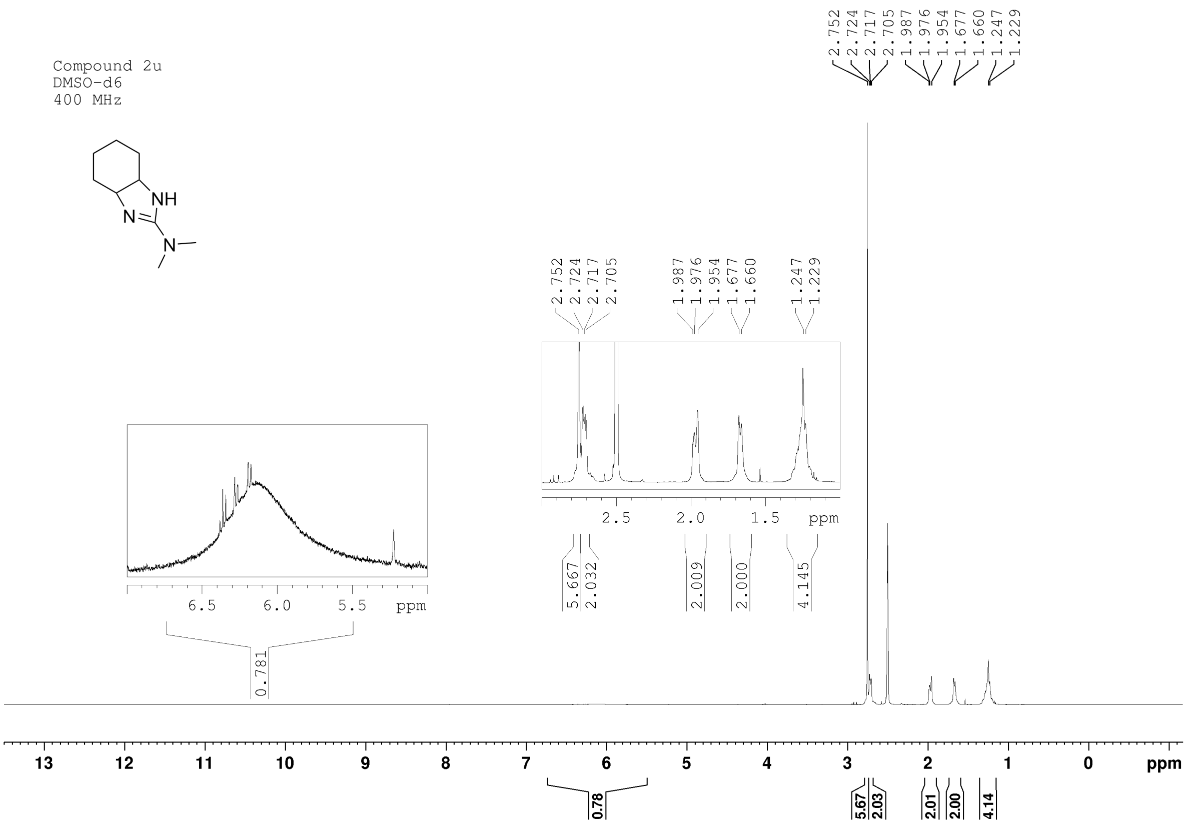
Compound 2u
DMSO-d6
400 MHz
NH
N
2.752
2.724
2.717
2.705
1.987
1.976
1.954
1.677
1.660
1.247
1.229
0.781
5.667
2.032
2.009
2.000
4.145
ppm

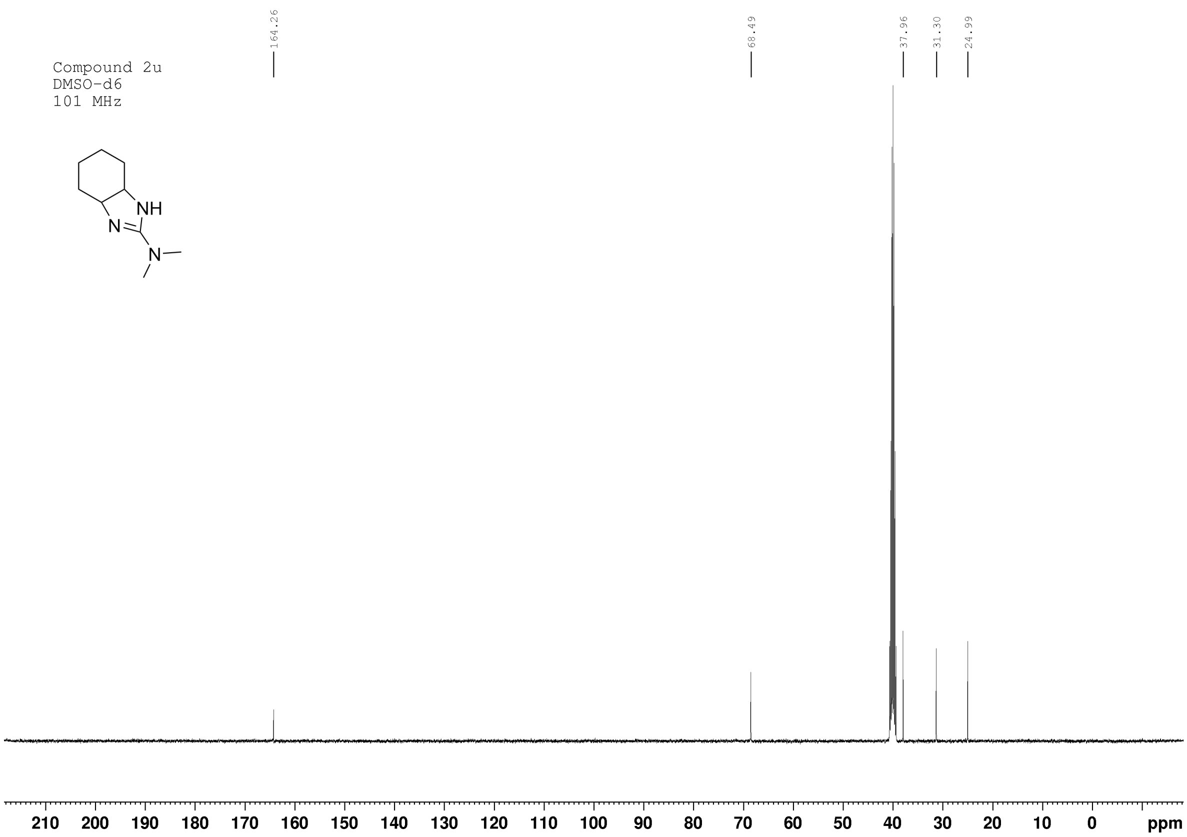
Compound 2u
DMSO-d6
101 MHz
NH
N
164.26
68.49
37.96
31.30
24.99
ppm

2v

1,1,3,3-Tetramethyl-2-(2,6-dichlorobenzamido)guanidine

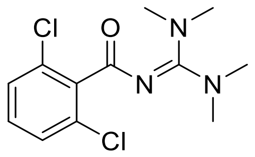

Condition B. Workup 3. Silica gel chromatography was performed using 90:10 EtOAc:MeOH as solvent. White solid. Yield 347 mg (60 %).

1H NMR (400 MHz, DMSO-d_6) δ [ppm]: 7.39 (d, J = 7.9, 2H), 7.27 (dd, J = 8.62, 7.44, 1H), 2.97 (s, 12H).

13C{1H} NMR (101 MHz, DMSO-d_6) δ [ppm]: 168.1, 167.1, 141.4, 130.8, 129.2, 128.5, 40.7

HRMS (ESI-TOF) m/z: [M+H]+ Calcd for $C_{12}H_{16}Cl_2N_3O$ 288.0665; found 288.0676

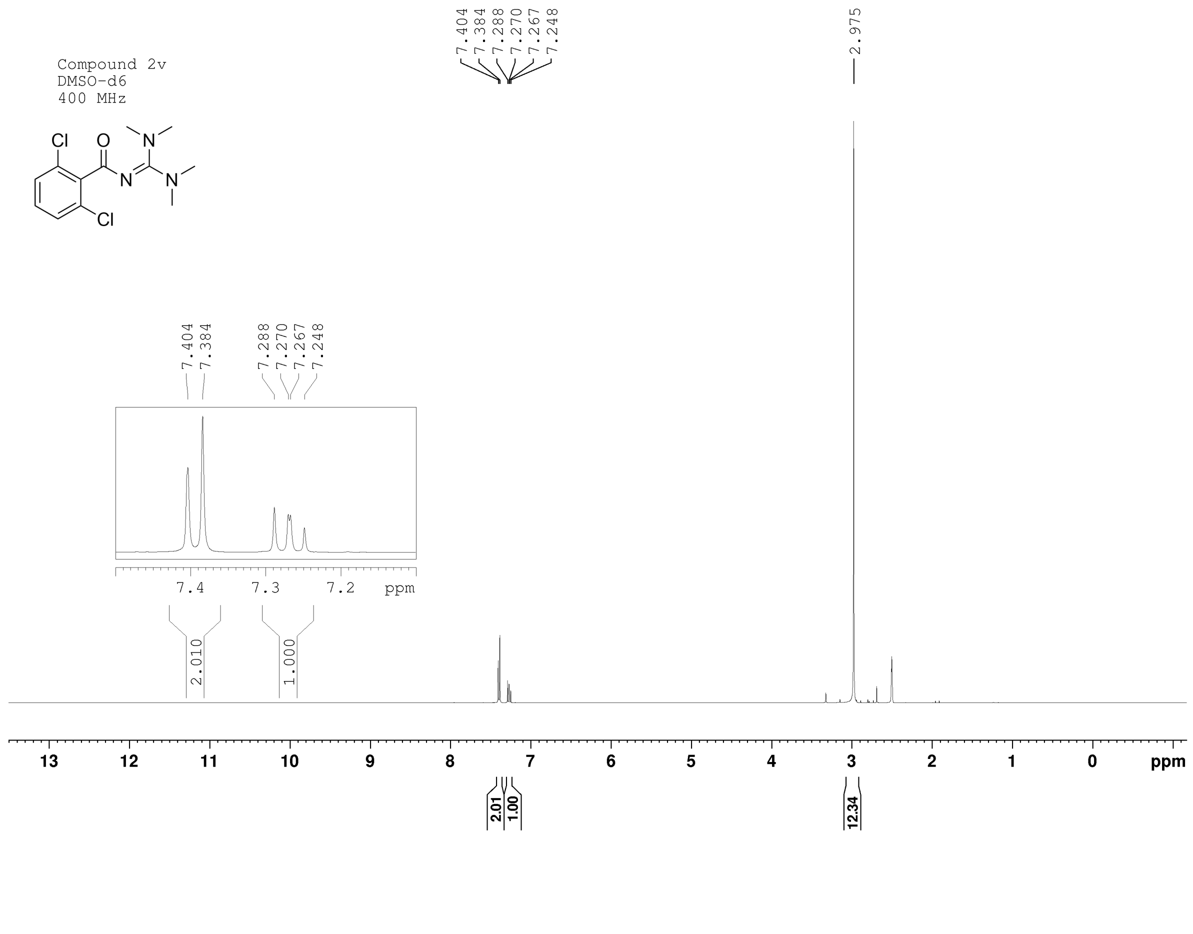

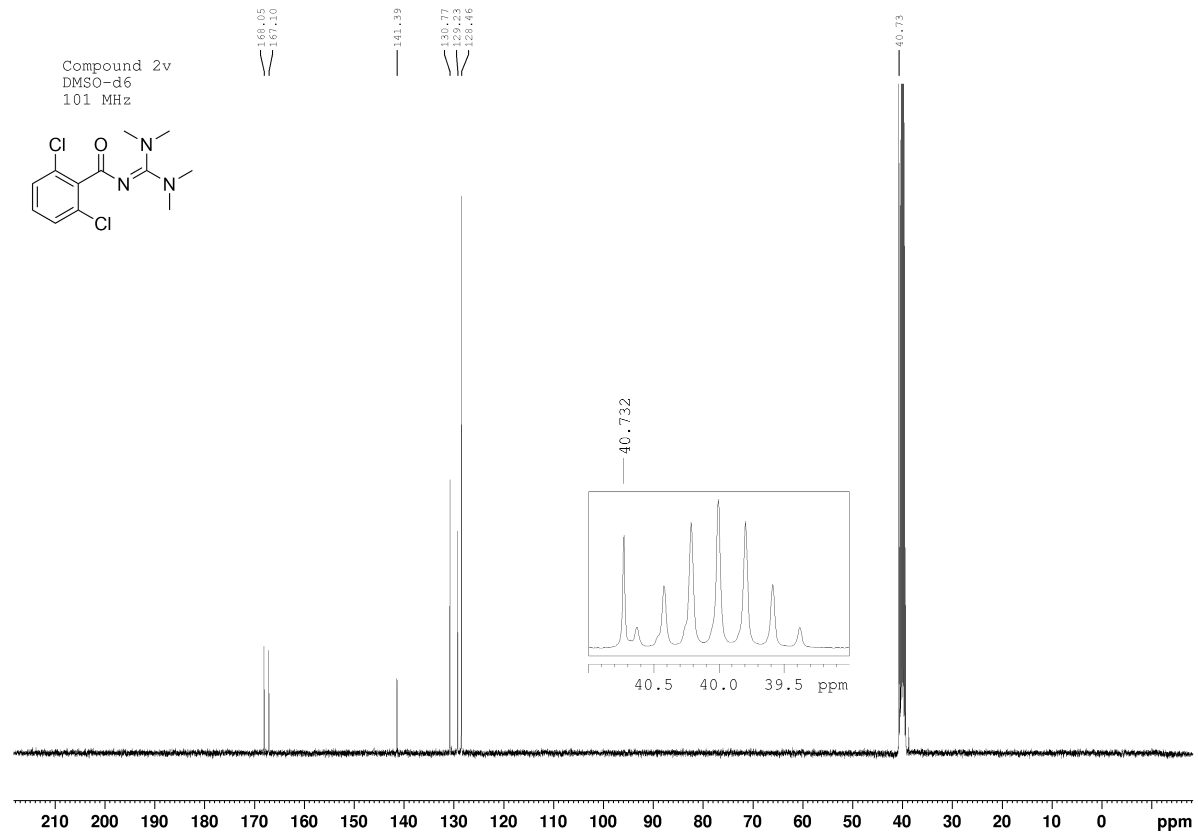

2w

1,1,3,3-Tetramethyl-2-(4-methoxybenzamido)guanidine

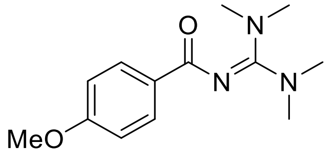

Condition B. Workup 3. Silica gel chromatography was performed using 90:10 EtOAc:MeOH as solvent. White solid. Yield 423 mg (85 %).

1H NMR (400 MHz, DMSO-d_6) δ [ppm]: 7.96 (d, J = 8.8, 2H), 6.91 (d, J = 8.8, 2H), 3.79 (s, 3H), 2.82 (s, 12H).

13C{1H} NMR (101 MHz, DMSO-d_6) δ [ppm]: 171.5, 167.2, 161.7, 131.2, 131.0, 113.3, 55.7, 40.1

HRMS (ESI-TOF) m/z: [M+H]+ Calcd for $C_{13}H_{20}N_3O_2$ 250.1550; found 250.1561

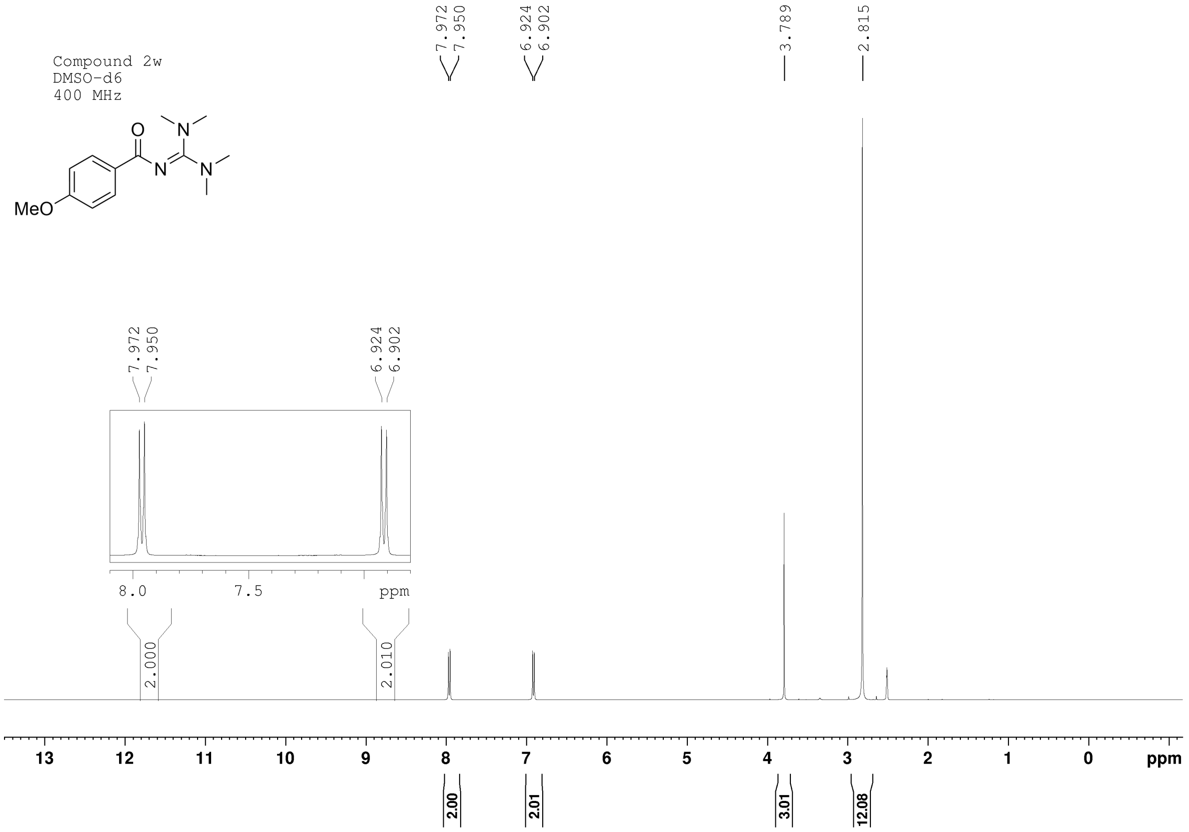
Compound 2w
DMSO-d6
400 MHz
MeO
O
N
N
N
7.972
7.950
6.924
6.902
3.789
2.815
2.000
2.010
8.0
7.5
ppm
13 12 11 10 9 8 7 6 5 4 3 2 1 0 ppm
2.00
2.01
3.01
12.08

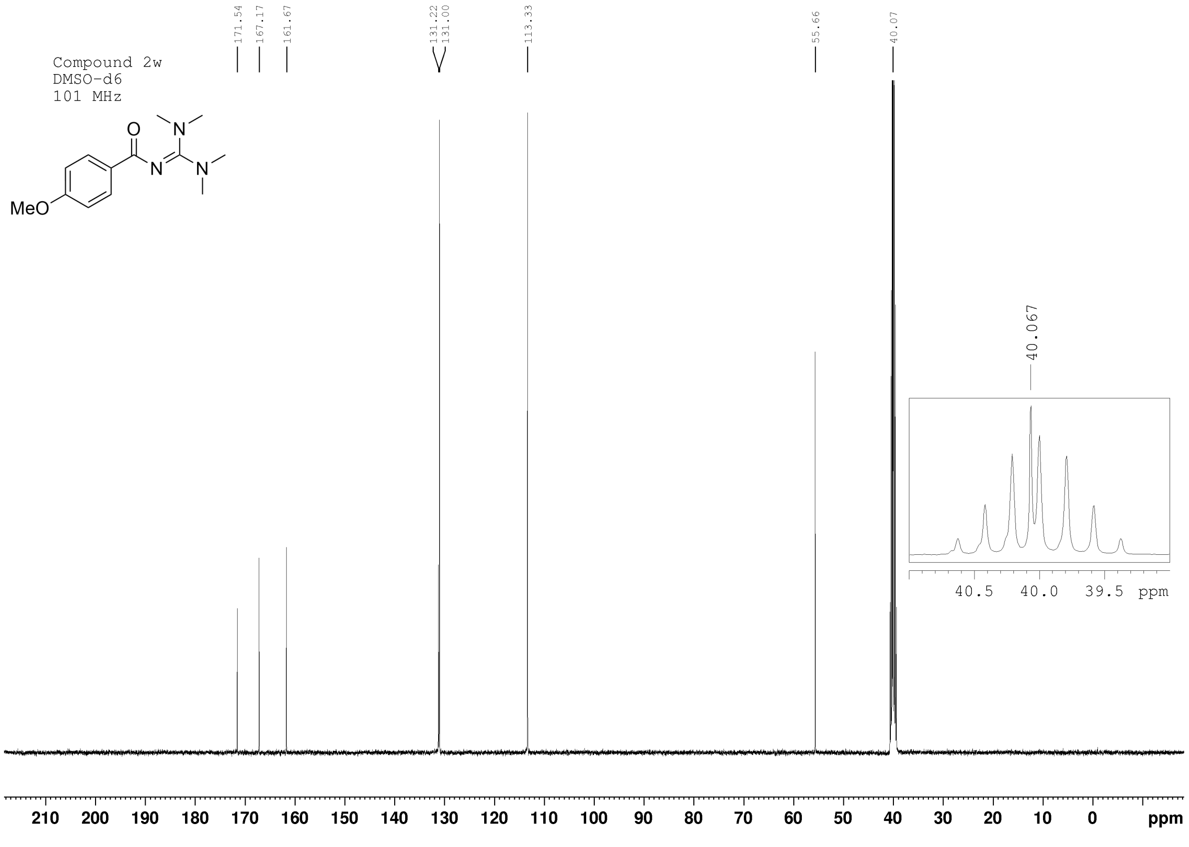
Compound 2w
DMSO-d6
101 MHz
MeO
O
N
N
N
171.54
167.17
161.67
131.22
131.00
113.33
55.66
40.07
40.067
40.5
40.0
39.5
ppm
210 200 190 180 170 160 150 140 130 120 110 100 90 80 70 60 50 40 30 20 10 0 ppm

2x

1,1,3,3-Tetramethyl-2-(4-fluorobenzamido)guanidine

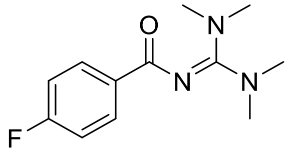

Condition B. Workup 3. Silica gel chromatography was performed using 90:10 EtOAc:MeOH as solvent. White solid. Yield 338 mg (71 %).

1H NMR (400 MHz, DMSO-d_6) δ [ppm]: 8.08-8.01 (m, 2H), 7.21-7.13 (m, 2H), 2.83 (s, 12H).

13C{1H} NMR (101 MHz, DMSO-d_6) δ [ppm]: 170.3, 167.6, 164.2 (d, J = 246.8), 135.2 (d, J = 2.7), 131.6 (d, J = 8.8), 114.9 (d, J = 21.5), 40.1

19F{1H} NMR (377 MHz, DMSO-d_6) δ [ppm]: -111.2

HRMS (ESI-TOF) m/z: [M+H]+ Calcd for C12H17FN3O 238.1350; found 238.1354

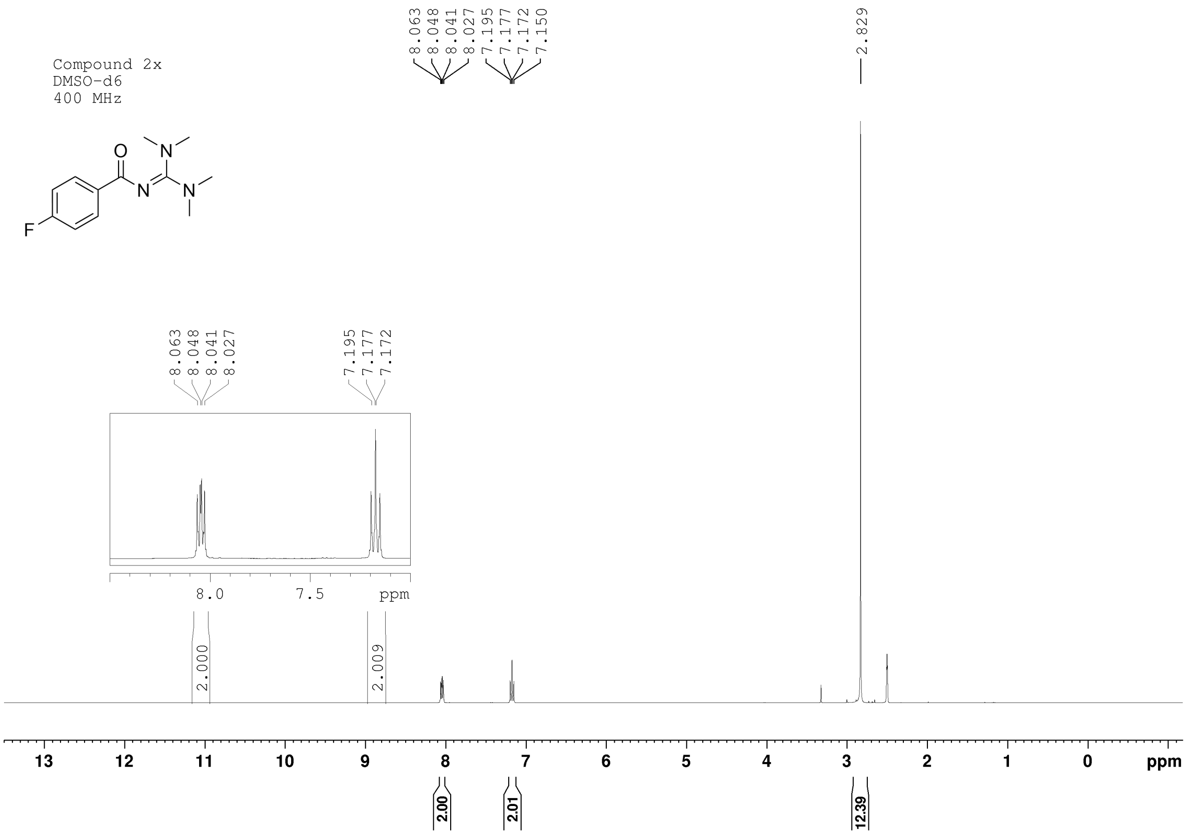

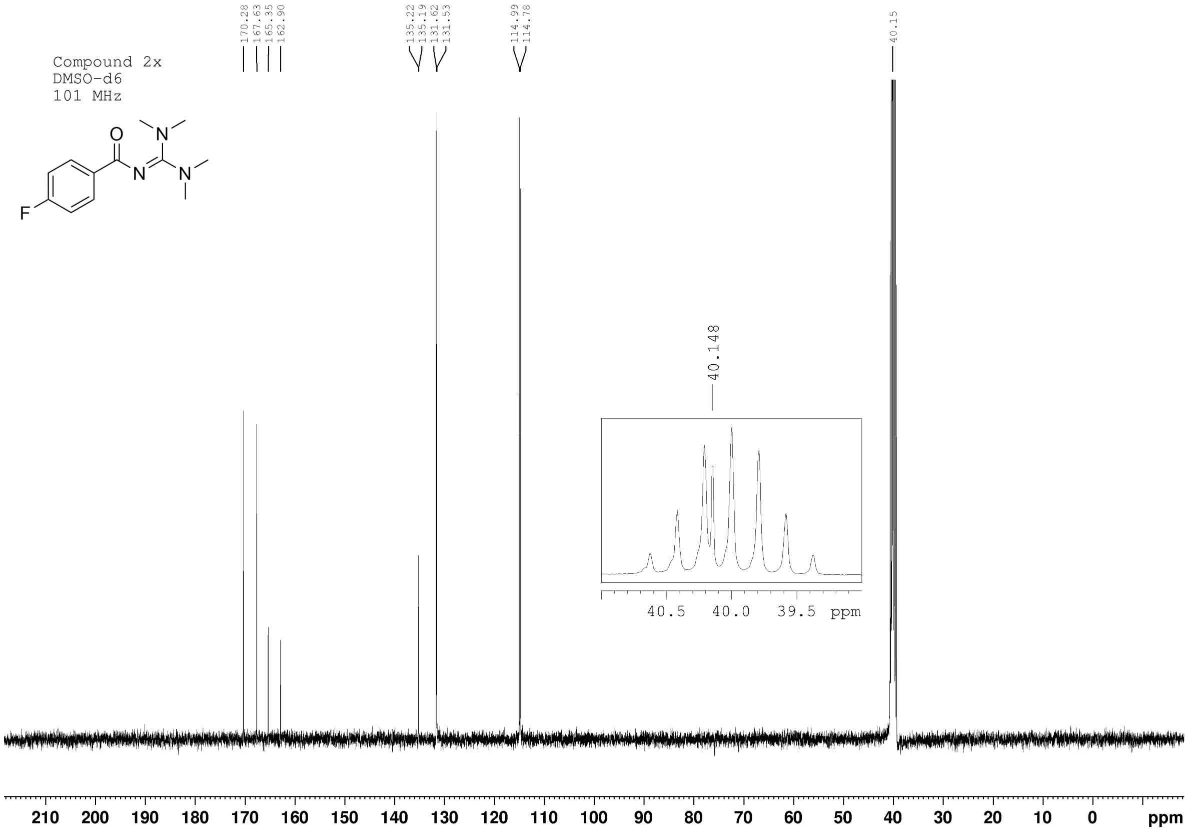
Compound 2x
DMSO-d6
101 MHz
170.28
167.63
165.35
162.90
135.22
135.19
131.62
131.53
114.99
114.78
40.15
40.148
O
N
F
ppm

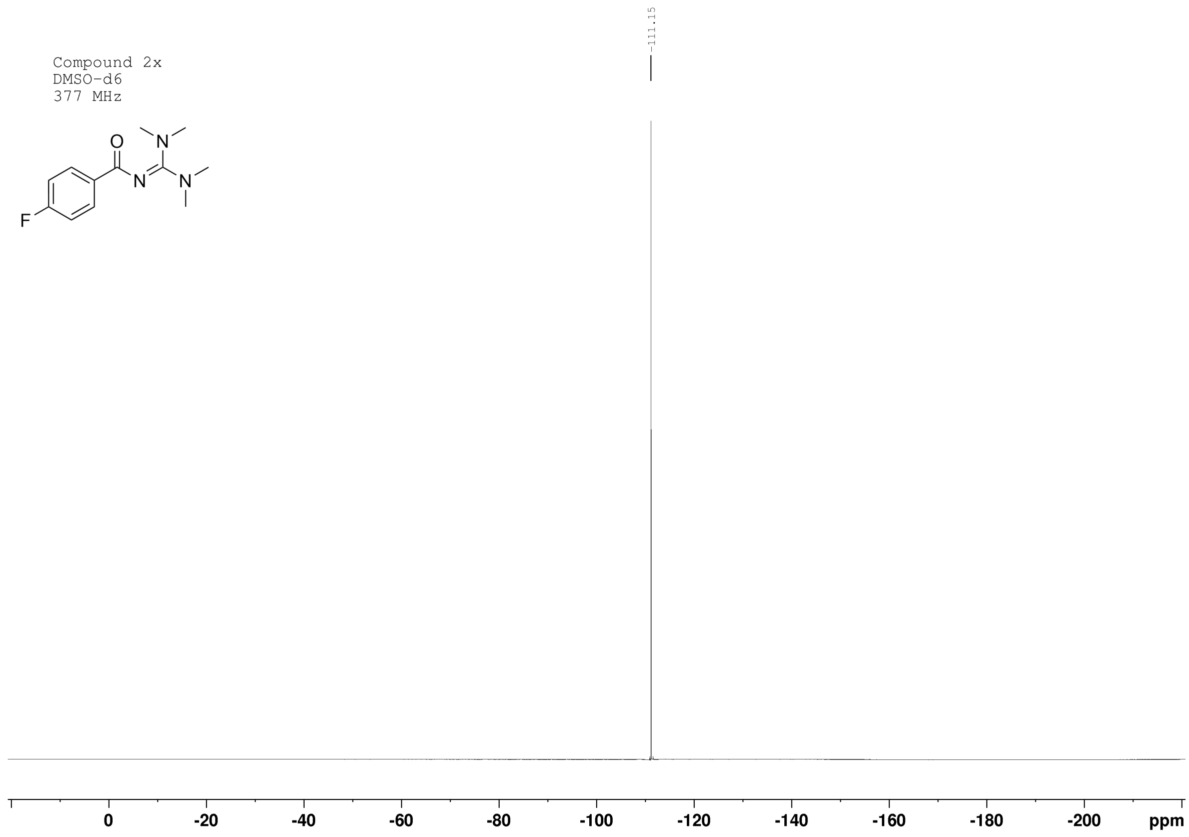
Compound 2x
DMSO-d6
377 MHz
-111.15
O
N
F
ppm

2y

1,1,3,3-Tetramethyl-2-trifluoroacetylguanidine

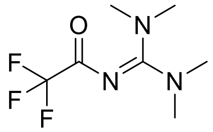

Condition B. Workup 3 with Na_2SO_4 added to aqueous phases during extractions. Silica gel chromatography was performed using 90:10 EtOAc:MeOH as solvent. Colourless oil. Yield 51 mg (12 %).

1H NMR (400 MHz, DMSO-d_6) δ [ppm]: 8.08-8.01 (m, 2H), 7.21-7.13 (m, 2H), 2.83 (s, 12H).

13C{1H} NMR (101 MHz, DMSO-d_6) δ [ppm]: 167.6, 159.6 (q, J = 32.0), 117.8 (q, J = 288.9), 40.4

19F{1H} NMR (377 MHz, CDCl3) δ [ppm]: -73.9

HRMS (ESI-TOF) m/z: [M+Na]+ Calcd for C7H12F3N3NaO 234.0825; found 234.0827

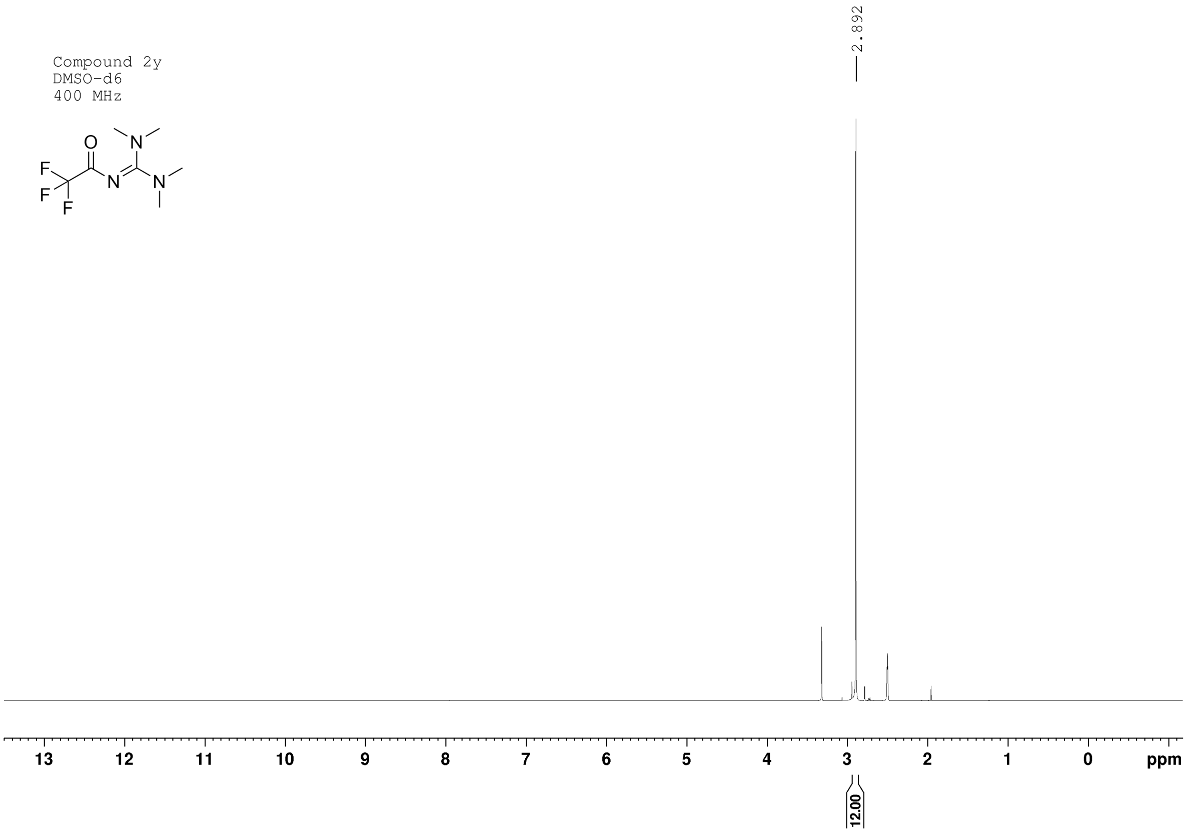

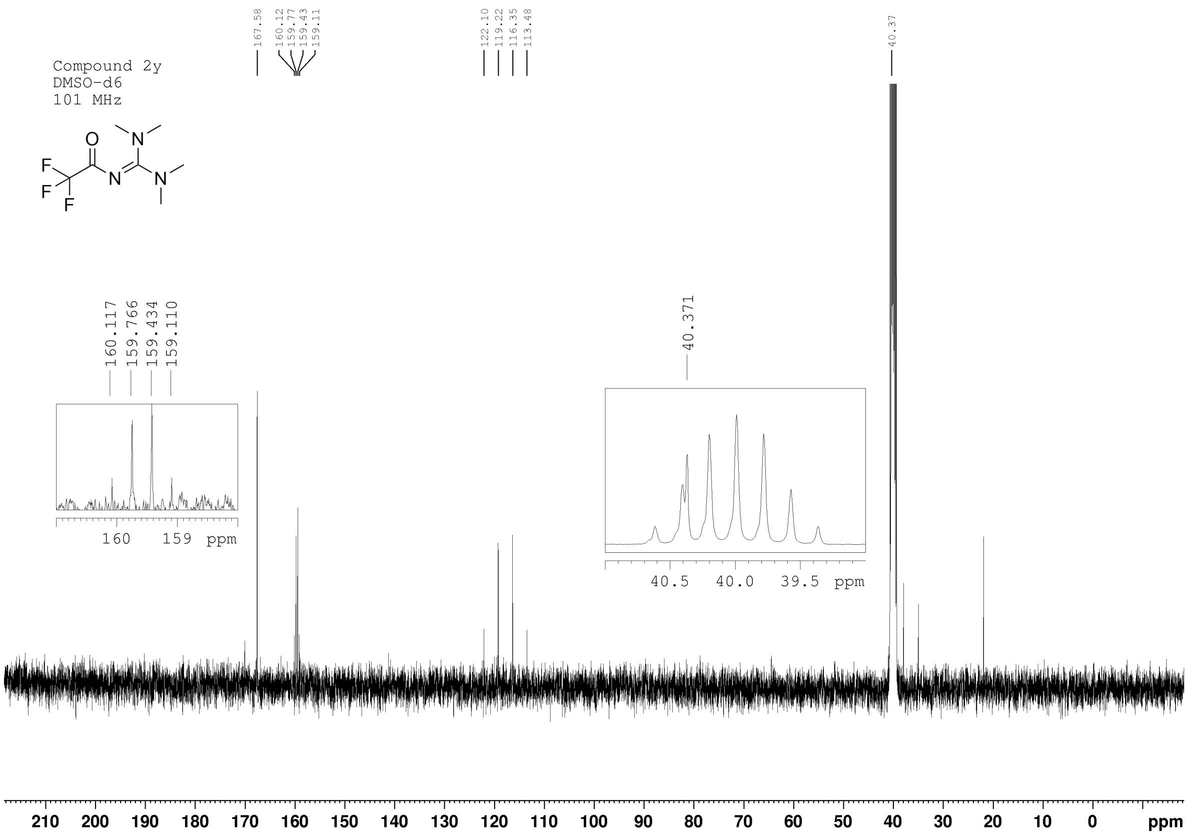
Compound 2y
DMSO-d6
101 MHz
167.58
160.12
159.77
159.43
159.11
122.10
119.22
116.35
113.48
40.37
160.117
159.766
159.434
159.110
160
159 ppm
40.371
40.5
40.0
39.5 ppm
210 200 190 180 170 160 150 140 130 120 110 100 90 80 70 60 50 40 30 20 10 0 ppm

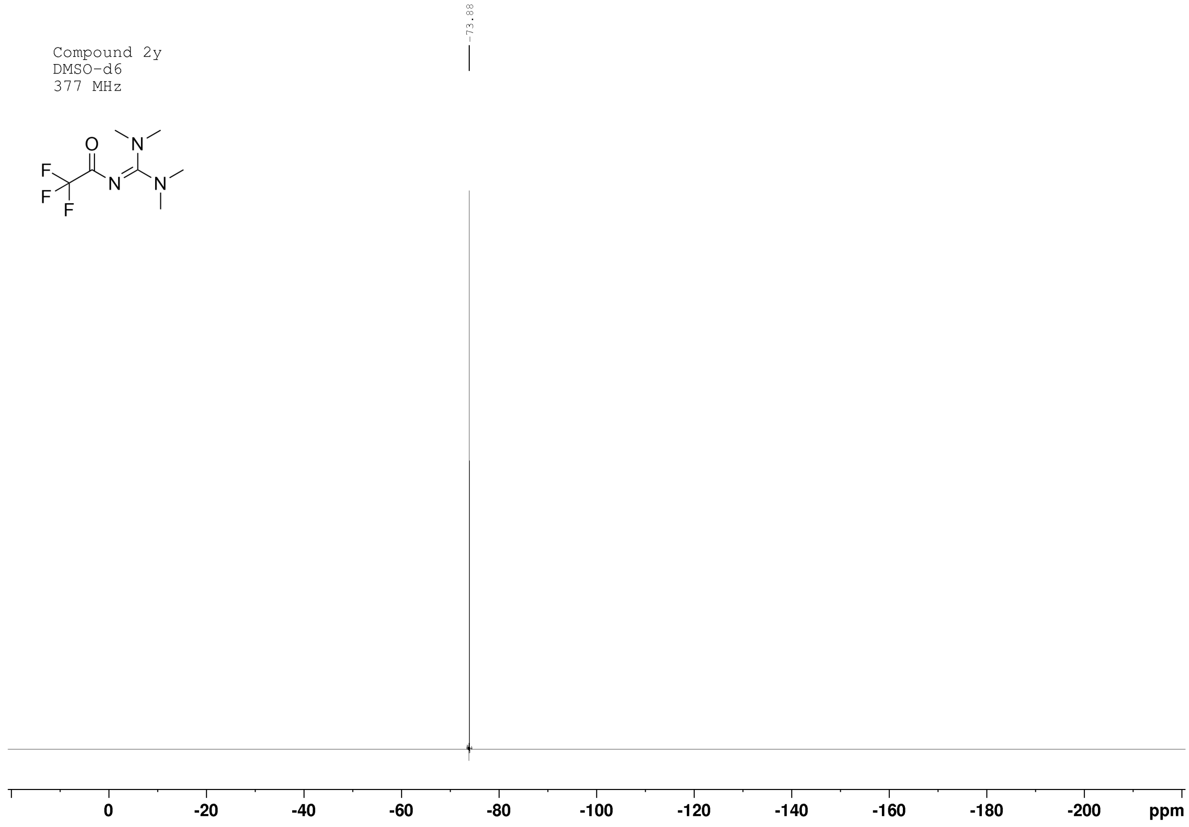
Compound 2y
DMSO-d6
377 MHz
-73.88
0 -20 -40 -60 -80 -100 -120 -140 -160 -180 -200 ppm

2z

1,1,3,3-Tetramethyl-2-(tert-butyloxycarbonyl)guanidine

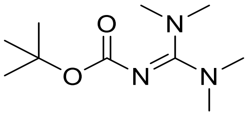

Condition B. Workup 3. Silica gel chromatography was performed using 90:10 EtOAc:MeOH as solvent. White solid. Yield 369 mg (86 %).

1H NMR (400 MHz, DMSO-d_6) δ [ppm]: 2.75 (s, 12H), 1.37 (s, 9H)

13C{1H} NMR (101 MHz, DMSO-d_6) δ [ppm]: 164.7, 159.5, 76.3, 39.7, 28.6

HRMS (ESI-TOF) m/z: [M+H]+ Calcd for $C_{10}H_{22}N_3O_2$ 216.1707; found 216.1710

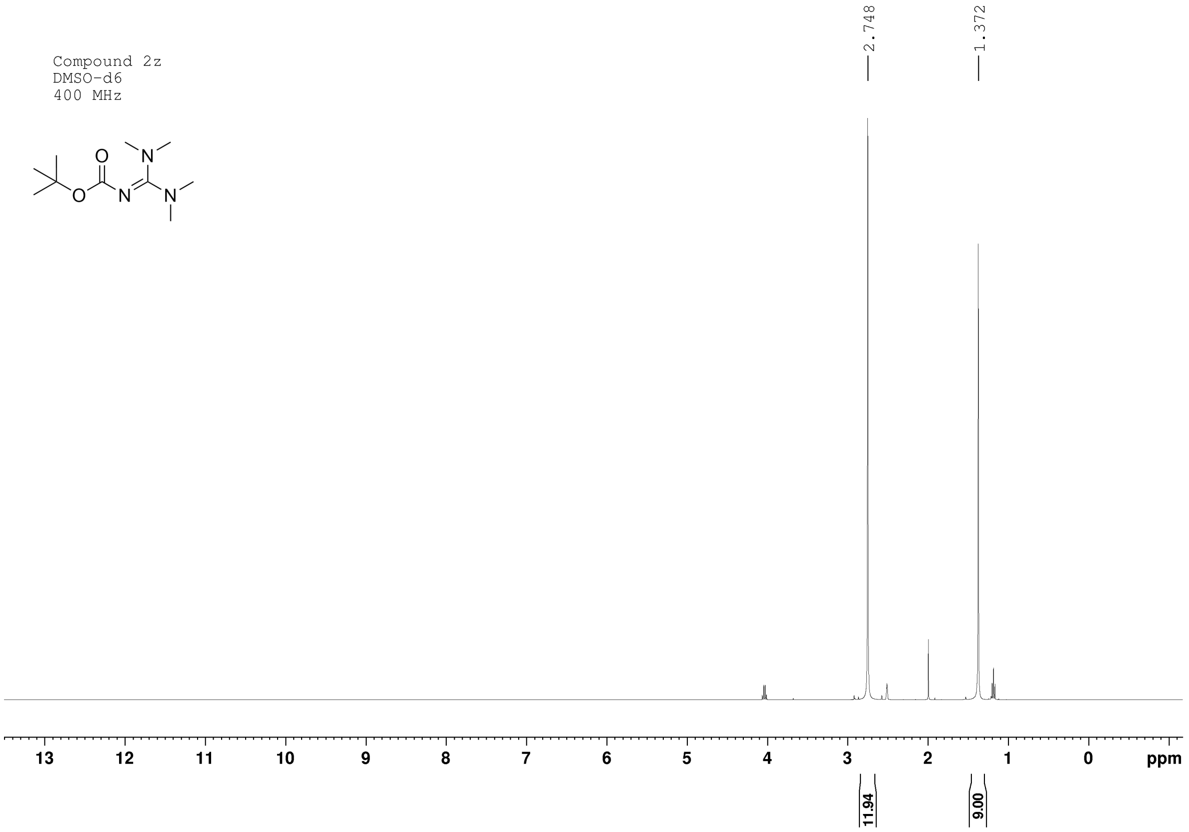

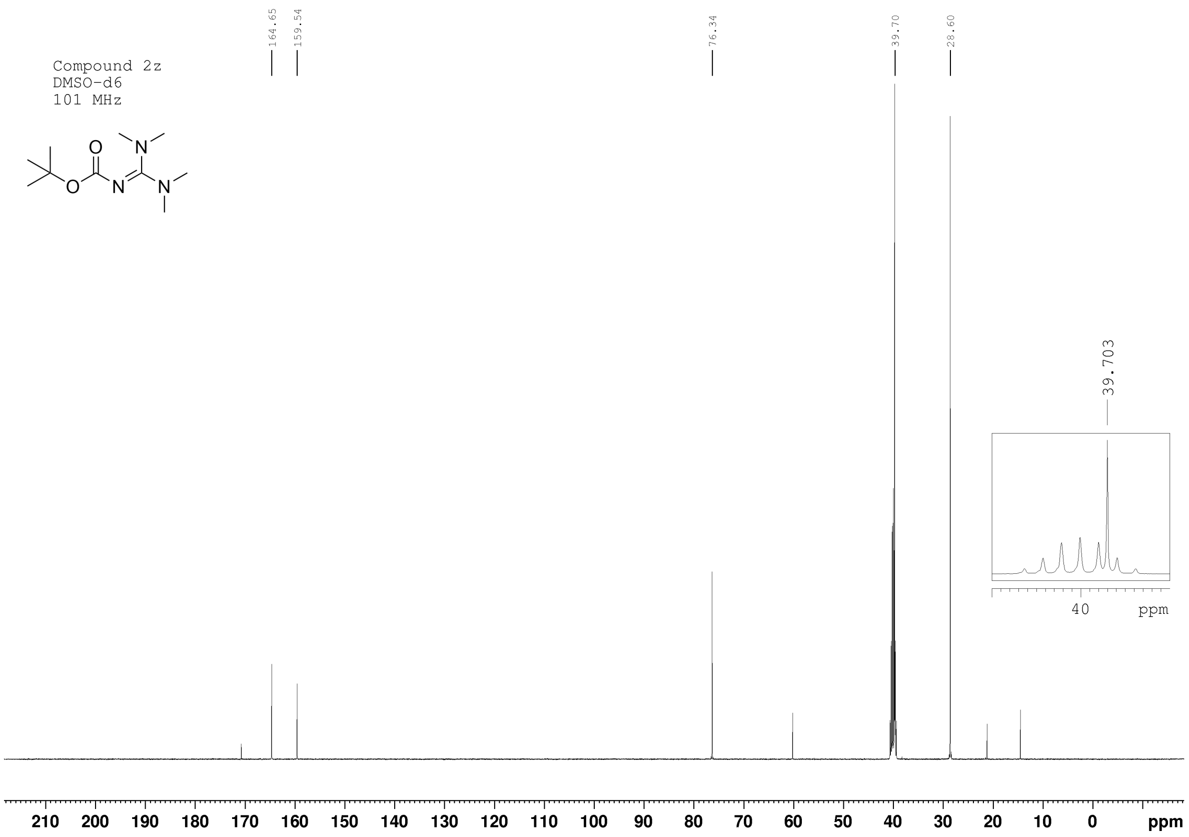

2aa

1,1,3,3-Tetramethyl-2-(benzyloxycarbonyl)guanidine

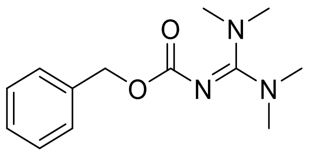

Condition B. Workup 3. Silica gel chromatography was performed using 90:10 EtOAc:MeOH as solvent. White solid. Yield 372 mg (75 %).

1H NMR (400 MHz, DMSO-d_6) δ [ppm]: 7.39-7.22 (m, 5H), 4.98 (s, 2H), 2.76 (s, 12H).

13C{1H} NMR (101 MHz, DMSO-d_6) δ [ppm]: 165.7, 159.6, 138.6, 128.7, 128.1, 127.9, 66.1, 39.8

HRMS (ESI-TOF) m/z: [M+H]+ Calcd for C13H20N3O2 250.1550; found 250.1554

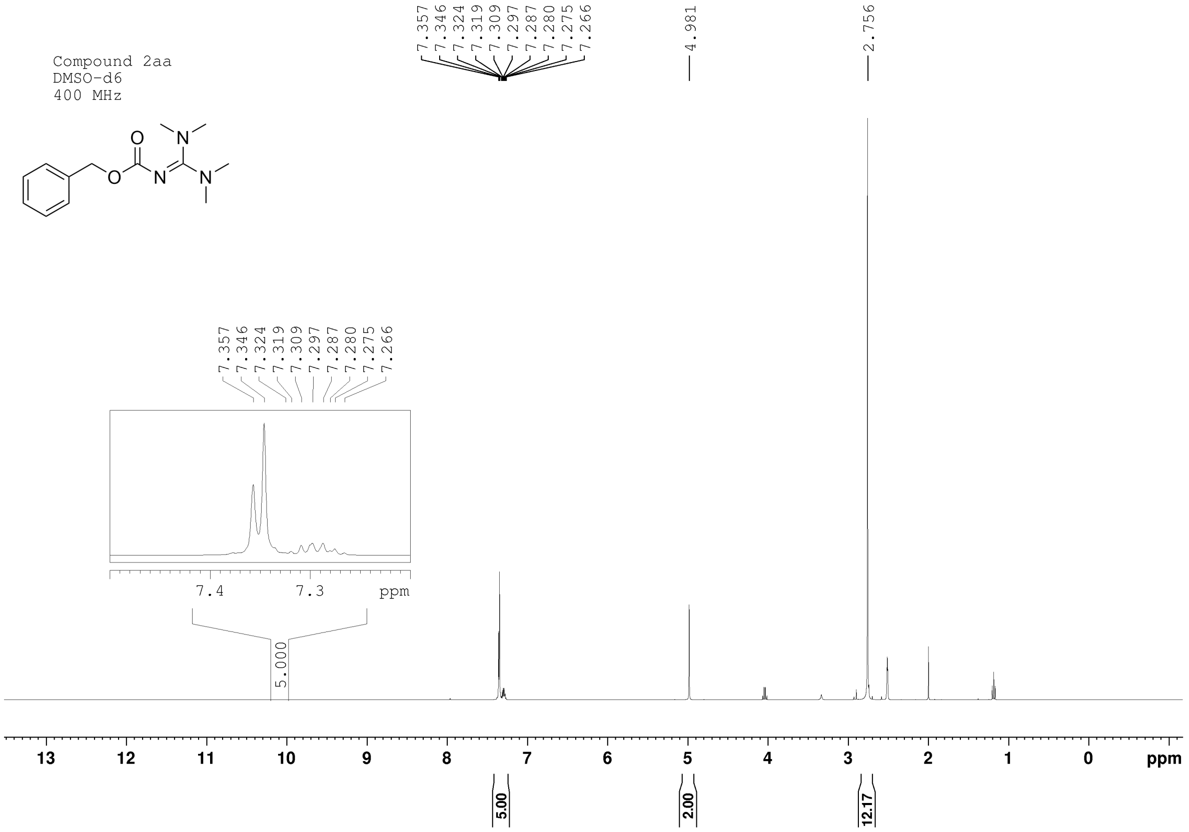
Compound 2aa
DMSO-d6
400 MHz
7.357
7.346
7.324
7.319
7.309
7.297
7.287
7.280
7.275
7.266
4.981
2.756
5.000
5.00
2.00
12.17
ppm

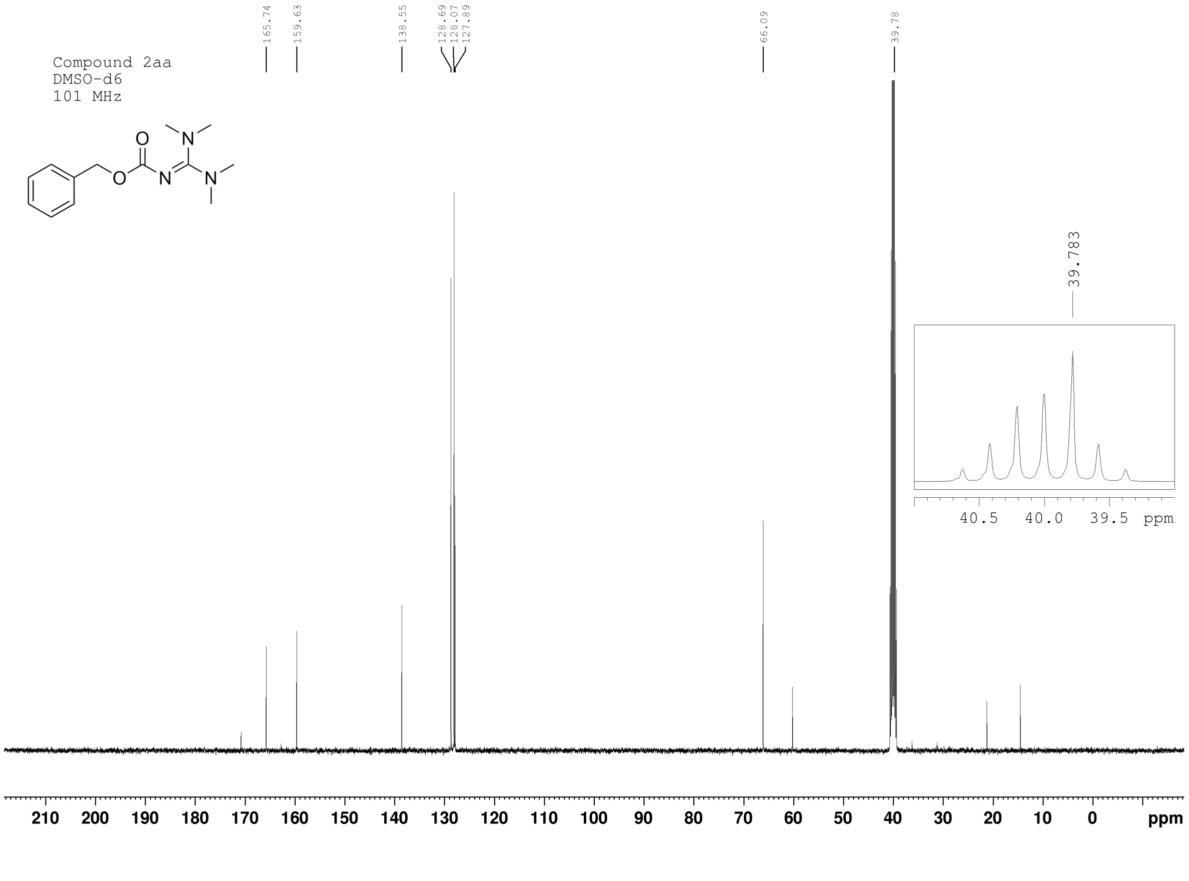
Compound 2aa
DMSO-d6
101 MHz
165.74
159.63
138.55
128.69
128.07
127.89
66.09
39.78
39.783
ppm

2ab

1,1,3,3-Tetramethyl-2-(4-methylbenzenesulfonamido)guanidine

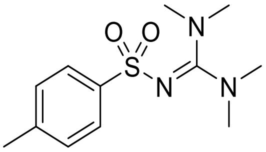

Condition A with ACN. Workup 1. Beige solid. Yield 498 mg (93 %).

1H NMR (400 MHz, DMSO-d_6) δ [ppm]: 7.63 (d, J = 8.2, 2H), 7.28 (d, J = 8.0, 2H), 2.82 (s, 12H), 2.34 (s, 3H).

13C{1H} NMR (101 MHz, DMSO-d_6) δ [ppm]: 161.5, 144.1, 140.9, 129.5, 125.6, 40.4, 21.3

HRMS (ESI-TOF) m/z: [M+Na]+ Calcd for $C_{12}H_{19}N_3NaO_2S$ 292.1090; found 292.1096

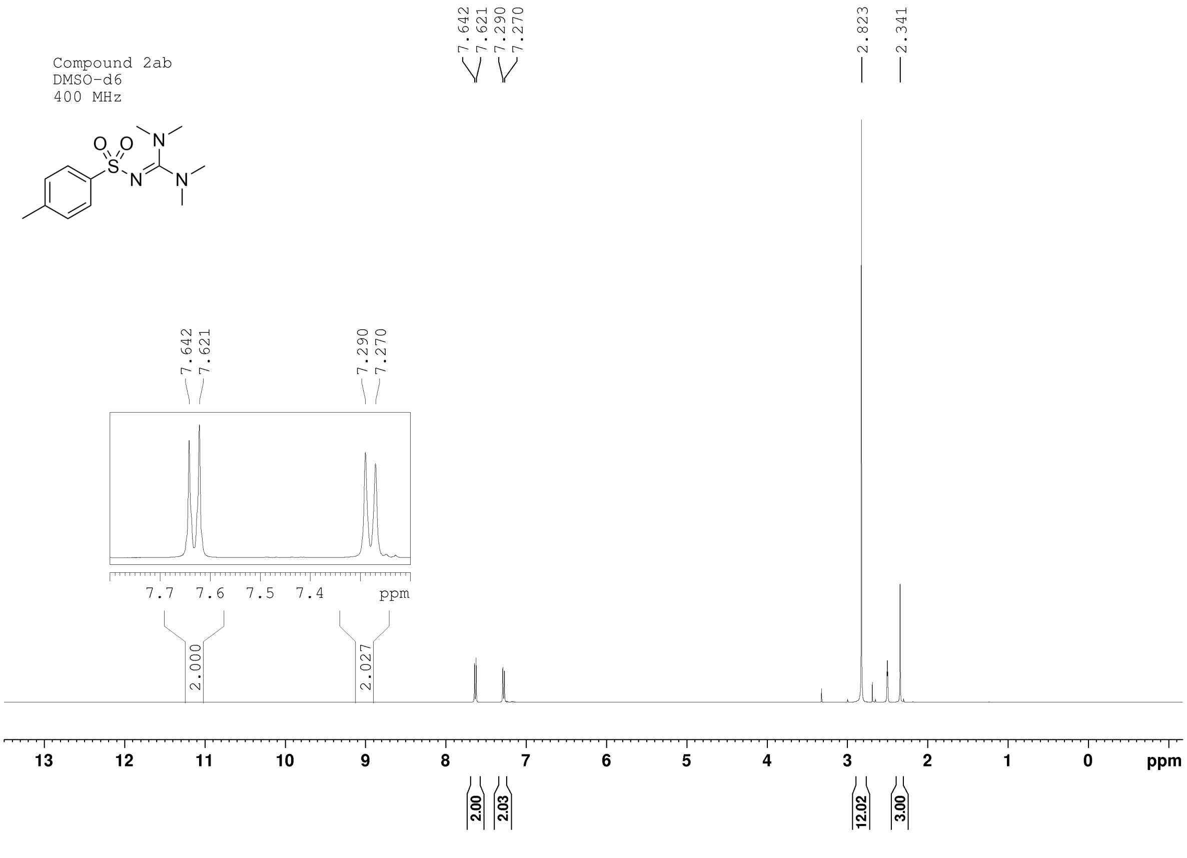

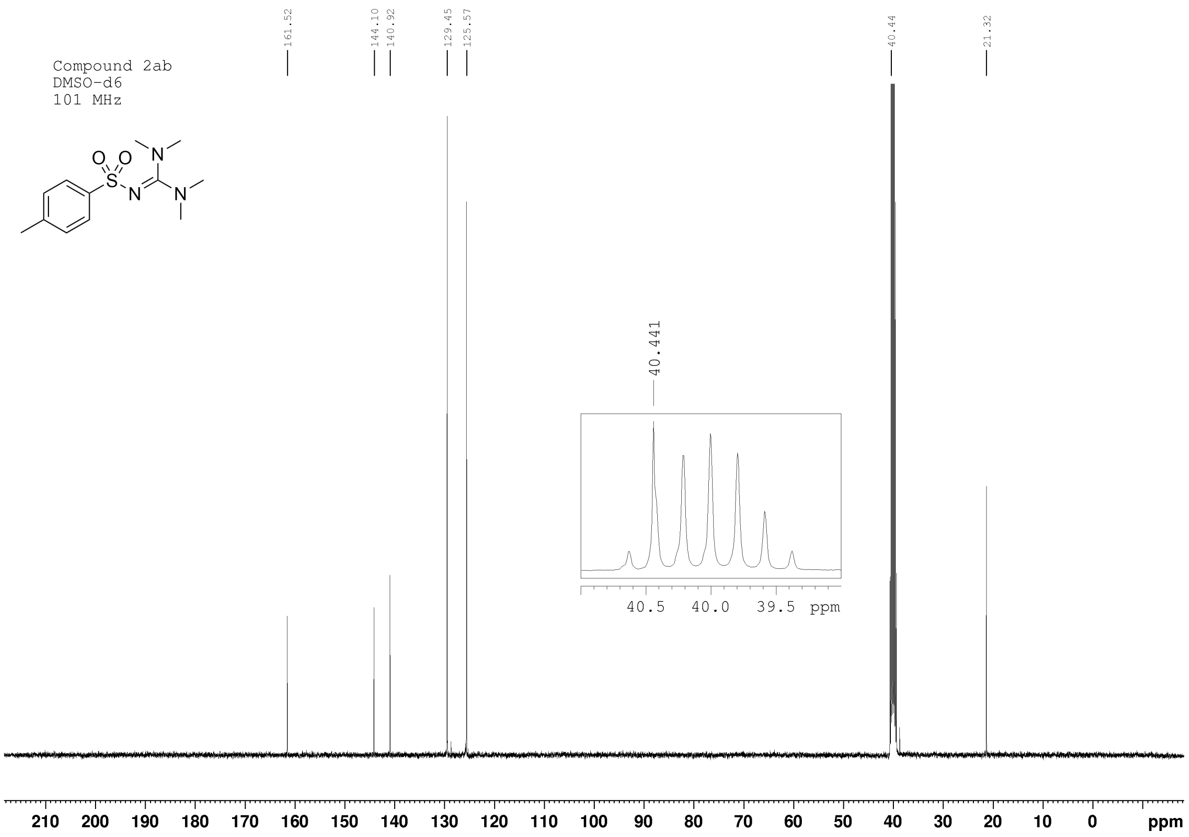

2ac

1,1,3,3-Tetramethyl-2-(4-nitrobenzenesulfonamido)guanidine

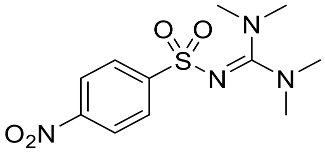

Condition A with ACN. Workup 1. White solid. Yield 565 mg (94 %).

1H NMR (400 MHz, DMSO-d_6) δ [ppm]: 8.40-8.29 (m, 2H), 8.06-7.96 (m, 2H), 2.86 (s, 12H).

13C{1H} NMR (101 MHz, DMSO-d_6) δ [ppm]: 161.5, 152.0, 148.9, 127.1, 124.7, 40.6

HRMS (ESI-TOF) m/z: [M+H]+ Calcd for C11H16N4NaO4S 323.0784; found 323.0787

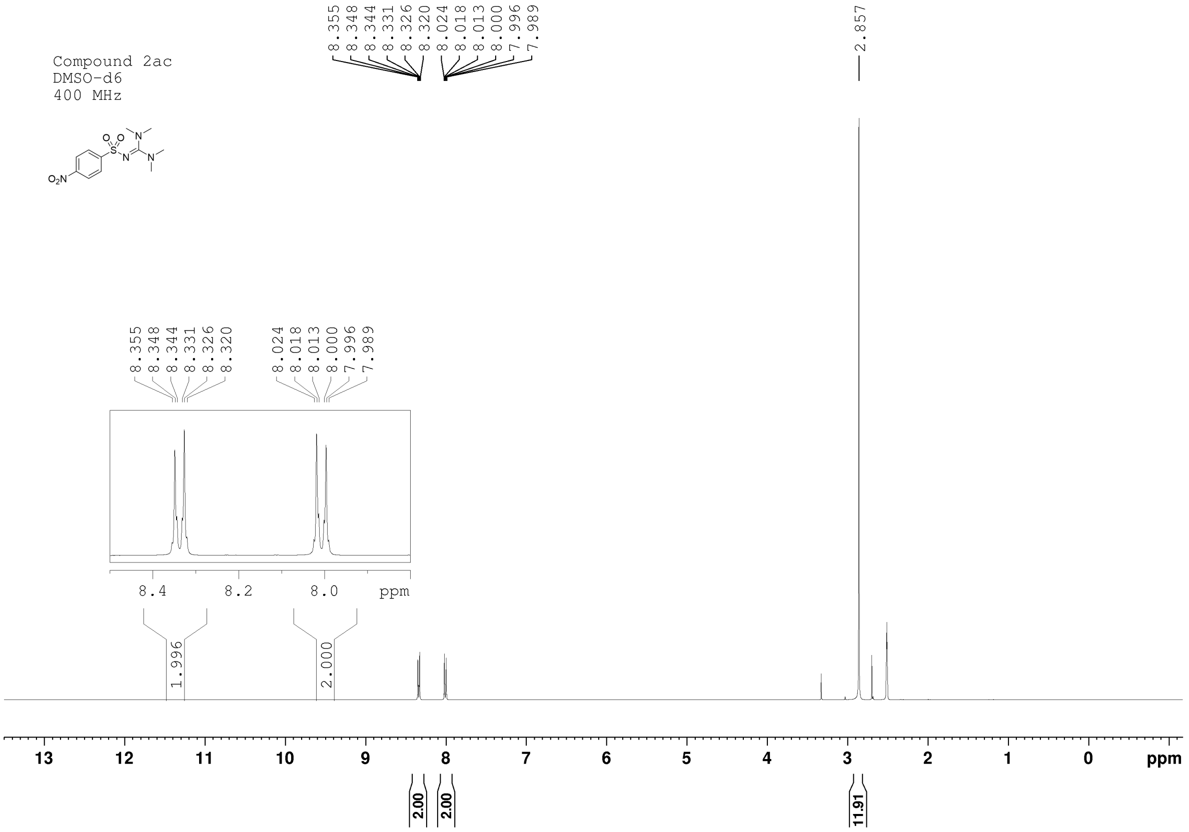
Compound 2ac
DMSO-d6
400 MHz
8.355 8.348 8.344 8.331 8.326 8.320 8.024 8.018 8.013 8.000 7.996 7.989
2.857
1.996
2.000
2.00
2.00
11.91
ppm

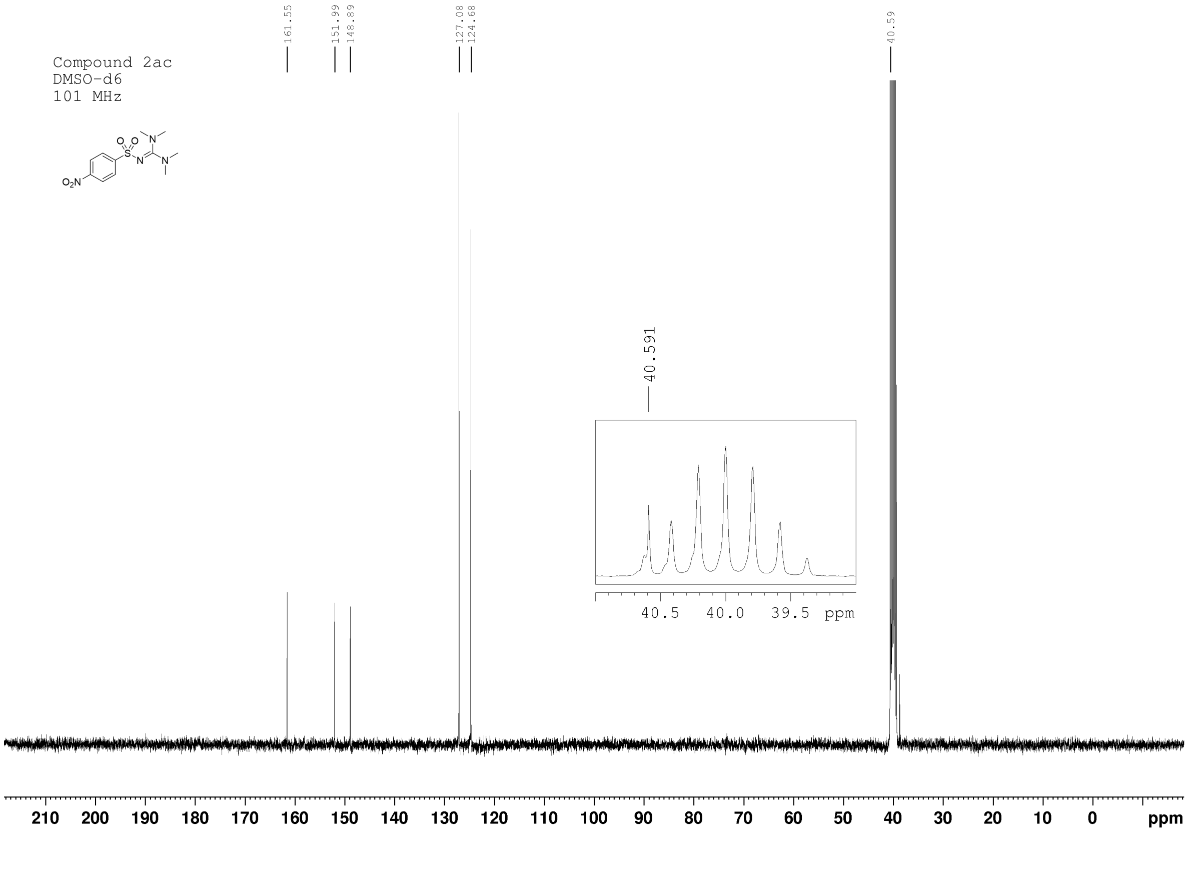
Compound 2ac
DMSO-d6
101 MHz
161.55 151.99 148.89 127.08 124.68 40.59
40.591
ppm

2af

2-Dibenzoazepine-5-carboxamide-1,1,3,3-tetramethyl-guanidine

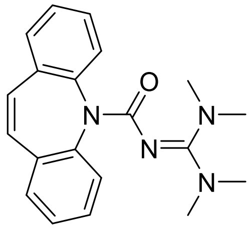

Condition B with DMF. Workup 3. Silica gel chromatography was performed using 90:10 EtOAc:MeOH as solvent. White solid. Yield 488 mg (73 %).

1H NMR (400 MHz, DMSO-d_6) δ [ppm]: 7.43-7.30 (m, 6H), 7.29-7.19 (m, 2H), 6.92 (s, 2H), 2.64 (s, 12H).

13C{1H} NMR (101 MHz, DMSO-d6) δ [ppm]: 164.3, 159.9, 130.8, 130.4, 129.1, 128.8, 126.5, 49.1, 39.6

HRMS (ESI-TOF) m/z: [M+H]+ Calcd for $C_{20}H_{23}N_4O$ 335.1866; found 335.1861

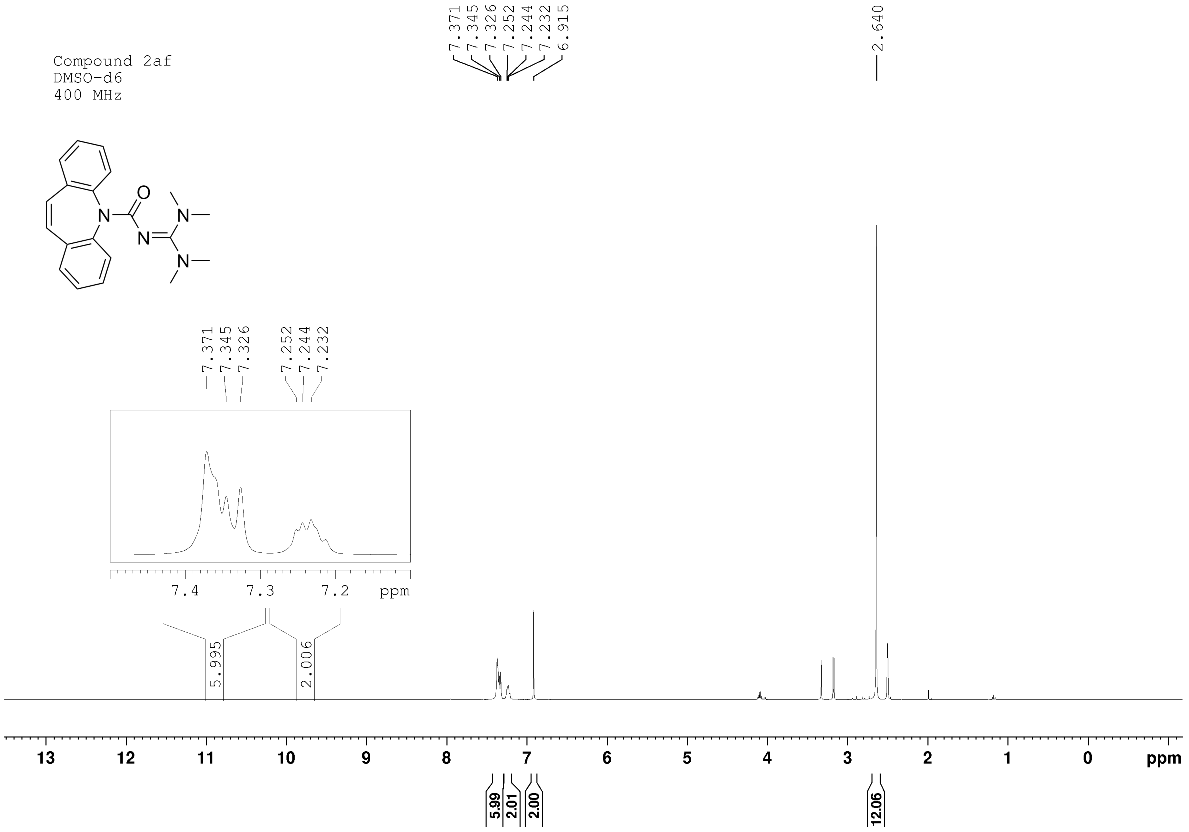

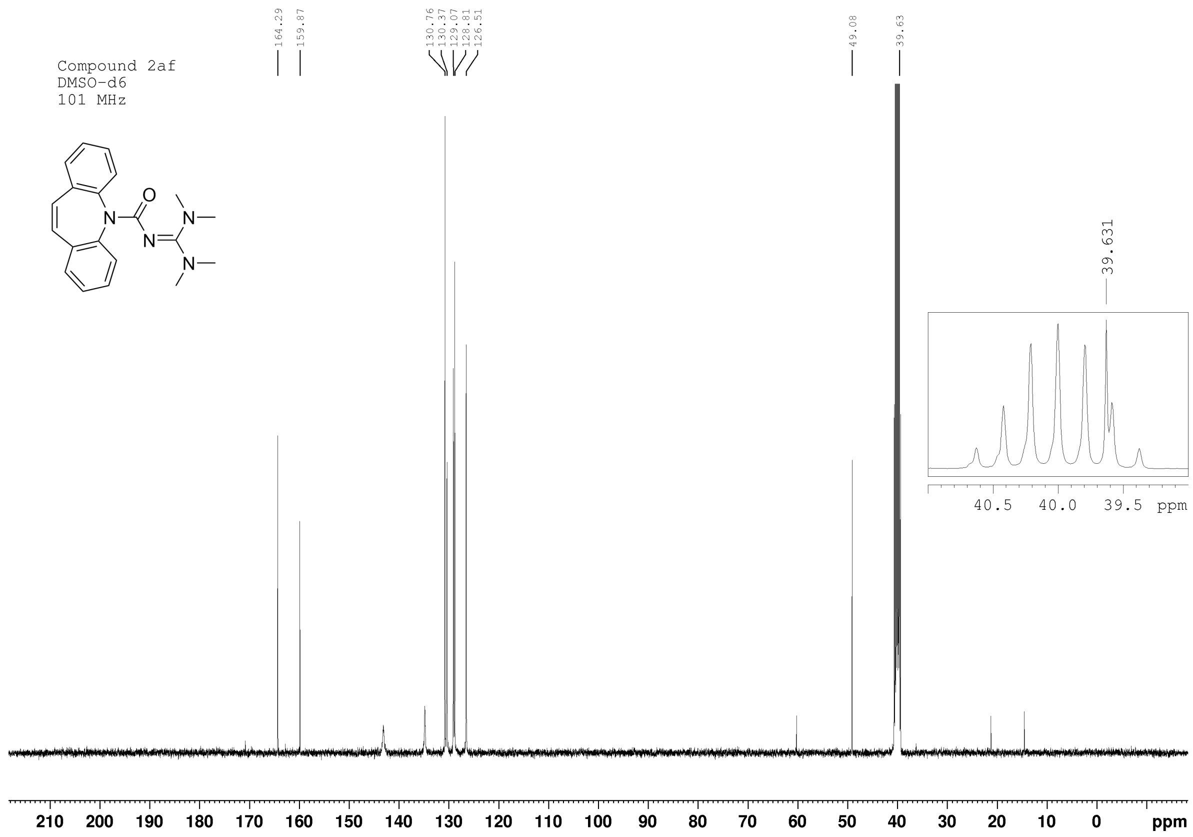

2ag

1,1,3,3-Tetramethyl-2-(*N,N*-diphenylthiourea)guanidine

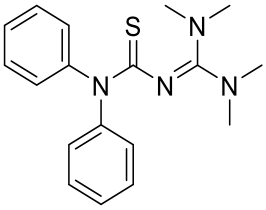

Condition B with DMF. Workup 3. Silica gel chromatography was performed using 90:10 EtOAc:MeOH as solvent. Beige solid. Yield 509 mg (78 %).

1H NMR (400 MHz, $CDCl_3$) δ [ppm]: 7.40-7.35 (m, 4H), 7.34-7.27 (m, 4H), 7.20-7.14 (m, 2H), 2.96 (s, 12H).

13C{1H} NMR (101 MHz, CDCl3) δ [ppm]: 184.8, 168.1, 146.8, 128.7, 128.7 (d, J = 4.5), 126.0, 40.4

HRMS (ESI-TOF) m/z: [M+Na]+ Calcd for C18H22N4SNa 349.1457; found 349.1450

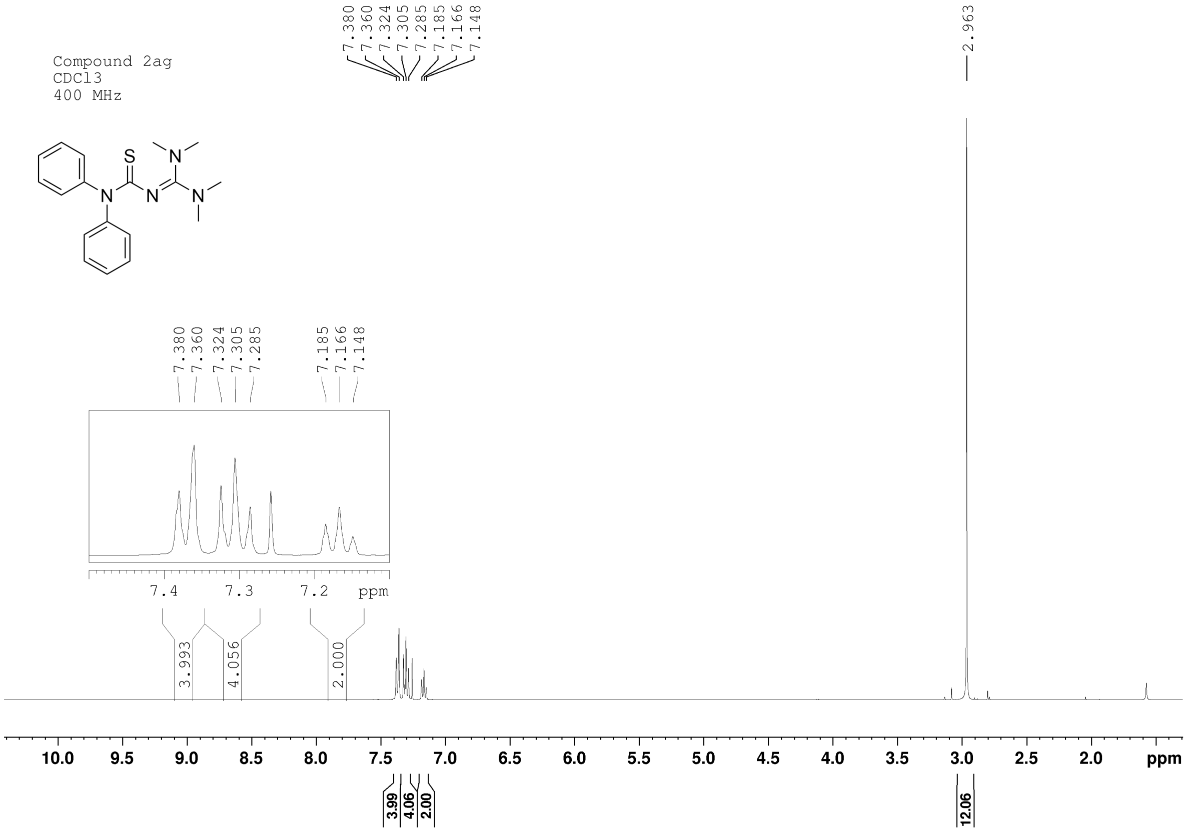
Compound 2ag
CDCl3
400 MHz
7.380
7.360
7.324
7.305
7.285
7.185
7.166
7.148
2.963
3.993
4.056
2.000
3.99
4.06
2.00
12.06
ppm

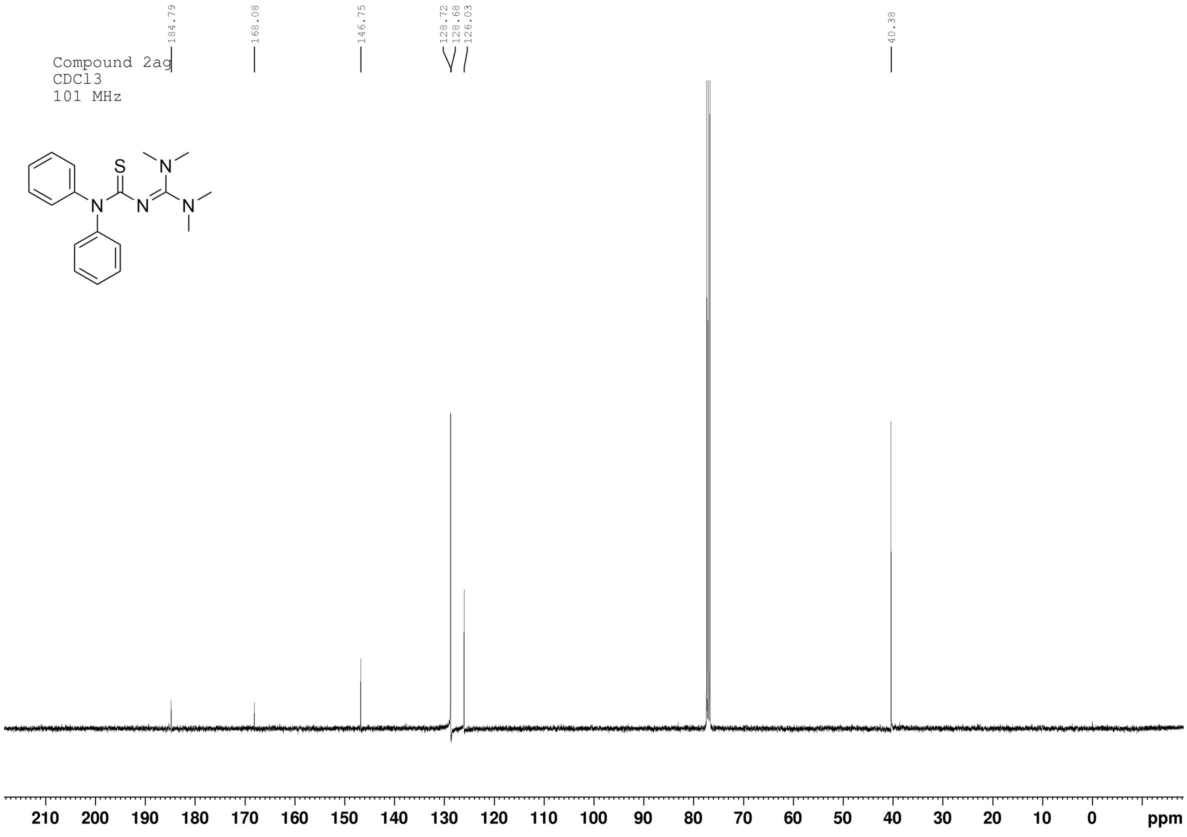
Compound 2ag
CDCl3
101 MHz
184.79
168.08
146.75
128.72
128.68
126.03
40.38
ppm

2ah

1,1,3,3-Tetramethyl-2-(5-bromoquinoxalin-6-yl)guanidine

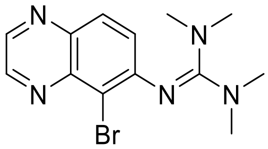

Condition B. Workup 2. Orange oil. Yield 410 mg (64 %).

1H NMR (400 MHz, DMSO-d_6) δ [ppm]: 8.84 (d, J = 1.9, 1H), 8.67 (d, J = 1.9, 1H), 7.83 (d, J = 8.9, 1H), 7.23 (d, J = 8.9, 1H), 2.70 (s, 12H).

13C{1H} NMR (101 MHz, DMSO-d_6) δ [ppm]: 160.6, 153.5, 145.8, 142.2, 139.2, 128.9, 128.6, 112.1, 39.7

HRMS (ESI-TOF) m/z: [M+H]+ Calcd for $C_{13}H_{17}BrN_5$ 322.0662; found 322.0669

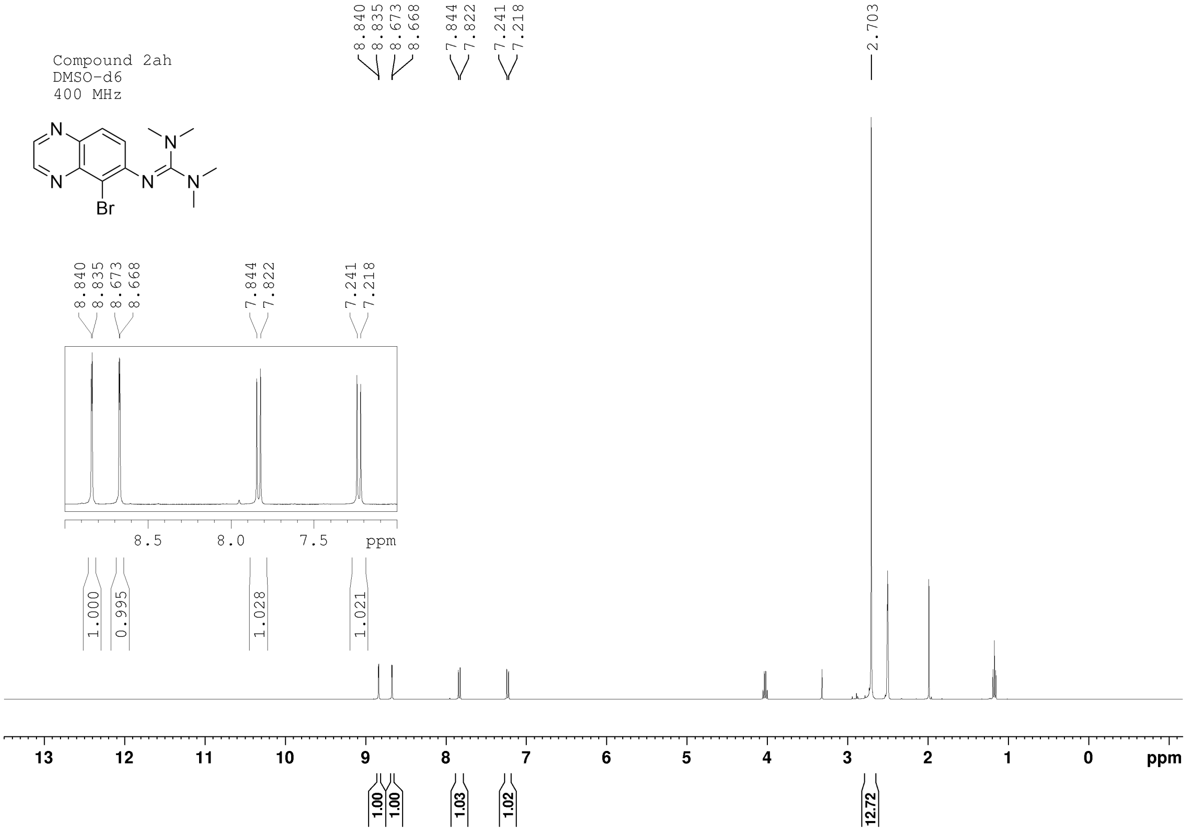

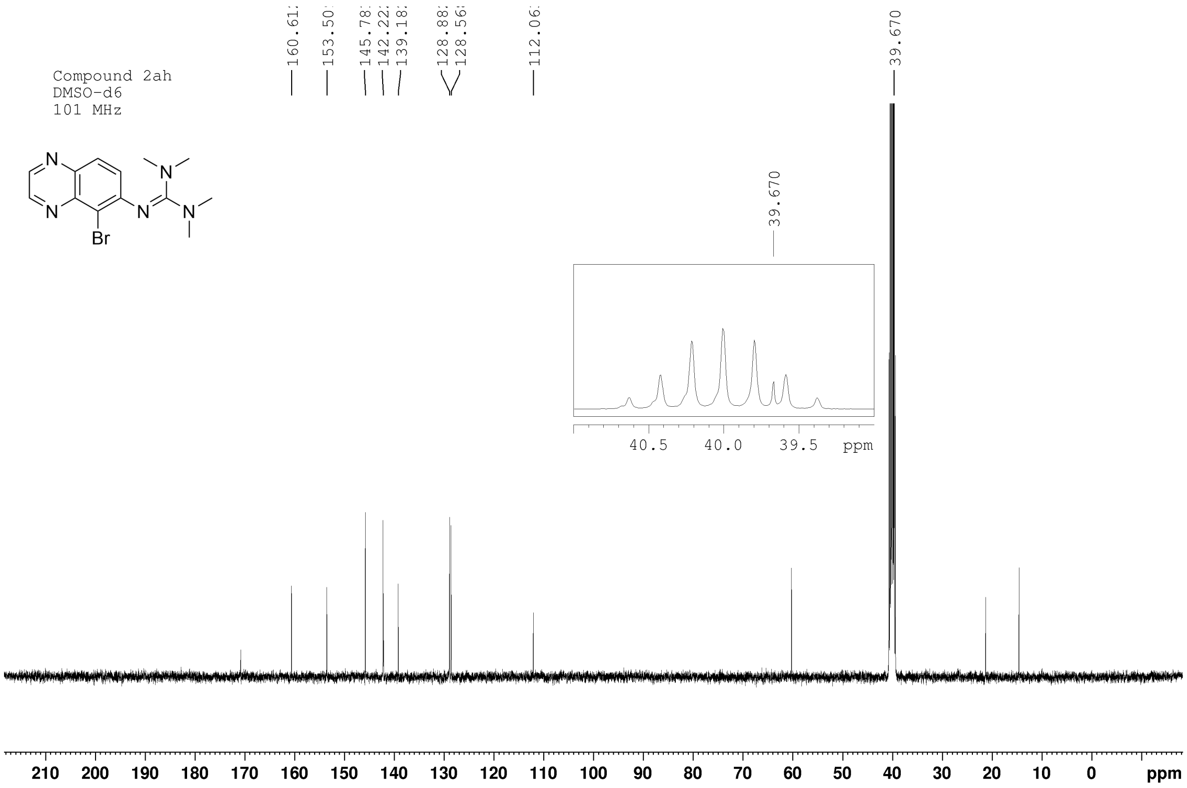
Compound 2ah
DMSO-d6
101 MHz
Br
N
160.61
153.50
145.78
142.22
139.18
128.88
128.56
112.06
39.670
40.5
40.0
39.5
ppm
210
200
190
180
170
160
150
140
130
120
110
100
90
80
70
60
50
40
30
20
10
0
ppm

2ai

N-(bis(dimethylamino)methylene)-4-(5-(p-tolyl)-3-(trifluoromethyl)-1H-pyrazol-1-yl)benzenesulfonamide

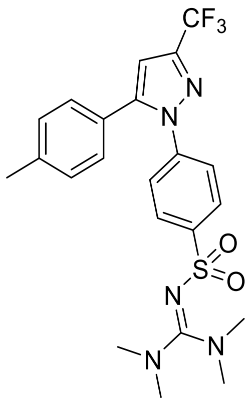

Condition A with ACN. Workup 1. Yield 800 mg (83 %), white solid.

1H NMR (400 MHz, DMSO-d_6) δ [ppm]: 7.84-7.77 (m, 2H), 7.50-7.41 (m, 2H), 7.23-7.13 (m, 5H), 2.84 (s, 12H), 2.31 (s, 3H).

13C{1H} NMR (101 MHz, DMSO-d_6) δ [ppm]: 161.5, 146.7, 145.7, 142.5 (q, J = 37.8), 140.6, 139.5, 129.8, 129.4, 129.0 (q, J = 70.5), 126.7, 126.4, 125.8, 121.8 (q, J = 267.1), 106.3, 40.5, 38.7

19F{1H} NMR (377 MHz, CDCl3) δ [ppm]: -60.8

HRMS (ESI-TOF) m/z: [M+H]+ Calcd for C22H25F3N5O2S 480.1676; found 480.1665

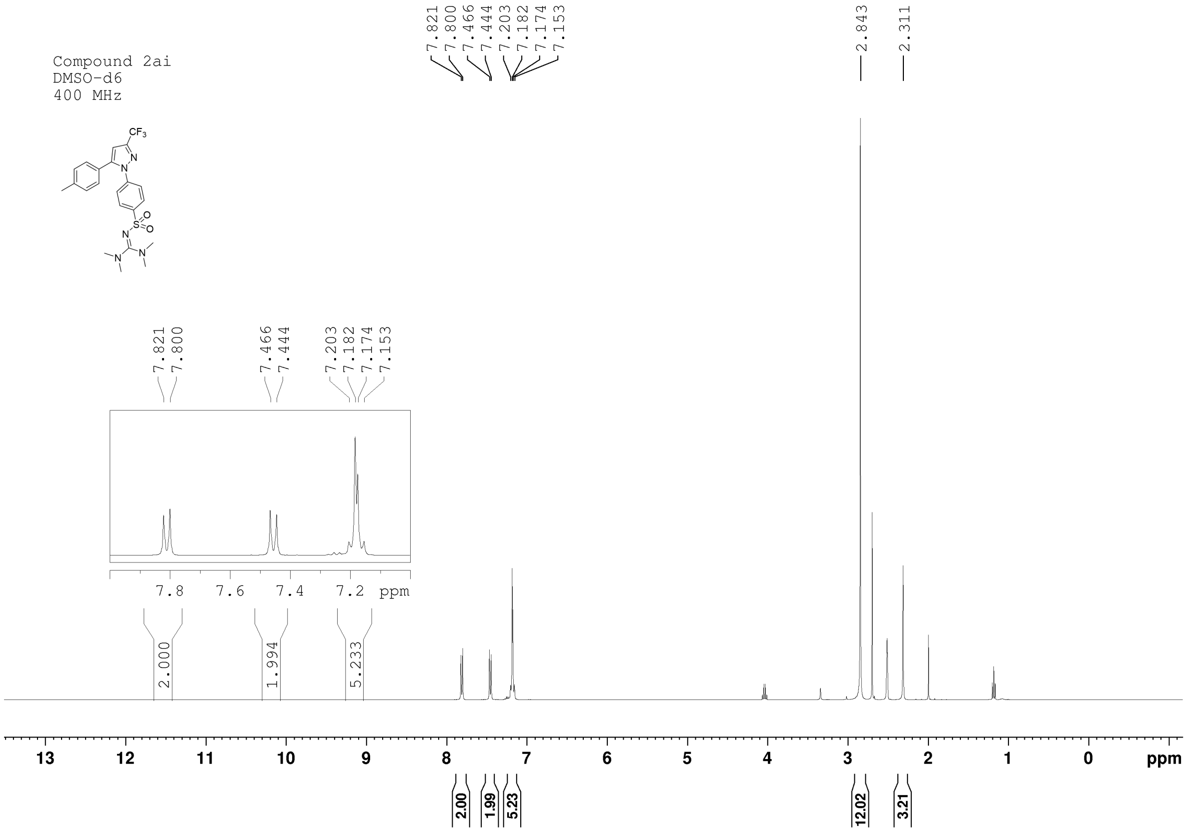
Compound 2ai
DMSO-d6
400 MHz
CF3
7.821
7.800
7.466
7.444
7.203
7.182
7.174
7.153
2.843
2.311
2.000
1.994
5.233
2.00
1.99
5.23
12.02
3.21
ppm

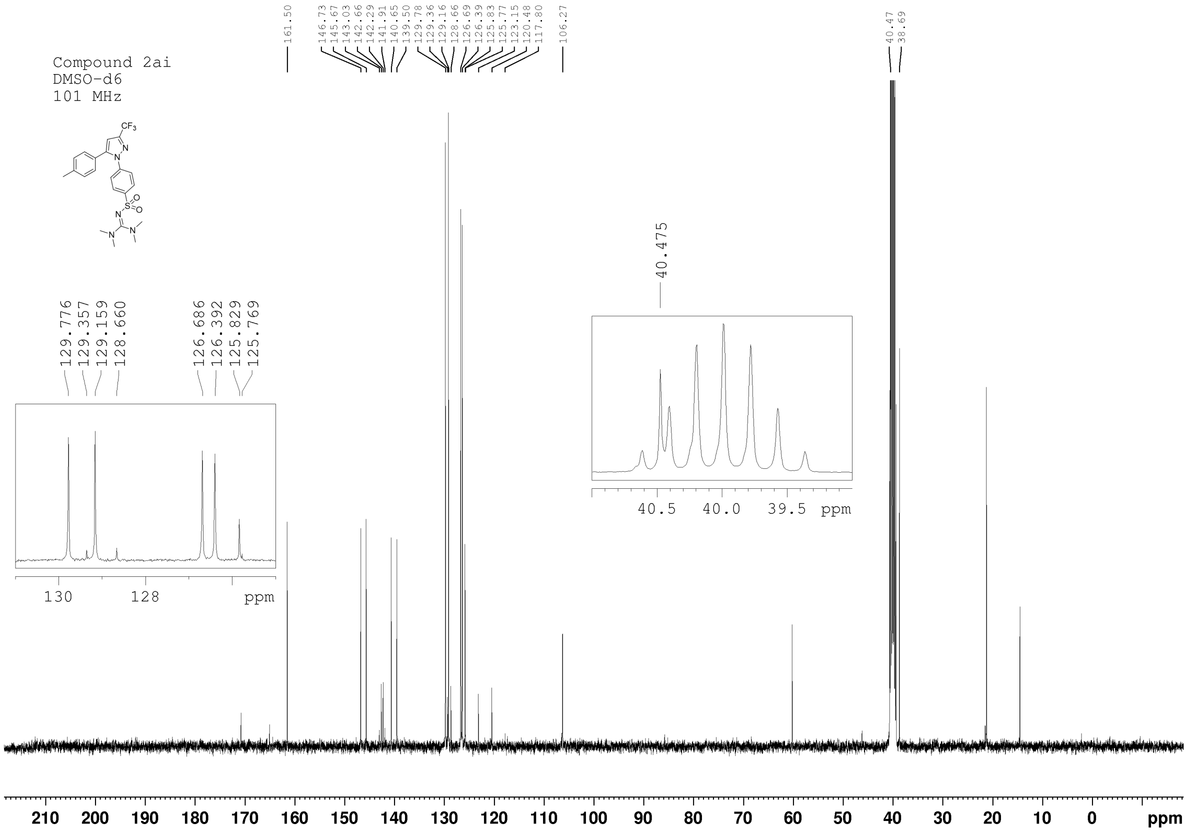
Compound 2ai
DMSO-d6
101 MHz
CF3
161.50
146.73
145.67
143.03
142.66
142.29
141.91
140.65
139.50
129.78
129.36
129.16
128.66
126.69
126.39
125.83
125.77
123.15
120.48
117.80
106.27
40.47
38.69
129.776
129.357
129.159
128.660
126.686
126.392
125.829
125.769
40.475
ppm

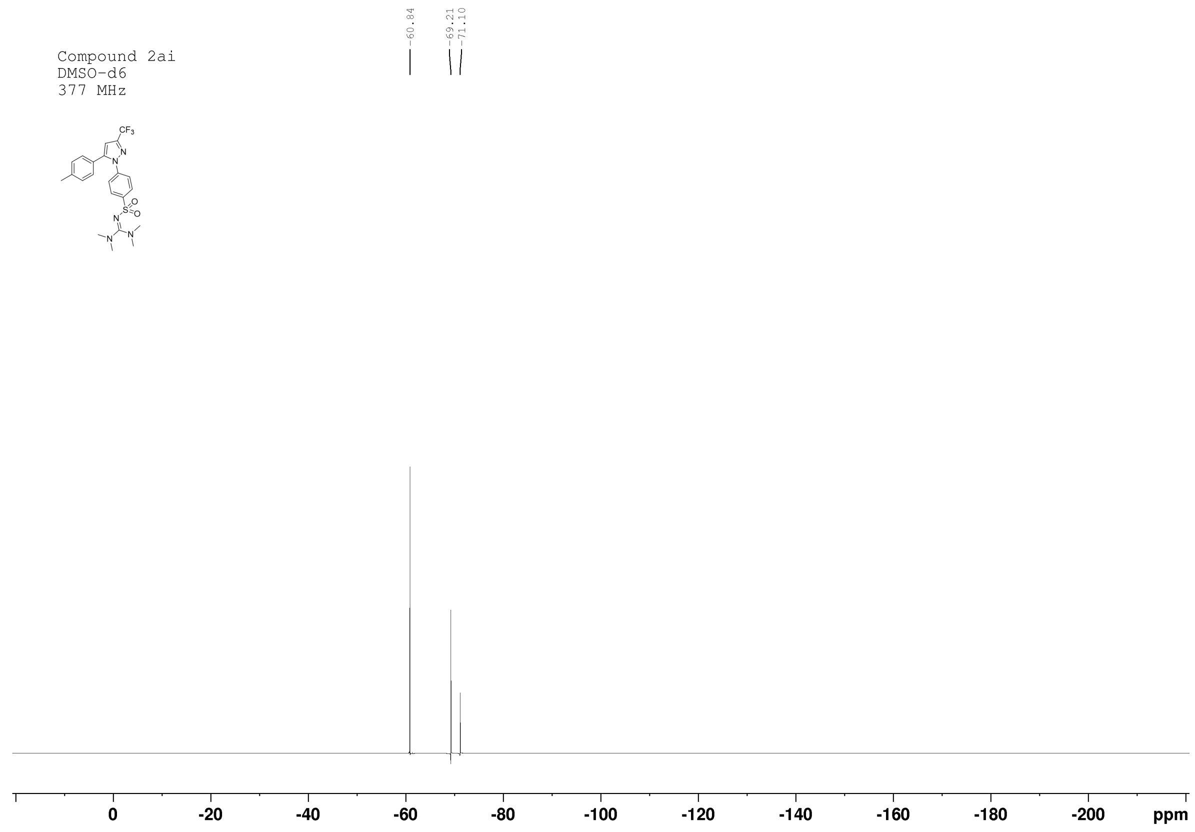

2aj

(S)-2-((3-(3-fluoro-4-morpholinophenyl)-2-oxooxazolidin-5-yl)methyl)-1,1,3,3-tetramethylguanidine

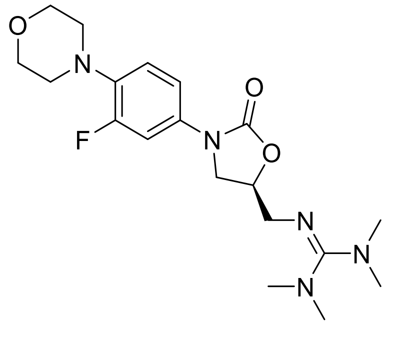

Condition A with ACN. Workup 3. Silica gel chromatography was performed using 90:10 EtOAc:MeOH as solvent. Yield 298 mg (28 %) as PF6 salt.

1H NMR (400 MHz, DMSO-d_6) δ [ppm]: 7.87 (bs, 1H), 7.51 (dd, J = 15.0, 2.5, 1H), 7.21 (dd, J = 8.8, 2.2 1H), 7.21 (t, J = 18.7 1H), 4.89-4.80 (m, 1H), 4.16 (t, J = 9.1, 1H), 3.79-3.71 (m, 5H), 3.58-3.44 (m, 2H), 3.00-2.95 (m, 4H), 2.93 (s, 12H).

13C{1H} NMR (101 MHz, DMSO-d_6) δ [ppm]: 161.8, 155.0 (d, J = 243.5), 154.2, 136.2 (d, J = 8.9), 133.7 (d, J =10.6), 119.7 (d, J = 4.3), 114.6 (d, J = 2.9), 107.3, 107.0, 71.8, 66.6, 51.2 (d, J = 2.8), 47.7, 47.6,

19F{1H} NMR (377 MHz, DMSO-d_6) δ [ppm]: -121.2

HRMS (ESI-TOF) m/z: [M+H]+ Calcd for $C_{19}H_{29}FN_5O_3$ 394.2249; found 394.2245

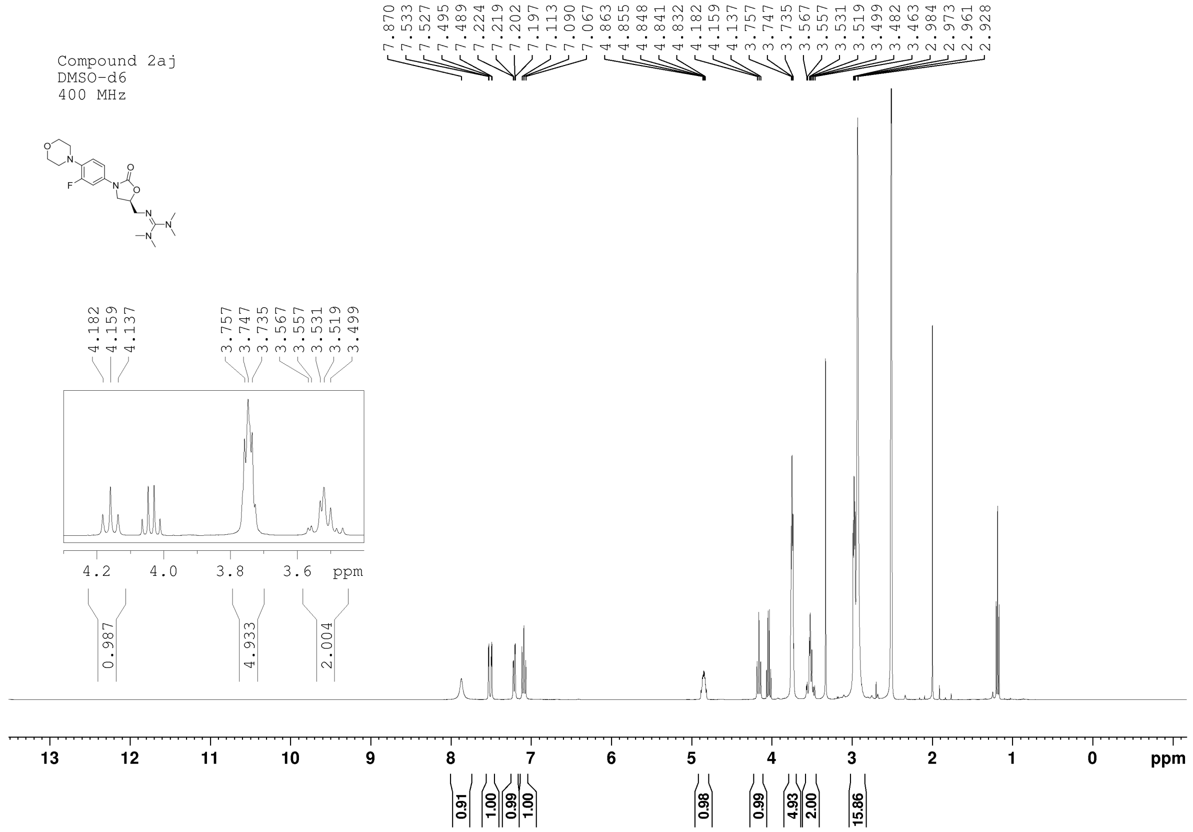

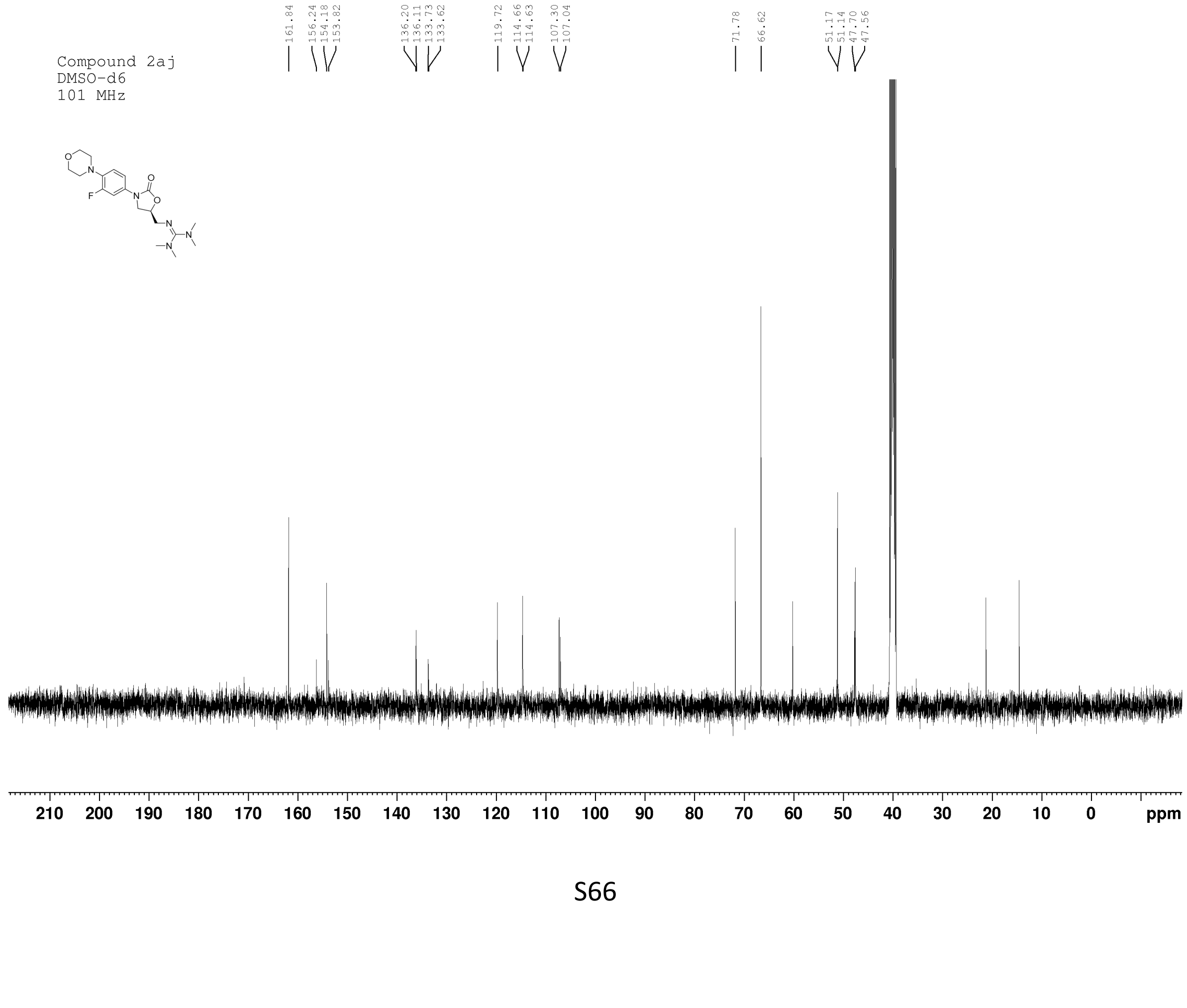

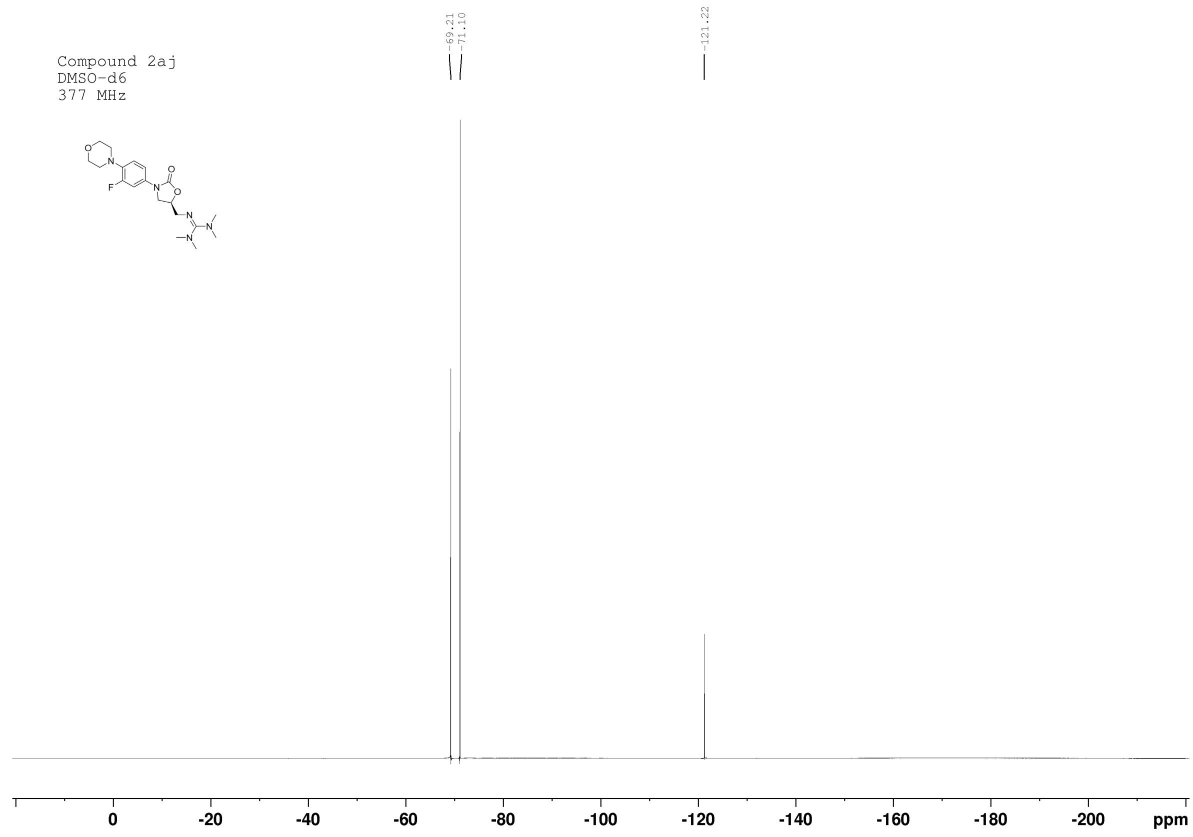

4a

4-fluoro-N-(4-fluorophenyl)benzamide

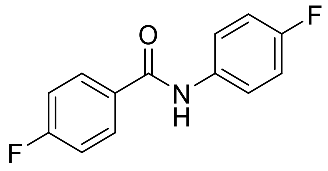

HATU (1.2 eq, 2.4 mmol) and 4-fluorobenzoic acid (1.0 eq, 2.0 mmol) was added into a 25 ml flask loaded with a magnetic stir bar. Dry solvent (8 ml of ACN) was added and the mixture was stirred. Lutidine (2.4 eq, 4.8 mmol) was added, followed by 4-fluoroaniline (1.0 eq, 2 mmol). Mixture was stirred overnight. Workup 3 was used with 1:1 cyclohexane:EtOAc as the solvent for silica gel chromatography. Beige solid. Yield 232 mg (50 %).

1H NMR (500 MHz, DMSO-d_6) δ [ppm]: 10.31 (s, 1H), 8.05 (dd, 2H, J = 8.5, 5.6), 7.78 (dd, 2H, J = 8.9, 5.1), 7.37 (t, 2H, J = 8.8), 7.20 (t, 2H, J = 8.8).

13C{1H} NMR (126 MHz, DMSO-d_6) δ [ppm]: 164.8, 164.6 (d, J = 249.0), 158.8 (d, J = 239.6), 135.9 (d, J = 2.5), 131.7 (d, J = 3.7), 130.8 (d, J = 9.1), 122.7 (d, J = 7.4), 115.8 (d, J = 18.4), 115.7 (d, J = 20.3)

19F{1H} NMR (470 MHz, DMSO-d_6) δ [ppm]: -108.8, -118.8

HRMS (ESI-TOF) m/z: [M+Na]+ Calcd for C13H9F2NONa 256.0544; found 256.0536

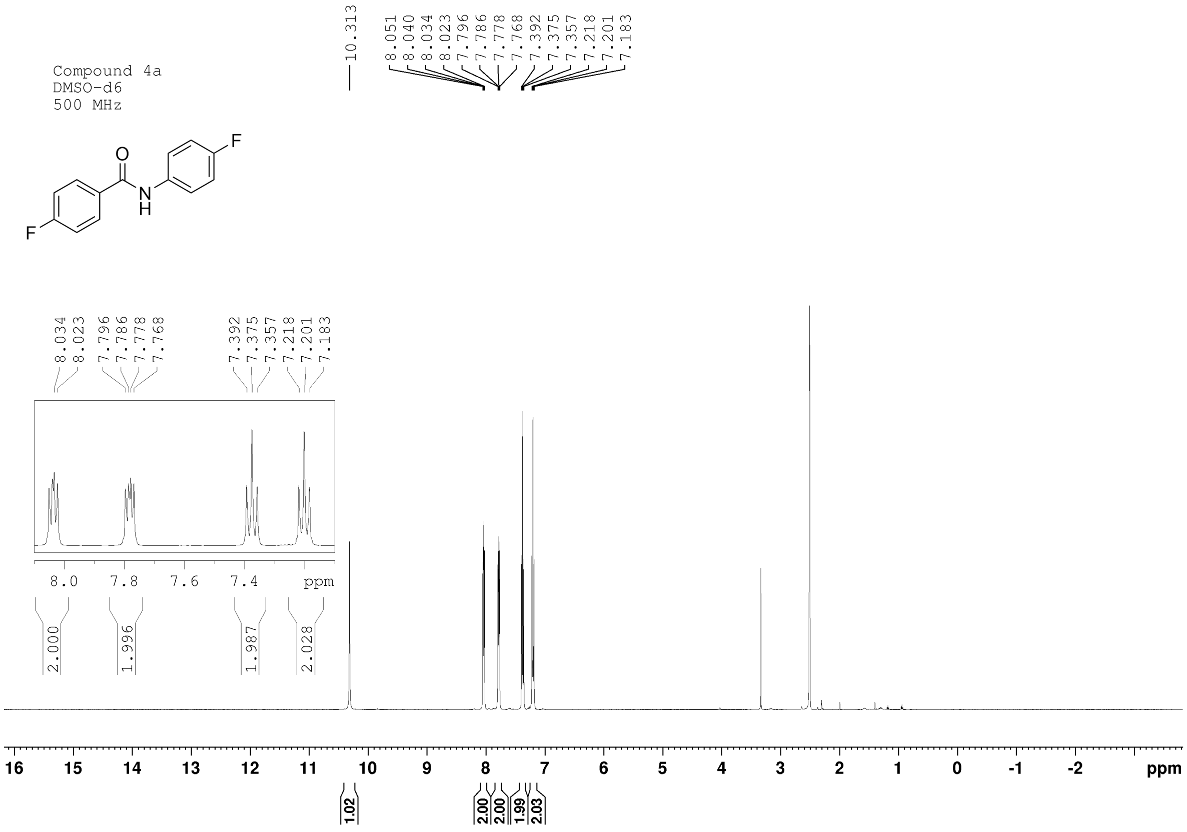
Compound 4a
DMSO-d6
500 MHz
10.313
8.051
8.040
8.034
8.023
7.796
7.786
7.778
7.768
7.392
7.375
7.357
7.218
7.201
7.183
2.000
1.996
1.987
2.028
1.02
2.00
2.00
1.99
2.03
ppm

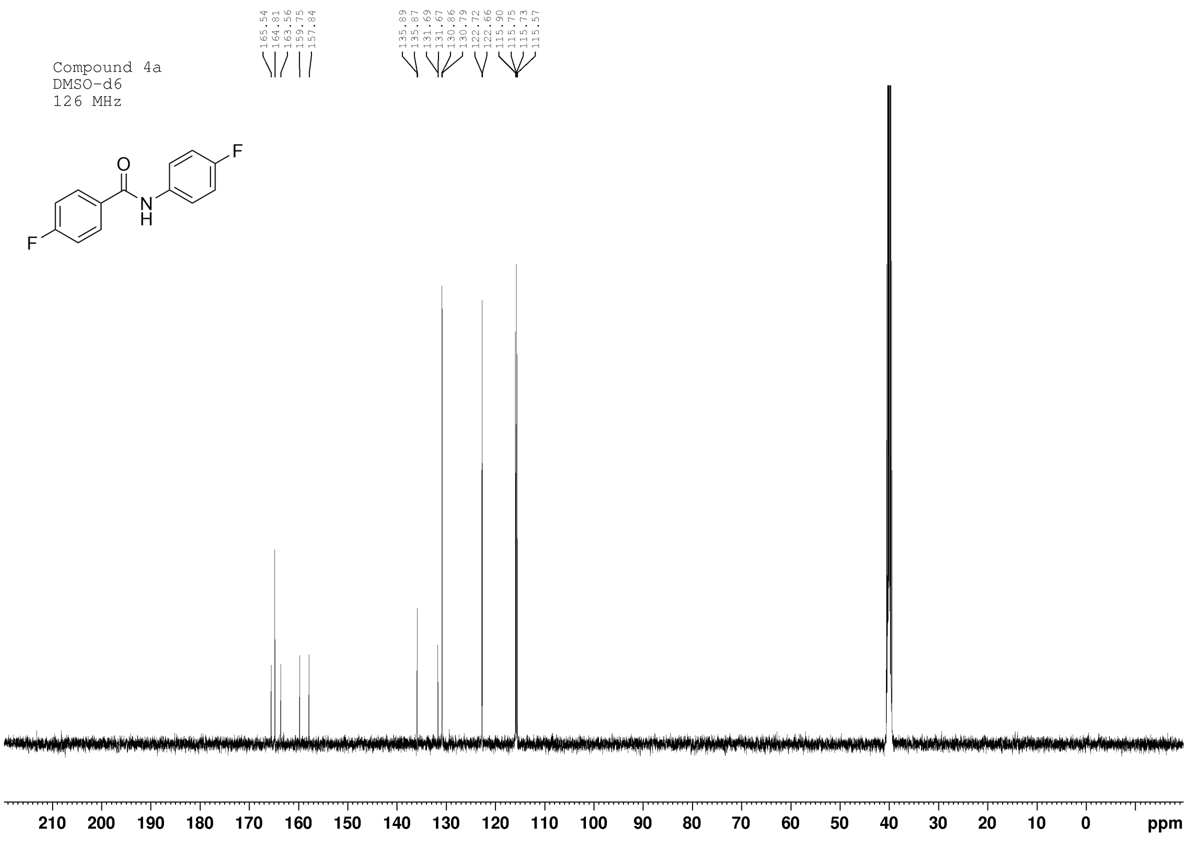
Compound 4a
DMSO-d6
126 MHz
165.54
164.81
163.56
159.75
157.84
135.89
135.87
131.69
131.67
130.86
130.79
122.72
122.66
115.90
115.75
115.73
115.57
ppm

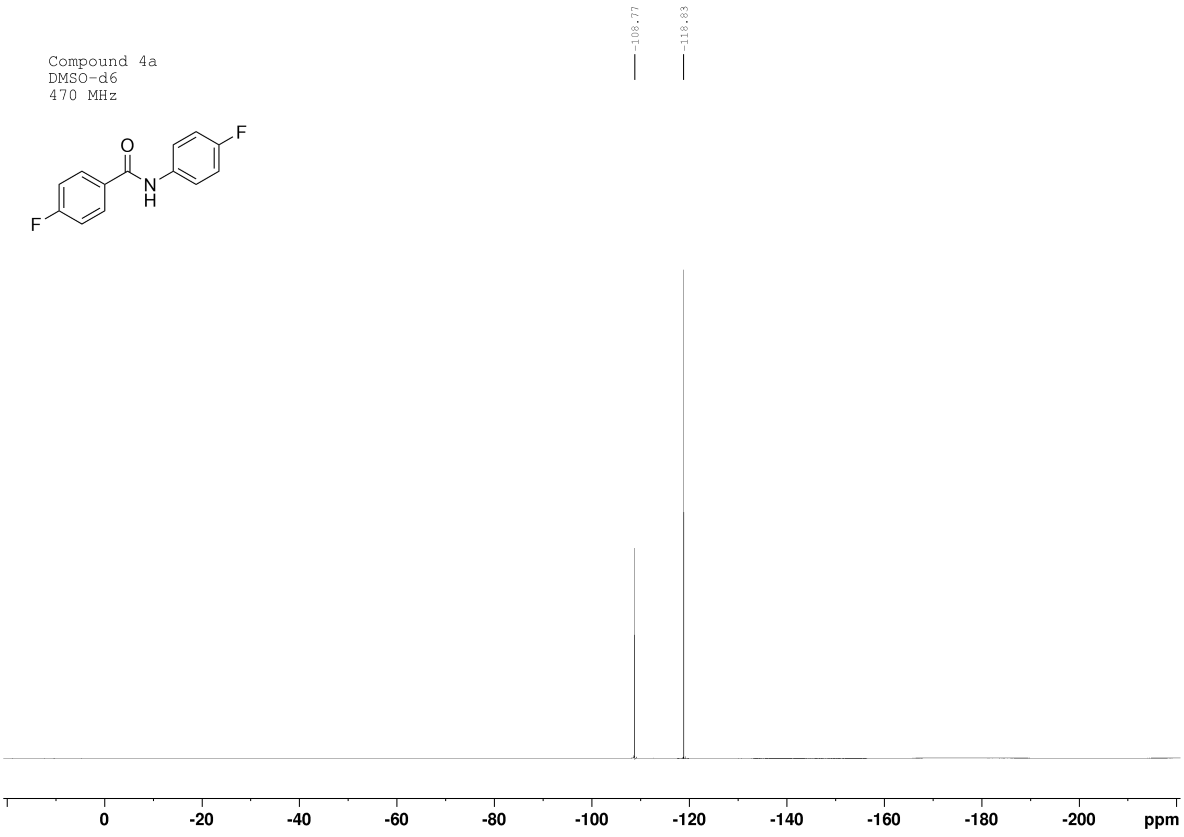
Compound 4a
DMSO-d6
470 MHz
-108.77
-118.83
0
-20
-40
-60
-80
-100
-120
-140
-160
-180
-200
ppm

4b

N-(4-fluorophenyl)-4-sulfamoylbenzamide

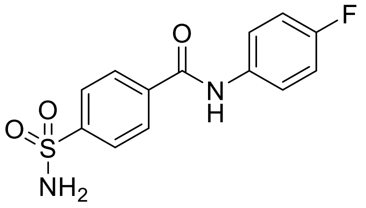

HATU (1.0 eq, 2.0 mmol) and 4-fluorobenzoic acid (1.0 eq, 2.0 mmol) was added into a 25 ml flask loaded with a magnetic stir bar. Dry solvent (8 ml of ACN) was added and the mixture was stirred. Lutidine (2.0 eq, 4.0 mmol) was added, followed by 4-fluoroaniline (1.0 eq, 2 mmol). Mixture was stirred overnight. During extraction with 2M NaOH and EtOAc, a solid formed. The solid was filtered and dried. Hot EtOAc was used to partially dissolve the solid and this was filtered. The filtrate was evaporated and a silica gel chromatography was performed using 90:10 EtOAc:MeOH to yield the product was a white solid. Yield 90 mg (15 %).

1H NMR (500 MHz, DMSO-d_6) δ [ppm]: 10.47 (s, 1H), 8.11 (d, 2H, J = 8.3), 7.96 (d, 2H, J = 8.3), 7.80 (dd, 2H, J = 8.9, 5.1), 7.53 (s, 2H), 7.22 (t, 2H, J = 8.9)

13C{1H} NMR (126 MHz, DMSO-d_6) δ [ppm]: 164.9, 158.9 (d, J = 240.6), 147.0, 138.2, 135.7 (d, J = 2.6), 128.8, 126.2, 122.7 (d, J = 8.3), 115.8 (d, J = 22.1)

19F{1H} NMR (470 MHz, DMSO-d_6) δ [ppm]: -118.5

HRMS (ESI-TOF) m/z: [M+Na]+ Calcd for C13H11FN2O3SNa 317.0367; found 317.0356

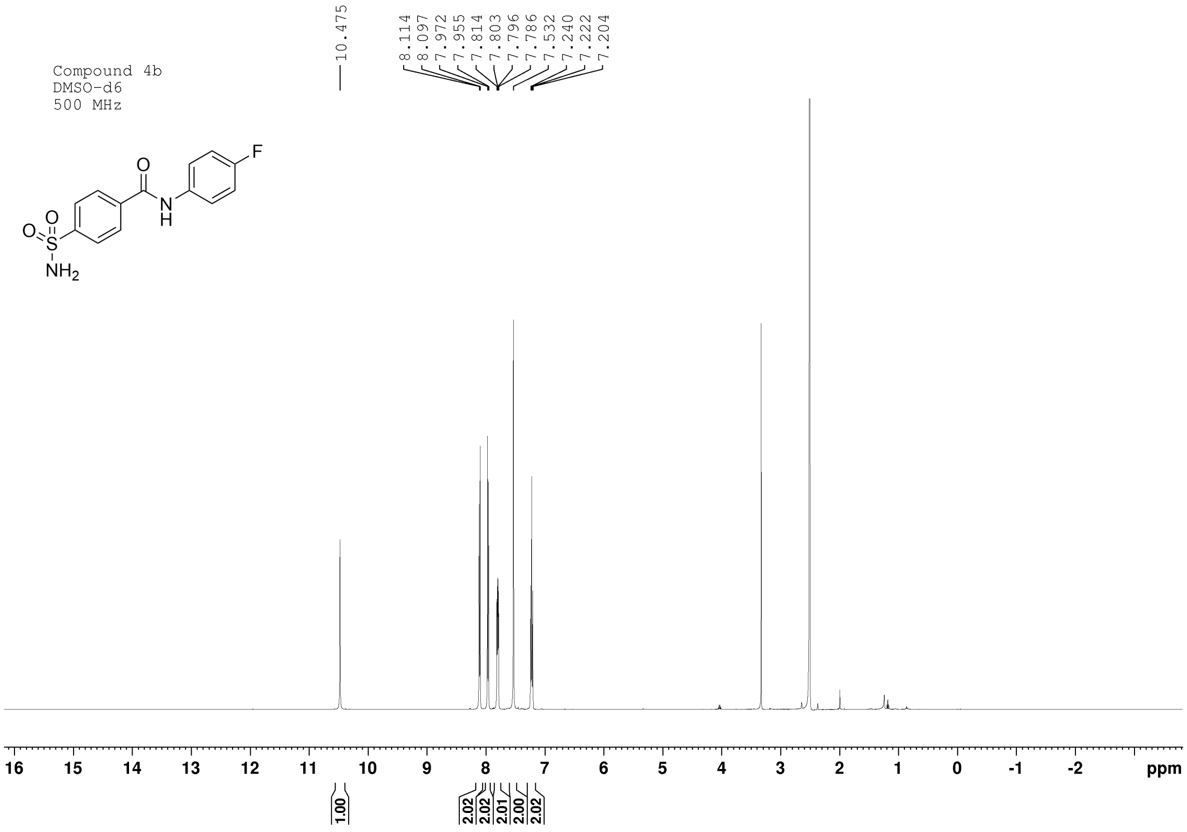
Compound 4b
DMSO-d6
500 MHz
10.475
8.114
8.097
7.972
7.955
7.814
7.803
7.796
7.786
7.532
7.240
7.222
7.204
1.00
2.02
2.02
2.01
2.00
2.02
ppm

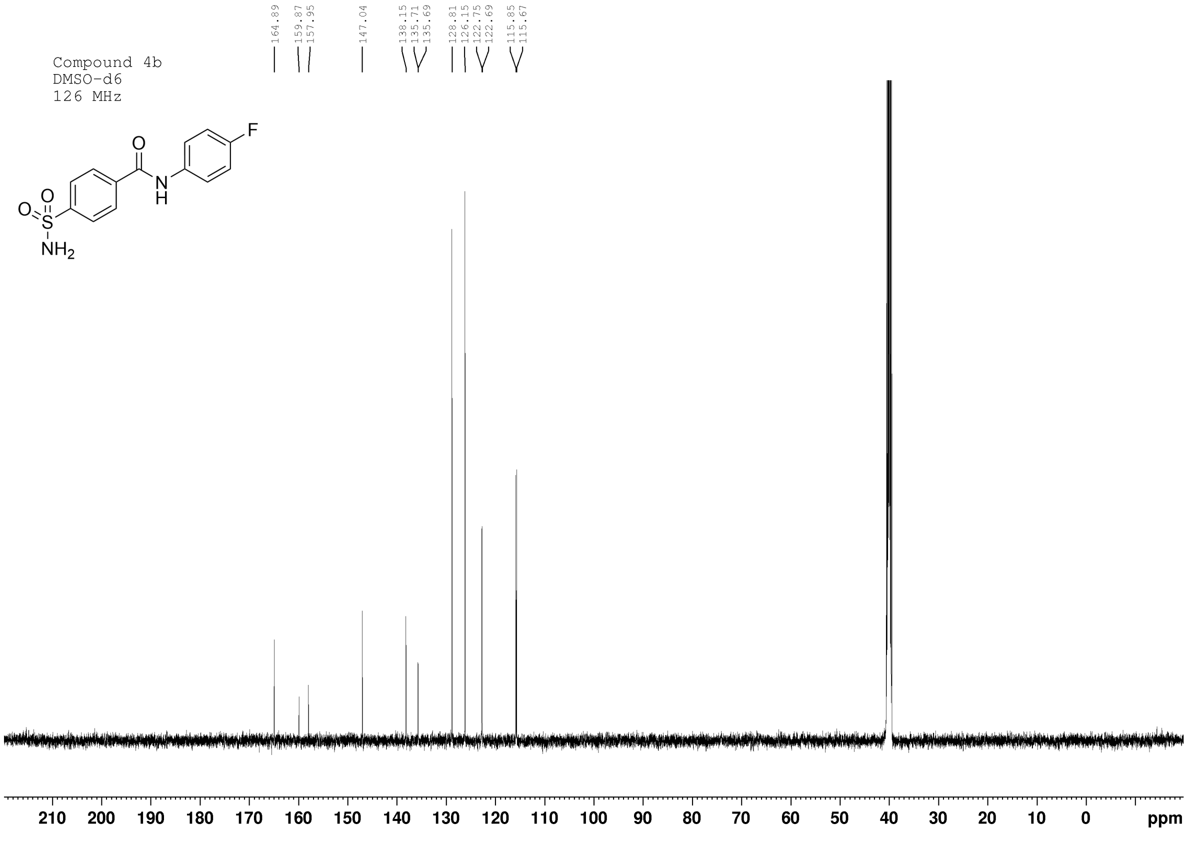
Compound 4b
DMSO-d6
126 MHz
164.89
159.87
157.95
147.04
138.15
135.71
135.69
128.81
126.15
122.75
122.69
115.85
115.67
ppm

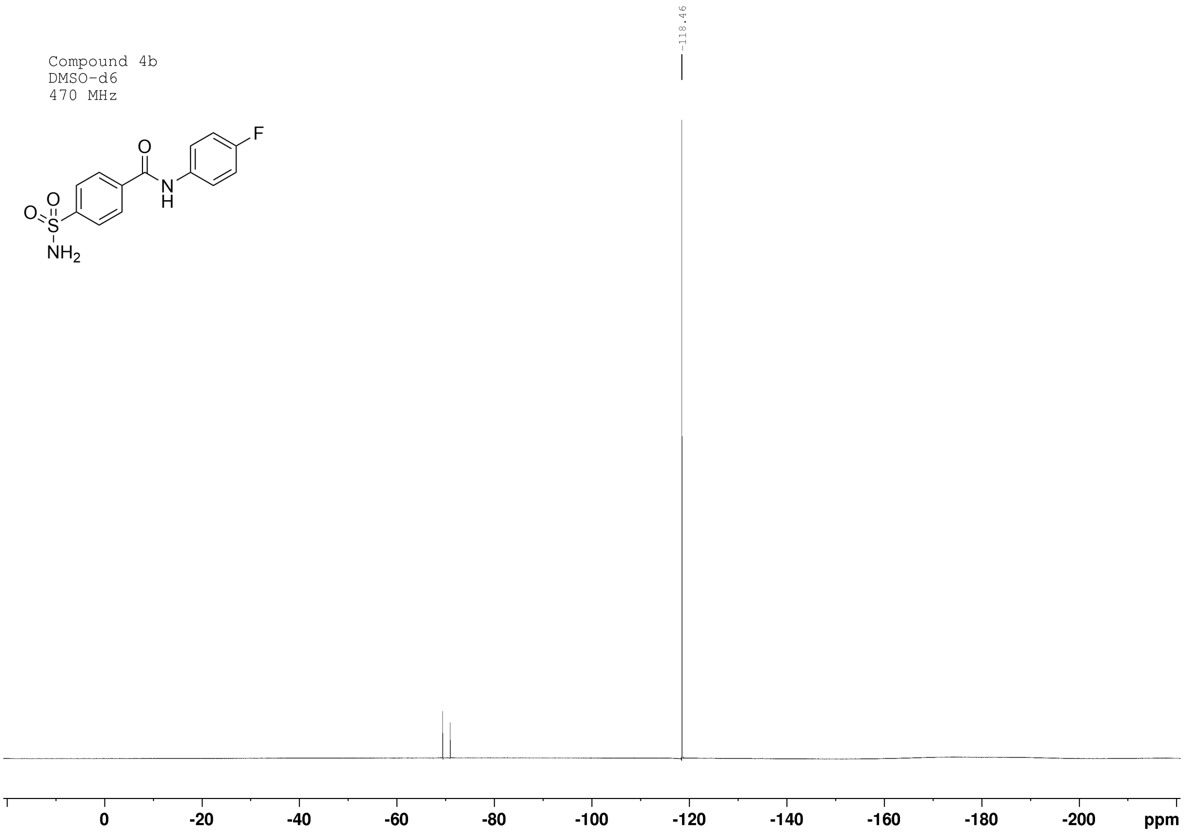
-118.46
Compound 4b
DMSO-d6
470 MHz
O
F
N
H
O
O
S
NH2
0
-20
-40
-60
-80
-100
-120
-140
-160
-180
-200
ppm

16. Competition experiment

Amide **4b** peak by $^{19}F\{^1H\}$ NMR is located at –118.10 ppm. Guanidine **2a**, most likely in protonated form, is located at –117.39 ppm in the lutidine experiment, whereas it is located at –118.54 ppm in the TEA experiment. The migration of guanidine **2a** peaks is also observable by ^1H NMR. In the lutidine experiment, the two doublet of doublets for guanidine **2a** are located at 8.77 and 8.52 ppm. In contrast, in the TEA experiment, the two doublet of doublets are located 8.47 and 8.25 ppm. The exact reason for the migration of peaks unclear, but it seems likely that guanidine **2a** is very sensitive to the hydrogen bonding environment.

The in situ competition reactions were conducted on 0.5 mmol scale in CD_3CN (2 ml). One complete, the reactions were diluted with DMSO-d6 for a final ratio of 1:5.

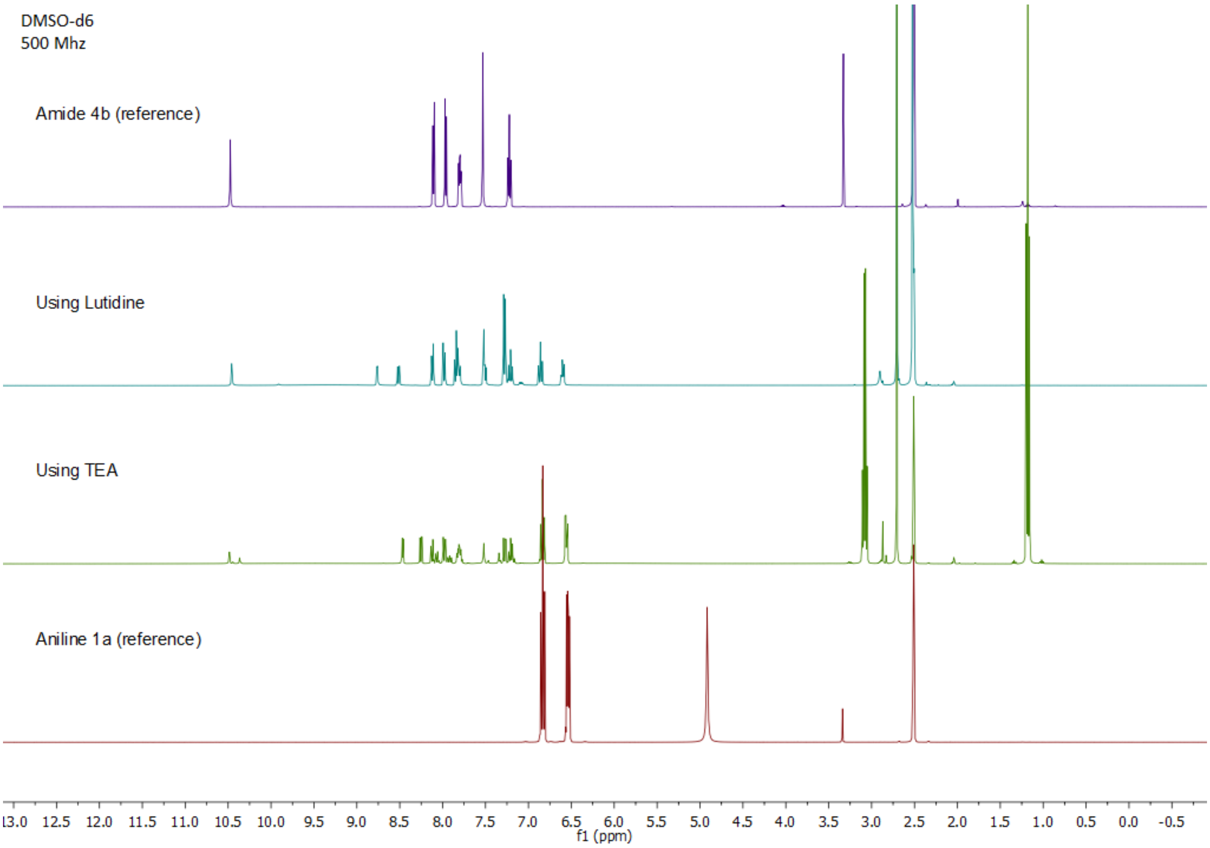

^1H NMR stacked spectra of the amide coupling presented in Table 5.

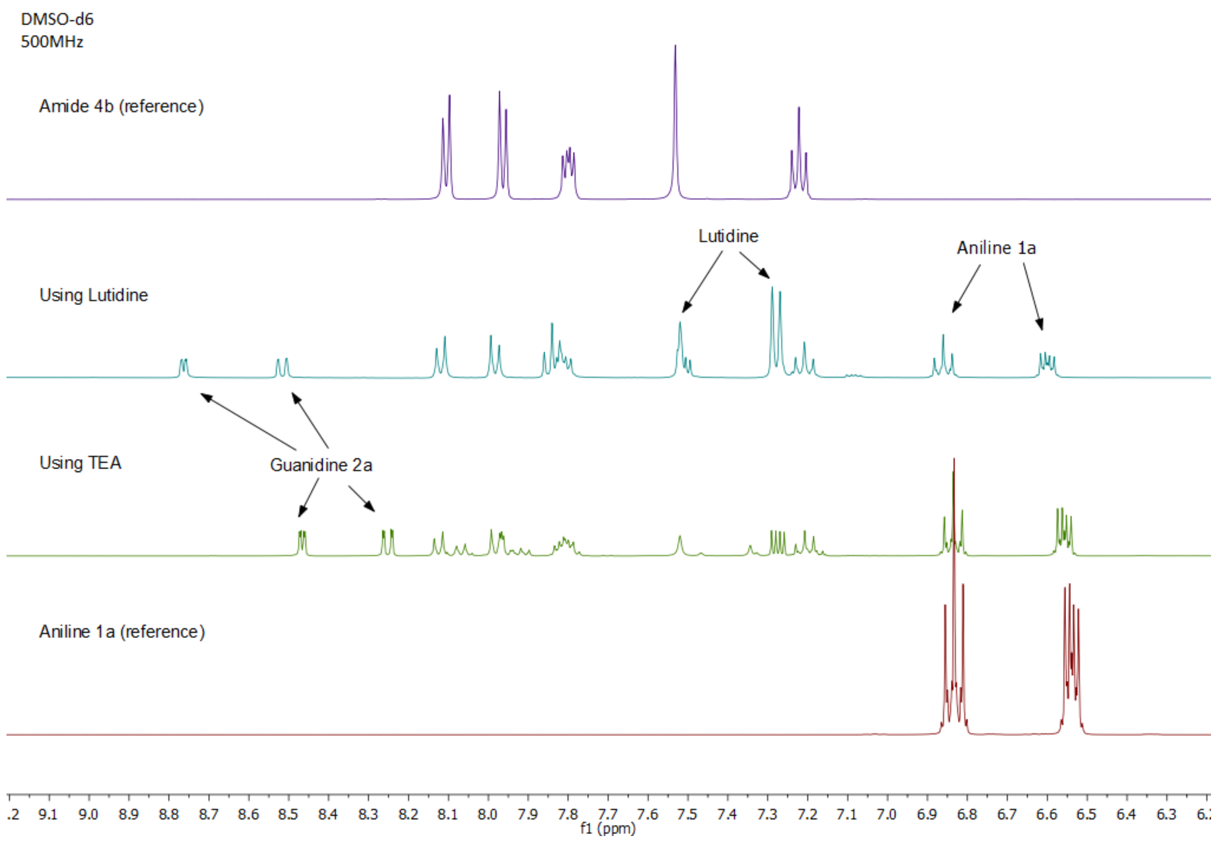

^{1}H NMR stacked spectra of the amide coupling presented in Table 5, selected region.

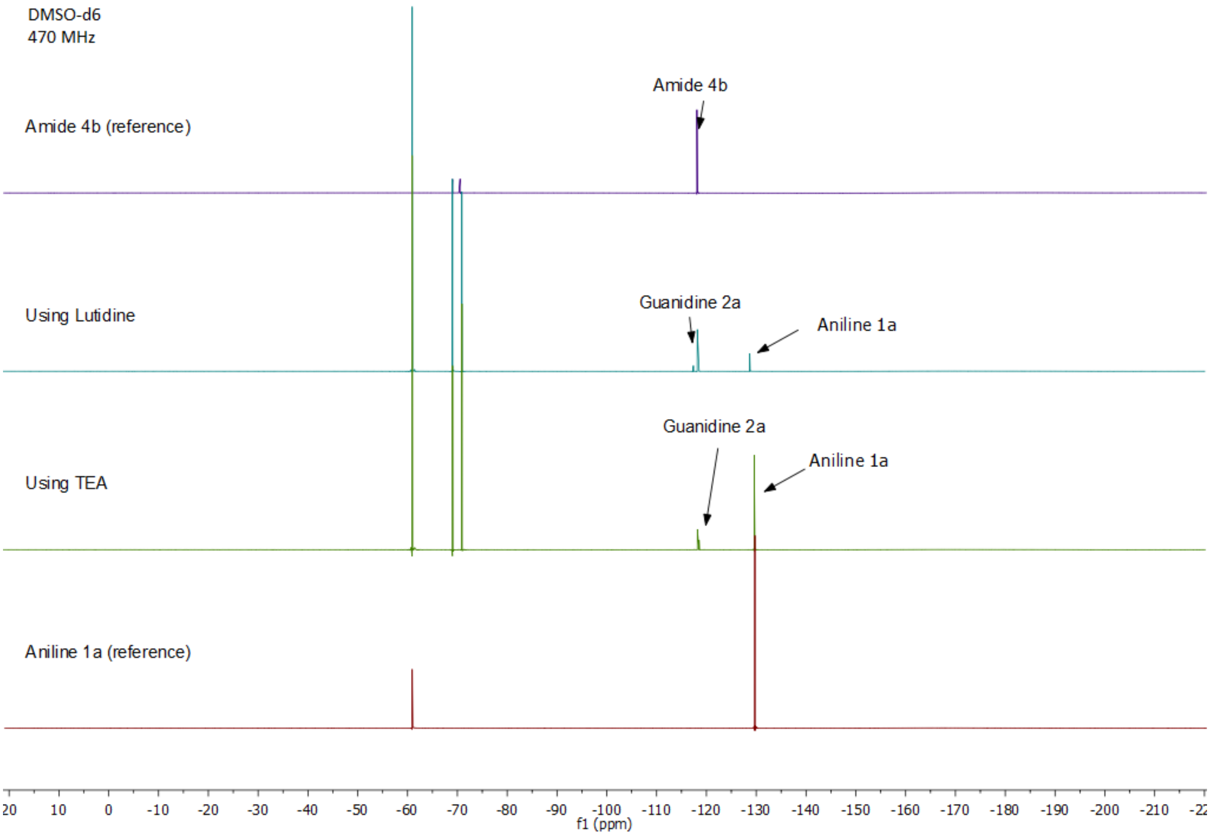

$^{19}F\{^{1}H\}$ NMR stacked spectra of the amide coupling presented in Table 5 with α,α,α-trifluorotoluene as an internal standard.

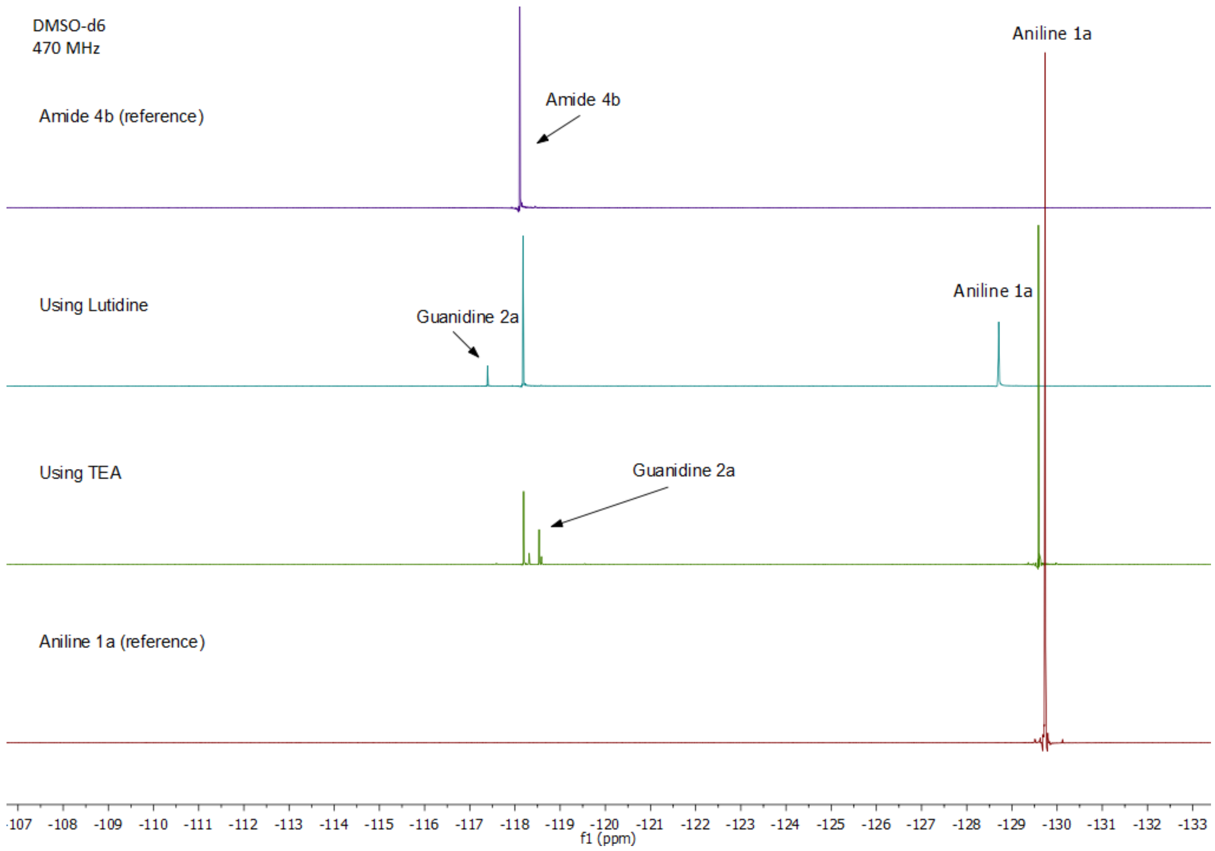

$^{19}F\{^{1}H\}$ NMR stacked spectra of the amide coupling presented in Table 5 with α,α,α-trifluorotoluene as an internal standard, selected region.
